# Barriers to integration of passive screening for sleeping sickness in Bibanga Health District, Democratic Republic of the Congo

**DOI:** 10.1371/journal.pntd.0014179

**Published:** 2026-04-08

**Authors:** Jérémie Ilunga, Philippe Mulenga-Cilundika, Joël Ekofo, Israël Badypwyla, Julienne Tshowa, Daniel Ishoso, Éric Mwamba, Gilbert Wembodinga

**Affiliations:** 1 Programme National de Lutte Contre la Trypanosomiase Humaine Africaine (PNLTHA-DRC), Kinshasa, Democratic Republic of the Congo; 2 Faculty of Medicine, Department of Public Health, University of Lubumbashi, Lubumbashi, Democratic Republic of the Congo; 3 Centre de Connaissances en Santé en RD Congo, Kinshasa, Democratic Republic of the Congo; 4 Faculty of Medicine, Université Nouveaux Horizons, Lubumbashi, Democratic Republic of the Congo; 5 Faculty of Medicine, School of Public Health, University of Kinshasa-Lemba, Kinshasa, Democratic Republic of the Congo; University of California Davis School of Veterinary Medicine, UNITED STATES OF AMERICA

## Abstract

**Introduction:**

The control of human African trypanosomiasis (HAT) relies primarily on the early detection and appropriate treatment of cases, which remain central components of current elimination strategies, while no vaccine or chemoprophylaxis is available. However, under-screening by mobile units is observed. Low HAT prevalence may lower motivation to participate in active screening programs that are important for early detection of cases, because people do not perceive the risk of infection. In this context, the integration of passive HAT screening into primary health services is encouraged but faces multiple challenges. This study aimed to explore the opinions and perceptions of the different actors involved in the integration of passive HAT screening into first level health establishments (Health Center and General Referral Hospital) in Bibanga Health District and identify the potential obstacles likely to affect it.

**Methods:**

A qualitative study was conducted in four health areas, selected based on their performance in reporting passive screening (PS) activities and their geographical distance from the Central District Office. Thirteen focus group discussions (FGDs) were held with men, women, and young people, as well as twelve semi-structured interviews (SSIs) with former HAT patients, community volunteers (Presidents of the Health Committee), healthcare providers, and local and provincial decision-makers. The discussions were digitally recorded, transcribed, translated from Tshiluba into French, and thematically analyzed using ATLAS.ti 7.5.16 software. A documentary review was also carried out to complement the empirical data.

**Results:**

Community perceptions indicate a limited knowledge about HAT, especially among young people, with confusion between HAT and malaria, fear of testing, and persistent rumors. Women expressed feelings of marginalization due to a lack of targeted information. On the provider side, demotivation is evident, linked to the absence of passive screening integration into official policies, lack of training, and lack of recognition. Community volunteer complained of a lack of support and motivation. The identified obstacles include: (i) structural barriers (geographical accessibility, stockouts, low attendance), (ii) economic constraints (medical expenses, transportation costs), (iii) sociocultural barriers (rumors, stigmatization), and (iv) institutional limitations (lack of supervision, low community involvement). Perception gaps have been noted between field actors and health authorities.

**Conclusion:**

The integration of passive screening into primary healthcare programs requires a multisectoral approach tailored to the local context. Simplifying the diagnostic process would facilitate integration. However, the healthcare system must be adequately funded and equipped. These results offer concrete pathways to enhance the integration of passive screening and contribute to the elimination of HAT by 2030.

## Author summary

Sleeping sickness (Human African Trypanosomiasis) has significantly declined in Africa thanks to decades of screening and treatment efforts. Today, the World Health Organization recommends integrating passive screening—detecting suspected cases within ordinary health facilities—as a key strategy to find remaining cases. However, in health zones like Bibanga, in the Democratic Republic of Congo, this approach remains challenging.

In this study, we collected the views of community members, former patients, nurses, and health authorities to understand why passive screening is not widely used. People expressed fear or distrust toward the test, health workers felt unmotivated, and necessary materials were often missing. These issues were observed even in health centers that were better equipped or located closer to district offices.

Our findings suggest that improving passive screening requires strengthening health communication, better training and support for health workers, and ensuring this activity is fully integrated into official supervision and reporting tools. Doing so would help protect at-risk populations and accelerate progress toward the goal of eliminating sleeping sickness by 2030.

## Introduction

The control of human African trypanosomiasis (HAT) relies primarily on the early detection and appropriate treatment of cases, which remain central components of current elimination strategies, while no vaccine or chemoprophylaxis is available [1,2]. Efficient control of this condition relies on the rapid identification of infected individuals, who are the main reservoirs of transmission [3]. In line with its recommendations, the World Health Organization (WHO) advocates for a combined approach of active and passive screening of at-risk populations, including asymptomatic individuals, to break the transmission chain [4,5].

Historically, sub-Saharan Africa has experienced several outbreaks of HAT throughout the 20th century, including a major resurgence in the 1990s. This resurgence peaked in 1998, after which strengthened commitments from WHO, national programmes, and NGO partners contributed to a steady decline in reported cases from the early 21st century onward [6,7]. Building on this progress, the 2012 WHO roadmap set the objective of eliminating HAT as a public health problem by 2020 and interrupting its transmission by 2030 [8,9].

The Democratic Republic of the Congo (DRC), the most affected country, still reports the majority of cases [10]. Despite intensive screening campaigns, DRC remains among the countries not yet eligible to request validation of HAT elimination, mainly due to the persistence of active foci and an still fragile surveillance system [9,11,12]. The Sleeping Sickness National Control Program (PNLTHA) in DRC bases its strategies on early detection and appropriate treatment of cases. Screening is performed using the CATT (Card Agglutination Test for Trypanosomiasis), rapid diagnostic tests (RDTs), and clinical examination (notably searching for cervical lymphadenopathy) [13]. However, the decline in prevalence has reduced the effectiveness of mass active screening. Passive screening, based on identifying suspected cases in healthcare facilities, remains inefficient due to low attendance at health centers and delayed care-seeking. Indeed, early symptoms (fever, headaches, fatigue) are non-specific and often mistaken for malaria, delaying diagnosis [4]. Patients tend to seek care only at advanced stages, which perpetuates the transmission cycle.

WHO recommends integrating passive screening into primary healthcare services by introducing RDT-HAT [14,15]. However, coverage of passive screening remains insufficient. A study by Falisse et al. (2020) in DRC highlighted the risk of underestimating endemicity. Given these limitations, a patient-centered approach is now recommended to optimize coverage and service quality [16,17]. This approach aligns with WHO’s strategy of integrated, person-centered care [17,18]. Nevertheless, this strategy faces significant challenges such as unequal access to care, lack of qualified human resources, and disruptions in supply chains [19].

In 2015, Mitashi et al. identified the absence of simple, adapted tools for diagnosing HAT as a major barrier to expanding passive detection in primary health centers [14]. Notable progress has been made with the advent of easy-to-store RDTs (up to 40°C) and affordable costs [3,16]. Many authors agree that integrating passive detection into the minimum package of activities of primary health centers offers a credible and complementary alternative to active screening [13,20]. However, low health center attendance remains a significant obstacle [14,20,21]. Between 2016 and 2018, a pilot project was conducted in three health districts of DRC (Yasa Bonga, Bibanga, and Kongolo), introducing an algorithm based on RDT-HAT [22,23]. The results suggest that a one-off project is insufficient: effective integration of passive screening requires a systemic approach rooted in local realities [24,25]. Ensuring universal access to passive screening for all at-risk individuals is essential to achieve the global goal of HAT elimination by 2030 [26].

In this context, this qualitative study aims to describe stakeholders’ perceptions and identify barriers to integrating passive screening activities for HAT into primary healthcare services in the Bibanga health district, Democratic Republic of the Congo. More specifically, it seeks to: 1) analyze the perceptions, knowledge, and attitudes of community actors, healthcare providers, and health policymakers regarding passive screening of HAT; 2) identify barriers hindering the effective integration of passive screening into health centers; and 3) develop operational and contextual recommendations to improve the sustainable integration of passive screening strategies in the fight against HAT during the pre-elimination phase.

## Methodology

### Ethics statement

The research protocol was approved by the Ethics Committee of the School of Public Health at the University of Kinshasa (ref: ESP/CE/161/2022). Informed consent was obtained verbally from all participants after explaining the study objectives, emphasizing the voluntary nature of participation, and ensuring guarantees of anonymity, confidentiality, and the right to withdraw at any time without justification.

### Study site and population

**Fig 1:**
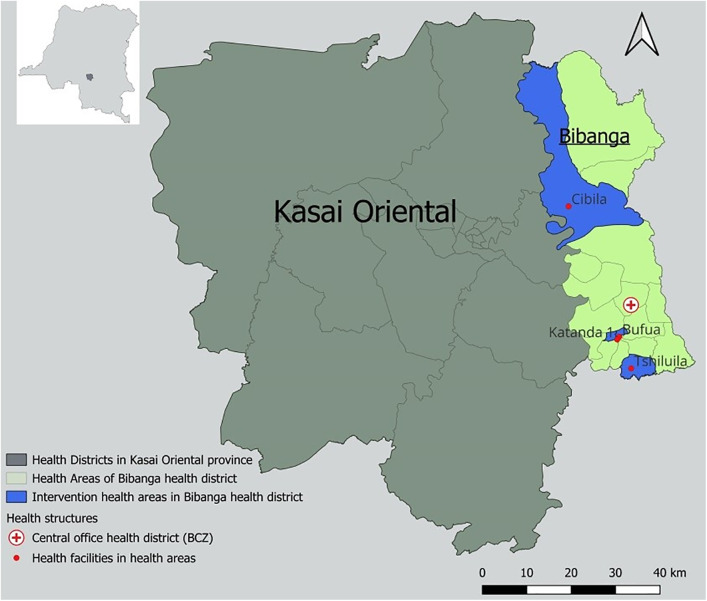
Map of intervention area. https://doi.org/10.5281/zenodo.17607958.

This qualitative study was conducted in the Bibanga health district, located in the Kasaï Oriental province of the Democratic Republic of Congo (DRC) (Fig 1). The area has a historically endemic presence of HAT and is surrounded by seven other zones also affected. The local health context is characterized by limited geographic and financial access to care, frequent shortages of essential medical supplies, and recurring community conflicts.

Four Health Areas (out of the 19 in the health district) were selected for this study based on their reporting performance levels (two high-performing and two low-performing) as well as their geographic location relative to the Health District Central Office (HDCO) (two close, two distant).

Participant recruitment was conducted using purposive sampling to ensure diverse perspectives and a rich collection of information. The study population included: 1) members of the local community and key informants (former HAT patients and community representatives), all residing in villages within the Bibanga Health Zone’s health areas that had recorded at least one new case in the past three to five years; 2) healthcare providers working in these health areas; 3) members of the Health Zone’s management team; and 4) provincial-level health decision-makers.

### Data collection methods

Between July and August 2023, two qualitative approaches were used to collect data: Focus Group Discussions (FGD) and Semi-Structured Interviews (SSI). We conducted 13 FGDs, each comprised of 8–10 community members. These included four groups of adult men (25 years and older), three groups of adult women (25 years and older), three groups of young men (15–24 years), and three groups of young women (15–24 years). The discussions were carried out until informational redundancy was reached, indicating data saturation.

The inclusion criteria for FGD participants were based on age, sex, and belonging to the local community. Participants were required to reside within the health area, fall within the defined age category (15–24 years or 25 years and older), and correspond to the gender targeted for each group. They also needed to be able to participate freely in group discussions and provide verbal informed consent. To minimize hierarchical bias or professional knowledge-related influence, health-care workers were not included among the FGD participants.

Simultaneously, 12 SSIs were carried out with key informants selected for the richness of their information. These included former patients, community relays (president of the Health Committee), qualified nurses, members of the health zone management team, provincial officials of the National Program for the Fight against HAT, and the head of the technical support office of the provincial health division.

Finally, documents associated with passive HAT screening tests were carried out at multiple levels of the health system: health centers, the Health District Central Office, and the Provincial Coordination of the National Program for the Fight against HAT. Reviewed documents included activity reports (2021–2022), monthly reports, supervision checklists, and reference and stock registers.

### Data analysis method

The qualitative data analysis followed an inductive thematic approach guided by the study’s objectives. An interview guide titled *“Analysis of perceptions and obstacles to the integration of passive HAT screening into primary healthcare services: a qualitative study in the Bibanga health district in DRC”* was used for all respondent categories (S1 Text). Audio recordings were fully transcribed and translated from Tshiluba into French. The transcripts were verified for linguistic and semantic fidelity by bilingual members of the research team. Each document was then imported into the qualitative analysis software ATLAS.ti 7.5.16 where it was stored and analyzed.

An initial open coding cycle identified units of meaning within respondents’ discourses. Descriptive codes were assigned to text segments related to perceptions of HAT including what it was, how people got it (mode of transmission), and symptoms; passive screening, including awareness of the practice and experience with it, and also perceived structural, economic, sociocultural, and organizational obstacles toward screening; healthcare-seeking practices; and the motivation of providers and community relays. Similar codes were grouped to generate analytical categories around the central themes of the study. These categories were compared across groups (FGD) and respondent types (SSI). This structuring allowed for the examination of points of convergence, divergence, or complementarity in the discourses, facilitating cross-analysis. Finally, triangulation was performed to ensure the validity and depth of the analysis, integrating data from interviews, focus groups, and official documents. Table 1 describes the data sources used (FGD, SSI, document review) for each of the themes that emerged.

## Results

### Study population

Focus group discussions (n = 13) included a total of 125 participants across four Health Areas, with 4 FGDs each carried out in Bufua, Katanda 1 and Bwa Tshiluila and a single FGD in Cibilia (Table 1). Overall, 53.6% of participants were older (≥25 years of age) and male. The majority of FGD respondents reported their occupation as farmer (51.2%), followed by artisanal diamond miner (17.6%, Table 2). Of the 12 SSIs conducted two were former HAT patients, three were Health Committee presidents, four were registered nursed and three health officials (Table 1). All SSI participants were over 25 years old, and the majority were healthcare professionals and male (Table 3).

**Table 1:** Number of respondent categories in the study by Health area and structure.

Categories	Bufua	Katanda 1	Bwa Tshiluila	Cibila	Central Office of the Health Zone	Provincial Health Division	Total
FGDs by category							
Community members	4	4	4	1	0	0	13
SSI by category							
Former HAT patients	1	0	0	1	0	0	2
Presidents of the Health Committee	1	1	1	0	0	0	3
Registered nurses	1	1	1	1	0	0	4
Health decision-makers	0	0	0	0	1	2	3

**Table 2:** Characteristics of Focus Group Discussion participants by Health Area.

Characteristics	Bufua	Katanda 1	Bwa Tshiluila	Cibila	Total
Age (in years)					
15 – 24	20	18	20	0	58
≥ 25	20	20	19	8	67
Gender					
Male	20	20	19	8	67
Female	20	18	20	0	58
Profession/occupation					
Student	4	5	3	3	15
Worker	3	3	2	1	9
Merchant	3	3	2	2	10
Farmer	13	14	17	20	64
Diamond miner	5	4	8	5	22
Unemployed	1	0	2	2	5

**Table 3:** Profile of SSI respondents by categories.

Characteristics	Bufua	Katanda1	Bwa Tshiluila	Cibila	Central Office of the Health District	Provincial Health Division	Total
Age							
≥ 25	2	2	2	2	2	2	12
Gender							
Male	2	1	2	2	2	1	10
Female	0	1	0	0	0	1	2
Profession/occupation							
Former HAT patients	1	0	0	1	0	0	2
Presidents of the Health Committee	1	1	1	0	0	0	3
Registered nurses	1	1	1	1	0	0	4
Supervisor (health district management team)	0	0	0	0	1	0	1
Provincial Coordinator Manager for HAT	0	0	0	0	0	1	1
Technical Support Office Manager	0	0	0	0	0	1	1

Four main themes emerged (Table 4). First is the perception of the different actors regarding sleeping sickness (HAT) which explores social representations of the disease, its image within the community, and the level of priority it is given by the population. Second was knowledge of the symptoms and transmission of HAT. This theme highlights the understanding that various categories of actors (community members and professionals) have of the clinical signs of HAT and its modes of transmission. Third is access to HAT screening and care which examines health care-seeking behaviors, passive screening practices, and factors influencing the utilization of health facilities. Fourth, obstacles to integrating passive screening into health centers. This theme analyzes structural, human, organizational, and sociocultural barriers that hinder the operationalization of passive screening in primary healthcare services. We present the FGDs, SSI, and document review separately.

**Table 4:** Summary table of the themes addressed.

Main theme	Data Source	Types of Actors Involved
Perception of sleeping sickness (HAT)	FGD, SSI	Community, providers, decision-makers
Knowledge of symptoms and transmission	FGD, SSI	Community, providers
Access to screening and care	FGD, SSI, Document review	Community, providers, zonal and provincial officials
Obstacles to integrating passive screening	FGD, SSI, Document review	All target groups

### Focus group analysis

#### Perceptions of sleeping sickness (HAT).

Perceptions and awareness of HAT depended greatly on the gender but not age of the FGD respondent. The majority of female participants were aware of the disease’s existence, but some perceived it as a mystical punishment or a consequence of prior bad acts behavior, *“When someone starts sleeping a lot, maybe it’s a spell cast on them,”* [FGD11_JF_BUF], *“We know that the fly bites, but some also say that witches transmit the disease.”* [FGD6_FA_KAT]. Unlike women, men perceived HAT more pragmatically and less mystically, *“Before, we thought it was a disease of witches, but now we know it’s the fly.”* [FGD_3_HA_KAT]. Some believed the disease mainly affects those working in forests or rural areas, *“This disease exists, but it mainly affects those who go to the fields or sleep outside.”* [FGD_2_HA_CIB].

#### Knowledge of symptoms and transmission.

Respondents accurately described disease symptoms (excessive sleepiness, memory loss, behavioral disorders), *“We see a person who begins to gain weight, no longer speaks normally, it’s as if they are possessed.”* [FGD_5_FA_BUF]. *“If you see someone talking to themselves and forgetting things, you should think of sleeping sickness.”* [FGD_9_JH_KAT].

Men were sometimes able to identify early non-specific symptoms as similar to malaria (or other illnesses), *“In the beginning, it’s like malaria, with headaches and fatigue. Later, you start sleeping too much.”* [FGD_1_HA_BUF]. It was also notable that among women there were misperceptions about the mode of transmission of HAT, *“If someone eats from the same plate as a patient, they can also catch the disease.”* [FGD_12_JF_KAT].

#### Access to screening and care.

Women rarely sought care as a first option and often expressed fear of the test. *“I’m afraid of injections and the test, especially when it involves a lumbar puncture.”* [FGD_12_JF_KAT]. In contrast, men were more willing to visit a health center than women, but many prefer to wait until symptoms worsen before testing. *“A man won’t go to the hospital for a simple headache. We wait to see if it passes.”* FGD_3_HA_KAT]. Both genders were concerned about treatment costs that emerged after diagnosis. *“We are told the exam is free, but then we are asked to buy medicines.”* [FGD_7_FA_TSH]. *“Tests are free, but then we are asked to buy more medicines, so we prefer to buy medicines at the pharmacy without going to the center.”* [FGD_11_JH_BUF].

#### Obstacles to integrating passive screening in health centers.

When asked about possible barriers to passive HAT screening at health centers, female focus group respondents highlighted the following: 1) many lacked awareness of screening tests and were not given clear explanations about them *“No, I’m not aware of the integration in terms of screening activities; all I know is that our AS refers, but I’m not aware of the screening itself.”* [SSI_2_PCO_KAT], 2) that the distance between villages where they lived and health centers and the associated transportation and other costs reduced access to HAT treatment, *“The health center is far, and sometimes we don’t even have money for transportation.”* [FGD_11_JF_BUF]. and 3) local beliefs and the influence of trad itional healers reduced program effectiveness. *“Here, before going to the hospital, we start with plants.”* [FGD_6_FA_KAT].

In contrast, male respondents showed a lack of interest in early detection and perceived that HAT only affects certain categories of people (farmers, fishermen, etc.). *“If you work in the city, you don’t even think about this disease. It’s for those who go into the bush.”* [FGD_3_HA_KAT]. But like females, men expressed concerns about test availability and treatment costs. *“You have to go far for the test, and often you come back with a prescription. That discourages us.”* [FGD_8_JH_BUF].

#### Suggestions to improve access to screening.

Female FGDs participants suggested that awareness could best be increased through action of influential women, including health care workers in villages, *“If nurses explained more often, we wouldn’t refuse screening.”* [FGD_6_AF_KAT]. Women also expressed that tests should be available at health centers without the need for referrals to other health facilities, and to remove the hidden costs associated with screening and treatment. *“We need to know that the test is truly free, and that we won’t be asked for anything afterward.”* [FGD_6_AF_TSH].

Male respondents suggested decentralization of testing to make it available in all health centers. *“If the test was done directly at the health center without transfer, more people would accept.”* [FGD_1_HA_BUF]. Men also felt that targeted screening campaigns would be effective *We often talk to women and children about malaria, but who talks to men about sleeping sickness?”* [FGD_9_JH_KAT]. Like females they saw the need to raise awareness of early symptoms to prevent diagnostic delays.

### Individual interview analysis

#### Perception of HAT and passive screening.

Presidents of health committees were aware of HAT but highlighted persistent beliefs that hinder screening efforts, *“Many people still think it’s a disease linked to spirits, so they prefer to see a traditional healer.”* [SSI_2_PHC_KAT]. PHCs emphasized the crucial role of community awareness. *“Our job is to explain to families that early testing can save lives.”* [SSI_1_PHC_BUF]. Former HAT patients provided firsthand experience with the disease and testing. They shared their own difficulties in accessing care before diagnosis. *“I waited too long before getting tested, and the disease had already advanced.”* [SSI_5_AC_TSH]. Former patients also confirmed that their treatment was effective, but that screening remains poorly understood within the community. *“Today, I am well, but many are afraid to take the test.”* [SSI_4_AC_BUF].

In contrast to community members, registered nurses (RNs) were aware of the importance of passive screening but highlighted a lack of resources in health centers. *“We received training on HAT, but we often lack tests and medicines.”* [SSI_9_IT_TSH]. Many RNs believed that the community still does not fully understand the importance of early screening. *“People come for other illnesses, but when we talk about HAT testing, some refuse, saying they are not concerned.”* [SSI_7_IT_CIB].

Local and provincial decision-makers recognize the importance of passive screening as a strategic lever in the fight against HAT, but their statements reveal a tension between theoretical commitment and operational constraints. At the local level, one Health District supervisor clearly expressed the team’s commitment to passive screening, emphasizing the need to facilitate early access to testing. *“We need to make screening more accessible to prevent patients from arriving too late.”* [SSI_10_SUP_BCZ]. At the provincial level, interest in passive screening is acknowledged, but logistical and funding issues are major points of friction. A provincial official warns: *“We still have logistical problems in transporting tests from the Provincial Health Division to health zones. I’m even shocked to learn that some rapid tests are still lying in storage and risk expiring.”* [SSI_12_MCP_THA]. Another Provincial Health Division official considers funding as an essential condition for feasibility: *“Without sufficient financial support, it will be difficult to scale up passive screening.”* [SSI_11_CB_DPS].

#### Obstacles to the integration of HAT passive screening.

##### Distrust of health centers and test results:

The issue of distrust of communities toward health centers and of lab test results received there were expressed by PHCs and RNs, respectively. *“Some believe that the tests themselves transmit the disease.”* [SSI_2_PHC_KAT]. *“When a positive result is announced, some patients ask for a second opinion elsewhere.”* [SSI_8_IT_KAT]. Related to distrust was the fear of diagnosis and stigma associated with HAT expressed by former patients. *“Many people think that the team comes with the disease to infect us, so they refuse to be examined.”* [SSI_5_AC_TSH]

##### Lack of awareness:

PHC members noted the lack of motivation among Community health relay agents who are volunteers, expressing the receive little support and this limits the ability to increase awareness of HAT passive screening. *“No transportation means, we can’t go to all villages to raise awareness.”* [SSI_1_PHC_BUF]. To counter this problem PHC members suggested strengthening the role of community relay agents in awareness campaigns by providing them with compensation for their work. *“If we had more support, we could convince more people to get tested.”* [SSI_3_PCO_BUF]. Former patients also suggested that they be included in awareness campaigns. *“If someone like me talks to others, they will listen.”* [SSI_5_AC_TSH]. RNs suggested the organization of awareness campaigns to demystify screening.

##### Fear of costs associated with testing and HAT diagnosis:

Similar to concerns expressed by FGD participants, former HAT patients emphasized problems associated with indirect costs associated with HAT treatment (transport, absence from work) as an important barrier to passive screening programs. *“I had nearly a month without working.”* [SSI_5_AC_TSH]. Former patients also suggested making screening more accessible, including in remote villages.

##### Lack of program resources:

Interviews with health professionals and officials focused more on programmatic barriers to screening efforts. RNs emphasized frequent stockouts of rapid tests (RDT-HAT). *“We had periods where we didn’t have HAT rapid tests for several months.”* [SSI_9_IT_TSH]. Nurses also mentioned a lack of ongoing training for nurses on screening and management. Zone and provincial decision-makers, emphasized the ack of inter-level coordination, faulty logistics in transporting rapid tests, and the lack of dedicated funding to sustainably support passive screening implementation in peripheral health facilities. Nurses’ principal suggestion was to regularly supply health centers with tests and medicines. *“If tests are always available and people understand their importance, they will come more easily.”* [SSI_9_IT_TSH]. Health officials all emphasized the need to increase investments in passive screening and develop partnerships with stakeholders to ensure regular supplies.

### Document analysis

Review of activity reports on passive screening, supervision records, and monitoring documents—were used to contextualize and support certain field observations. Passive screening activity reports showed considerable variation in frequency and quality. There were notable differences among the four health areas, consistent with their selection two high-performing areas, two low-performing areas, as well as two areas near the district’s central office and two located in remote areas. Thus, there were disparities in patient follow-up regularity and passive screening integration in official reports. We also observed discrepancies between provider statements and archives, with a mismatch of nurse reporting that they carried out passive screening, while their official reports contained no record of screening procedures or results. Passive screening was not included in supervision checklists, supporting provider and supervisor statements that passive screening is not systematically monitored or evaluated. Finally, we were able corroborate prolonged stockouts of rapid diagnostic kits (RDTs) and that several health areas experienced extended RDT shortages.

## Discussion

Through Focus Group discussions with community members as well as interviews with PHCs, complemented by individual interviews with former patients, healthcare providers and local and provincial decision-makers we have been able to describe perceptions of sleeping sickness (HAT) and passive screening, and more importantly understand the obstacles to and identify solutions for integrating passive screening into primary healthcare services. Overall, we found distinct perceptions between women and men. Women accept the existence of HAT but hesitate to get screened due to beliefs, fear of tests, and costs. Women emphasize the need for better communication and improved access to health services and screening at health centers to enhance the integration of HAT passive screening into health programs. In contrast, men recognized the disease, but mostly considered it a secondary issue, especially among farmers and artisanal diamond miners in rural areas. They exhibited a lack of interest in early detection, but they noted that barriers to accessing healthcare services hinder the integration of passive screening into programs and suggested targeted campaigns directed at men could improve the situation. Interviews brought other stakeholders into the discussion, principally health care professionals and officials. All actors recognized the importance of passive screening, but several obstacles remain (e.g., test stockouts, community mistrust, and resource shortages). Suggestions included improving awareness, supporting community relays, and ensuring a steady supply of tests are crucial for better integration of screening into primary health services.

### Perceptions of HAT and passive screening

Perceptions of sleeping sickness and passive screening varied among the interviewed actor categories, revealing sometimes contrasting interpretations. In the community, adults, especially women, recognize the seriousness of the disease because they have experienced it in the past, but they note a current decline in interest, linked to the rarity of cases. Some associate the disease with supernatural beliefs or external interventions (e.g., “the team that brings the disease”), which influences their willingness to participate in screening. This phenomenon was also observed by Mpanya et al. (2012) [27], who showed that risk perception is strongly influenced by collective narratives, disease visibility, and intergenerational memory. This aligns with findings by Theobald et al. (2017) [28], who demonstrated that the lack of gender-sensitive information strategies contribute to the exclusion of women from control interventions, limiting their access to screening and care.

Community relays (Presidents of the Health Committee), perceive HAT as a still real but forgotten threat. They highlight a lack of communication and mobilization around passive screening. They also note persistent confusion between old invasive treatment protocols and current, simpler approaches. This confusion acts as a barrier to acceptance of screening. Mulenga et al. (2019) emphasize that poorly understood or perceived screening can be associated with unjustified risks in rural communities.

Healthcare providers express a certain fatigue, considering the disease as “residual.” While recognizing the usefulness of passive screening, they mention feelings of abandonment, especially due to the lack of clear integration of this activity into routine care and monitoring tools such as the National Health Information System or supervision checklists. A study by Wamboga et al. (2017) [3] in Uganda’s pre-elimination region reports a similar trend, where the decline in cases led to reduced provider involvement in screening activities. Falisse et al. (2020) [16] also highlights that when the disease becomes rare, health professionals tend to prioritize more common pathologies, further decreasing vigilance regarding HAT screening.

Former patients see the disease as serious but report that fear of diagnosis and invasive old methods (notably lumbar puncture) still persist in collective imagination. However, they acknowledge that current treatments are simpler and more effective, which they believe should be more widely communicated. Mpanya et al. (2012) [27] also report that patients’ lived experiences are a key lever for improving community engagement when used in appropriate communication.

Local and provincial decision-makers recognize that HAT is no longer perceived as an immediate priority by both the population and health structures. This decreasing interest is attributed to the decline in cases, creating an impression of disease disappearance, as well as to relaxed communication efforts and the absence of an integrated alert system within primary health services. This situation leads to a loss of vigilance both in the community and among providers. As Wamboga et al. (2017) [3] note, in pre-elimination contexts, maintaining vigilance cannot rely solely on epidemiological memory; it must be supported by ongoing health education strategies and a strengthened screening system capable of detecting residual cases even with low incidence. The need for proactive communication also aligns with observations from actors in Bibanga, who lament a lack of community mobilization initiatives around passive screening.

These varied perceptions highlight the importance of an integration strategy for passive screening that considers social representations of the disease, the institutional positioning of screening, and the lived experiences of different actors. They call for enhanced communication activities, the rehabilitation of community memory regarding the disease, and clarification of passive screening’s role within primary care services.

#### Obstacles reported by women and young women.

Women identified several barriers to integrating passive screening: limited financial and geographic access, lack of information, and beliefs associated with testing. They expressed fear of the test, mainly due to confusion with lumbar puncture and concerns about side effects. Mpanya et al. (2012) [27] and Mitashi et al. (2015) [14] have shown that cultural beliefs and fears related to diagnosis can hinder participation in passive screening. These findings underscore the need for targeted, culturally sensitive communication strategies to improve women’s uptake of screening.

#### Obstacles reported by men and young men.

Among men, mistrust of the health system, fear of testing, and low awareness are the main obstacles. Some consider the test unnecessary or unreliable. Mpanya et al. (2012) [27] and Koffi et al. (2021) [29] mention this disinterest, often linked to low disease prevalence and lack of targeted communication. Acup et al. (2017) [30] note that in pre-elimination settings, the absence of sustained awareness campaigns reduces perceived utility of screening and fosters community mistrust. Strengthening awareness, emphasizing early detection, and restoring credibility of health structures are essential to address these barriers among men.

#### Obstacles reported by community relays (presidents of the health committee).

Community relays cite lack of material and financial motivation as a major demobilization factor. Mitashi et al. (2015) [14] show that community engagement cannot be sustainable without recognition. Wembonyama et al. (2015) [31] also highlights this structural barrier. Improving the structuring and motivation of relays is necessary to ensure their ongoing involvement in passive screening.

#### Obstacles reported by healthcare providers.

Providers identified three levels of barriers: institutional, operational, and human. They point to the absence of clear directives, shortages of tests, irregular supervision, and demotivation due to low incentives and case scarcity. Studies in the DRC confirm that provider motivation is influenced by working conditions. For example, Kabemba et al. (2021) [32] in Kalemie show that frustration over remuneration affects care quality. Similarly, Nkwahata et al. (2023) [33] in Kenge identify weak incentives and poor working environments as professional engagement barriers.

#### Obstacles reported by former patients.

Former patients mentioned fear of screening, lack of information, and negative perceptions of diagnosis. Mpanya et al. (2012) [27] and Mulenga et al. (2019) [15] highlight the importance of communication in shaping disease perception. Their experiences underscore the need for respectful, confidential approaches in screening activities.

#### Obstacles reported by local and provincial decision-makers.

The statements from decision-makers reveal a certain disconnect between the operational vision of field actors (regarding access and proximity) and the strategic perspective of provincial authorities (the lack of institutional ownership as well as the prioritization and resource allocation logic). This observation aligns with the findings of Robays et al. (2016) [34], who emphasize that decentralization of screening cannot succeed without close coordination between the central, provincial, and local levels. Similarly, Koffi et al. (2020) [35] stress the importance of institutional and logistical alignment to ensure the sustainability of integrated strategies to combat Human African Trypanosomiasis.

### Study limitations

One limitation of our study, was that it was conducted in a single health district (Bibanga), one of the three health districts that benefited from a pilot project to integrate passive screening into HAT programs. This choice, dictated by logistical, financial, and temporal constraints, limits the generalization of the results to other similar contexts in the DRC. We also acknowledge that for some interviews conducted with some healthcare providers, by the fact that the principal investigator is also a staff member of the National Program for the Fight against Human African Trypanosomiasis, may introduced a social desirability bias into responses. To mitigate this possibility, the study objectives were clearly explained to all participants, emphasizing the importance of freely and independently expressing opinions, outside any institutional ties with the National Program for the Fight against Human African Trypanosomiasis.

Finally, although the document review enriched the analysis, the study could have been strengthened by direct field observations or a formal assessment of the project’s implementation within the relevant structures. This additional data would have helped refine the interpretation of the results and further triangulate the information collected from participants.

## Supporting information

S1 File Verbatim Transcripts. (ZIP)

S2 File Verbatim Transcripts. (ZIP)

S3 File Verbatim Transcripts. (ZIP)

S4 File Verbatim Transcripts. (ZIP)

S5 File Verbatim Transcripts. (ZIP)
