# Supplementary material for: Barriers to integration of passive screening for sleeping sickness in Bibanga Health District, Democratic Republic of the Congo
Source: PLoS Negl Trop Dis. 2026 Apr 8;20(4):e0014179. doi: 10.1371/journal.pntd.0014179 (PMC13089886; doi:10.1371/journal.pntd.0014179)
Supplement: S1 File — (ZIP) [file pntd.0014179.s001.zip › S1_Verbatim transcripts/1_AS_BUFUA/AUD.1_FG_FEMMES_BUFUA.docx]

**FGD WITH MEMBERS OF THE BIBANGA HEALTH ZONE COMMUNITY**

**Audio N°1: FGD with women from the Bufua Health Area**

**I. Knowledge of Sleeping Sickness**

**Do you know a disease that causes a person to fall asleep uncontrollably at any time? What do you call it in your language? What are the different names for this disease, and what do they mean?**

*P3: Sleeping sickness*

*P5: Drowsiness*

*P1: Sleeping microbe*

*P9: It comes from microbes, and we know the disease comes from wild insects or bad flies that bite people. When it bites someone who has the disease and then bites another person, it transmits the disease.*

**Apart from the fact that a person has uncontrollable sleep at times, do you know other signs attributed to this disease?**

*P7: We know there are signs. You can see someone become paralyzed, lose consciousness; when they want to do something, they can't do it, but they start wandering around for no reason due to confusion. You can see in others headaches and high fevers.*

*P2: In others, there are cervical lymph nodes.*

*P4: In others, the body swells.*

*P5: In others, they sleep at any time. Sometimes there is loss of consciousness; everything they do or see, they feel tired, which shows they have sleeping sickness.*

*Where does this disease come from, and how is it transmitted to humans?*

*P1: It comes from the fly that comes from the forest. If it bites a sick person and then bites another, it transmits the disease to them.*

*P5: It is a fly different from the other flies we have.*

*P8: They are called cibuibua.*

*P2: It's when it bites a person who is sick and then comes to bite another who is not sick, it transmits the disease to them.*

**Are there ways to protect oneself from sleeping sickness?**

*P5: (......) Protect ourselves! Sir, it's like when we are in the field working, if this insect comes to bite you and quickly leaves, leaving you with the disease. Now, to protect yourself, what are we going to wear to chase away this insect? We see that traps have been placed by the river to catch flies.*

*P8: To my knowledge, to avoid this disease, only if the government thinks of us like in the past when they sprayed insecticide in the bush to kill the insects—that is the way to protect yourself. Because you can be standing like this, and this insect comes to bite you; now how are you going to protect yourself? Only if they use the products.*

**II. Perception of Health Services**

**What do you do here in the village when you feel sick? (Where do you go to find a solution?)**

*P3: We get examined; when the FEMETRO team comes, they do examinations.*

*P2: When I feel sick, I buy products, and if I take them and there's no change, I go to the health center.*

*P8: I go to the hospital.*

**When you think, based on the signs mentioned (reiterate some signs cited by the group), that a person has sleeping sickness, what do you do to find a solution?**

*P1: We tell them to go get examined at the hospital.*

*P4: Or else, when the person comes to the center and we find they are a suspect, we give them a referral letter to the secondary hospital.*

*P2: Or else, when the FEMETRO team comes for screening, we tell them to go get checked by the FEMETRO agents.*

**Do you know the structures that organize or carry out screening for this disease? If yes, which ones?**

*P4: They are down there at the secondary hospital. Hmm hmm, only at the trypanosomiasis unit is where they do sleeping sickness examinations. They are the only ones who do these examinations for us here. The others come from Ngandajika (FEMETRO).*

**How do you appreciate the services offered by the health center (CS) you attend in the village?**

*P5: The health center receives us well. You can come with a child; they welcome you well, treat the child, and the child heals. If it doesn't heal, they give you a referral letter and send you either to Bibanga or to the secondary hospital.*

*P8: We see that they help us because when we didn't have a center like this, if you had a child who was anemic, getting to Bibanga was difficult. But since they established the health centers, the nurse helps us. Even when we don't have money, he starts with the first treatment and then sends you to Bibanga for a transfusion.*

*P1: And when you leave the health center and arrive at Bibanga, you are always welcomed well.*

*P9: For me, we are received very well, as the mother just said.*

**How do you appreciate the distance to travel to reach the health center?**

*P3: Yes, some are far, but when a child falls sick, they must arrive at the health center for care. For some of us, we go from house to house to sensitize them to go to the health center; even if you have nothing, you must always arrive at the health center.*

*P5: It is very close.*

**How do you appreciate the waiting time before being received by the health center staff?**

*P6: You can go with the child to Bibanga. If you were given a note here, as soon as you arrive at Bibanga and present it, they don't delay you. They start treating the child directly; you can't even spend 30 or 15 minutes without the child starting treatment.*

*P5: When you come on the first day, you are always welcomed well. But the following day, when you come and find people, you have to queue. She repeated the same phrase.*

*P1: It depends on the number of people you find ahead of you. Because when you already find people before you, just as you were received the first time, you must respect the queue and take your turn.*

*P10: In short, when I have already committed to treatment, my goal is to continue. I have already left home for this; why should I complain? I wait until it's my turn.*

**How do you appreciate the treatment you receive at the health center?**

*P7: We are always treated well.*

*P2: We are always treated well, but if the nurse finds the disease is serious, he sends you to the competent structure.*

*How do you appreciate the availability of the health center nurse when you need them?*

*P1: At the health center, there is staff rotation; each nurse comes to cover their shift.*

*P4: There is staff rotation, but if a nurse wants to step out, we matrons are present. He can tell us, 'I'm stepping out to buy products'; he leaves and always returns on time. He never spends too much time away when he is on duty.*

*P9: When we come, we always find the nurse.*

**How do you appreciate the cost of consultation and care at the health center?**

*P10: The cost is reasonable.*

*P3: The cost is reasonable for everyone. The pricing is always for children, and for adults, it's for adults.*

*P4: The price... Previously, we saw people in households: someone with a sick child, a baby—the child is sick, but they keep them at home. You ask them, 'The child is pale, why don't you take them to the hospital?' They answer, 'Because I don't have the money.' That's why we tell them to always bring children to the hospital or to the health center since it's close. Afterwards, you can find money little by little to pay the debt. That's the advice we gave them.*

*P1: What we observe is that even if the cost is modest, when you arrive at the health center, they give you the consultation price and add a prescription to buy products, and you find that the product costs a lot. At that moment, it distresses the person accompanying the patient. Because when you get to the pharmacy and don't have the necessary amount to buy these products, you risk losing the patient.*

*P5: We wish that when we bring the sick person to the hospital, everything would be there. Because when we are sent to the market to buy products, no one knows us there; they don't even know the suffering you have. They tell you the product costs 13,000 francs, yet you only have the 3,000 francs you brought to the health center. At that moment, you will fail everything and risk losing the patient.*

*P8: What is important is that they provide the necessary products so that whenever we arrive, even if they ask for 10,000 francs, you can pay 5,000 francs upfront; they start the treatment, and you come to pay the rest later.*

*P2: We ask the government to help us with the products so that households, even if they don't have the means, can always receive treatment in case of illness to save lives.*

**Are you aware that sleeping sickness screening examinations are free?**

*P1: Yes, we know.*

*P3: Yes, we know that when someone has sleeping sickness, they pay nothing.*

**Is there a problem that prevents the community from attending the health center for care?**

*P7: Yes, there are people, sir. When someone is worried because they have no means to get treatment, thanks to sensitization, they go to the center, examinations are done, the disease is found. But when given the prescription, they go home and stay there because they have no money.*

**What is important is that we could provide assistance at the hospital so that the little money people give—the 2,000 francs there—could help purchase medicines and treat the patient. Now, when given the prescription, there aren't even products in the pharmacy. They might ask you for 5,000 francs, but the person only gives 1,500 francs. With that, how can the nurse buy the products?**

*That is the suffering. So the person prefers to stay home; they buy the products themselves at the pharmacy.*

*P3: It's the same suffering (collective response).*

**What are your suggestions if we need to improve access to health care services in our Health Area/Health Zone?**

*P4: That the authorities think of us, that they stock our health center with products. Because if there are products and I have a sick child, we can always treat them, and I can start paying gradually. But if I am given a prescription and sent to the market—there, no one knows me—I risk losing my child. That is the suffering.*

*P6: We ask that they stock pharmaceutical products.*

**III. Perception of Sleeping Sickness and Screening**

**How do you feel in the community if you are told that a certain person has tested positive for sleeping sickness after examinations?**

*P5: It worries us; it saddens us.*

*P1: Some, even if they have a positive diagnosis, refuse to take treatment. We tell them they are sick, and they run away because they are afraid of the treatment. Even if we encourage them multiple times, they still do not accept to take the treatment.*

*P8: We have concerns.*

*P10: It truly pains us.*

*P3: It pains me, and I feel like approaching them so they can receive the product.*

**To what do you attribute the fate/stigma of sleeping sickness?**

*P7: Previously, we were filled with false beliefs that if you got examined in the presence of a sorcerer, they would cast a spell on you to make you test positive. If you had 20 elements (white blood cells), it could increase to 100.*

*P2: For example, someone in my neighborhood when they had this disease, we thought they were a sorcerer, to the point of saying they had a 'crash' (mental breakdown) because they had behavioral confusion. And those who said these things were their aunts. He could even doze off in the toilet. Some said he was bewitched by his mother, yet it was sleeping sickness. When he was taken to the health center, he was screened for sleeping sickness. When he received treatment, the sorcery ended until today.*

*P8: It's like any other disease; you treat it, and it is curable.*

**Does sleeping sickness scare you when you hear about it?**

*P1: It used to be frightening, but since healthcare personnel were sent to us, the number of patients has decreased.*

*Do you think you would go to be screened at a health center/general referral hospital if you present signs suggestive of sleeping sickness?*

*P4: I am afraid, and I pray to God that I am not affected by this disease.*

*P2: I am always afraid. Recently, someone told me, 'How you are walking, it's as if you have sleeping sickness.' I said to myself, 'Why are they saying that? May it stay away from me.'*

**Why, according to you, are some people afraid to get screened for sleeping sickness?**

*P5: Because you see someone just starting treatment, even if they were strong, they weaken. Others become in poor health. There are even some who died just from taking the product, and others went mad. That is why, when you are told you have this disease, you become afraid, thinking, 'If I go, I will become like that person.'*

*P8: The fear also comes from the restrictions: when they say you must not eat this and not walk in the sun. They even say you must always stay in one place and not move.*

*P3: No, that's old. Now, patients are fine; they eat everything. Besides, they are taken care of.*

*P1: The fear is death. When announced that you have sleeping sickness, you think of death because many have died.*

**COMMENTARY**

The integration of HAT (Human African Trypanosomiasis) into our center is a good thing for the community because it helped us reduce the number of patients.

Furthermore, we see that our nurses are now more at ease; it's no longer like before when we sent people only to Bibanga, Tshikaji, and Mbujimayi.

We want them to add more diagnostic tools to eliminate this disease in Katanda.

**Thank you**
