# Supplementary material for: Barriers to integration of passive screening for sleeping sickness in Bibanga Health District, Democratic Republic of the Congo
Source: PLoS Negl Trop Dis. 2026 Apr 8;20(4):e0014179. doi: 10.1371/journal.pntd.0014179 (PMC13089886; doi:10.1371/journal.pntd.0014179)
Supplement: S1 File — (ZIP) [file pntd.0014179.s001.zip › S1_Verbatim transcripts/1_AS_BUFUA/AUD.2_FG_GARCONS_BUFUA.docx]

**FG WITH MEMBERS OF THE BIBANGA HEALTH ZONE COMMUNITY**

**Audio N°2: FGD with Adolescent Boys of the Bufua Health Area**

**I. Knowledge of Sleeping Sickness**

**Do you know a disease that makes the person who catches it fall asleep at any time and uncontrollably? What do you call it in your language? What are the different names for this disease and what do they mean?**

*P3: Sleeping sickness.*

*P7: It's still sleeping sickness.*

*P1: We know it as sleeping sickness.*

*P2: There is no other term.*

*P9: Also meningitis.*

*P4: The terms escape us; if there are other terms, they escape us.*

**Apart from the fact that the person has uncontrollable sleep at times, do you know of any other signs attributed to this disease?**

*P3: Yes, there is fever, headaches, and even sleeping.*

*P9: There are also lymph nodes that appear all over the body.*

*P4: Even on the neck too.*

*P2: Loss of consciousness.*

*P6: Someone who laughs in public at any time and for no reason.*

*P10: You can also see someone with paralysis of a lower limb.*

*P3: Well, for the signs, firstly there are headaches, sleeping, and fevers. If someone has these signs and contacts the mobile team that examines people, they will find this disease, that's how it is.*

**Where does this disease originate and how is it transmitted to humans?**

*P6: In the past, because this disease existed when we were still young and less intelligent, we saw the group that came to set up things in the forest to attract the insects that have it. We think it came from there.*

*P5: It comes from flies or mosquitoes.*

*P1: It's the fly.*

*P10: It's when this insect stings a person and then comes to sting another who did not have this disease.*

*P2: That is, the insect stings the one who has the disease and then stings the one who didn't have the disease to transmit it to them.*

*P4: It originates especially from flies and mosquitoes.*

*P5: It also originates from forest mosquitoes. When we go to the forest and these mosquitoes sting us, they also contribute to the transmission.*

*P9: Also, they place mosquito nets by the riversides, but we don't know where it originates from.*

**Are there ways to protect oneself from sleeping sickness?**

*P10: To protect oneself from sleeping sickness, since that insect stings, you must sleep under a mosquito net.*

*P6: It is also a matter of cleanliness; hygiene and handwashing are necessary.*

*P9: Also, as we have the habit of eating outside, the insect can sting someone in the forest and then come and land on the food; that could also transmit the disease.*

*II. Perception of Health Services*

**What do you do here in the village when you feel sick? (Where do you go to find a solution?)**

*P4: Others go to the hospital so that the disease can be detected quickly.*

*P2: Others go to the market and buy products from the pharmacy.*

*P7: As there are health centers near us, that helps us when someone feels sick; they go there to receive treatment.*

*P5: There are illnesses that just require taking some roots.*

**When you think, based on the signs mentioned (reiterate some signs cited by the group), that a person has sleeping sickness, what do you do to find a solution?**

*P10: We ask them to go to the hospital to know the causes.*

*P2: If they take products and there is no change, we tell them to go to the health center to get examined.*

**Do you know the structures that organize or carry out screening for this disease? If yes, which ones?**

*P8: Yes, there is a sleeping sickness post at the secondary hospital.*

*P5: It's at the secondary hospital.*

**How do you appreciate the services offered by the health center you frequent in the village?**

*P1: They always satisfy us.*

*P6: They satisfy us.*

*P4: They satisfy us because when we come, they give us treatment and we get cured.*

*P9: There is not always satisfaction because there are times when you are given products but there is no cure, and you start to regret the money you gave for the care, without any change.*

*P10: No satisfaction because sometimes you might lack money due to illness; you come to the health center and they refuse to treat you. The illness worsens and someone could die at that moment.*

*P3: They do not satisfy us at times. I give the example of staff who give a product that does not match your illness and causes other illnesses in the body.*

**How do you appreciate the distance traveled to reach the health center?**

*P2: Yes, because in every neighborhood there are posts. You can be in Bufua or Tshialama or Ngandajika, even in Katshia-Mpanga, you will always find a health center that is close to your residence.*

*P8: (......) it can also be due to the will of the person themselves who refuses to frequent the health center in their neighborhood, which is close, and prefers the one at a distance because there is quality care. They accept to travel this long distance because the center near them does not provide good care.*

**How do you appreciate the waiting time before being received by the health center staff?**

*P9: It's good because there is organization. When you come early, you are received first. If you find people who arrived before you, they won't let you skip; you have to queue following those who preceded you. When your turn comes, they will receive you.*

*P1: The waiting time also depends on the case. If you have a case that requires urgency, they receive you quickly. Sometimes if you have a high fever, the nurse gives you paracetamol to calm the fever, and when they finish treating the person they are with, then they will receive you.*

*P3: Depending on the larger number of people you find ahead of you, you have to wait and follow the order; it's not bad.*

*P4: Sometimes it can happen when there is only one nurse and many patients, then it takes us a lot of time and makes us click our tongues (in frustration). There also needs to be more nurses to speed things up.*

**How do you appreciate the treatment you receive at the health center?**

*P7: Yes, for me I am satisfied.*

*P9: For me, it depends on the nurse who receives you because some give injections well and others very poorly.*

*P4: For me, there is good treatment in our center.*

*P3: The treatment is good when there is a cure, but if there is no cure, how can I come back to the center?*

**How do you appreciate the availability of the health center nurse when you need them?**

*P7: Yes, they are available.*

*P5: Yes, they are available because they work in shifts; there are those for the day and those for the evening.*

*P8: On this side, there are no problems; the nurses are always there.*

**How do you appreciate the cost of consultation and care at the health center?**

*P3: ( ...) It bothers us because when you are sick, you arrive at the health center for care, they start asking you for a lot of money, and they even add a prescription that makes you go even more into debt.*

*P6: Sometimes it's because of the severity of the illness that they also ask for a lot of money. What can you do? You give it so that your sick brother doesn't die. That's why they ask for a lot of money.*

*P5: For me, I find the cost of treatment is acceptable. It's not the nurse himself who set it; it's the state that communicated it to him.*

**Are you aware that examinations for sleeping sickness screening are free?**

*P1: Yes, we know that.*

*P6: We have known that since our childhood.*

*P3: We know that the treatment is free, but when you are diagnosed positive, the treatment does not start immediately; it takes time to begin.*

**Is there a problem that prevents the community from frequenting the health center for care?**

*P3: The hindrance is caused by the lack of money. Let's take someone who has a fever and is also hungry at the same time. They must first go to the field to look for food because it's not normal to leave the household hungry and go anywhere else.*

*P10: The obstacle is also when a person has a fever but doesn't have the money for care. To be received there, you must first pay for the consultation. But if you don't have that money, what do you do? You stay home until you find the money.*

**What are your suggestions if we need to improve access to health care services in our Health Area/Health Zone?**

*P1: The obstacle; we want them to send us medications to the health center because what complicates things is when you are sick and you come for care, they prescribe a prescription for you to buy the products yourself.*

*P5: That they send products so that after examinations, if they discover the disease, they give the products; at that moment, the person is cured. But if they don't give the products, the illness worsens further.*

*P2: We notice that our center lacks products. If they want to do examinations on us, the only test is always the malaria test, the RDT. After doing this test, they prescribe for you to buy the medications yourself. These things do not facilitate healing.*

**III. Perception of Sleeping Sickness and Screening**

**How do you feel in the community if you are told that a certain person tested positive for sleeping sickness after examinations?**

*P7: I feel very bad; I ask myself the question to know where this disease came from.*

*P2: I have a feeling of fear because my friend, with whom we are together all the time, has just caught this disease, so it could also be transmitted to me.*

*P9: For me, it's joy because they discovered the disease quickly; otherwise, it could be transmitted to me if it drags on for a long time since I live with him.*

**To what do you attribute the affliction of sleeping sickness?**

*P6: Sleeping sickness is transmitted by insects when they sting a person. If there is treatment, it's good for the person to become normal.*

*P4: We are on earth, and on earth there are many problems. You can see a disease that is normal, but some people say it comes from darkness. However, there is also a hygiene problem; the disease catches you because you do not have adequate hygiene.*

**Does sleeping sickness frighten you when you hear about it?**

*P6: It frightens us because we think about what has already happened: a person who was fine with you and when they catch the disease, they start holding clothes in their hands, they go mad.*

*P9: It frightens because it causes sleep. You can see a person who was strong for work; they can no longer work, they start sleeping at any time.*

**Do you think you would go for screening at a health center/reference general hospital if you presented with signs suggestive of sleeping sickness?**

*P1: Yes, I have the courage to get screened for sleeping sickness.*

*P5: I would be afraid because of shame; they will ask me questions to find out where I got this disease.*

*P6: It frightens because it's not a good disease.*

*P2: We are used to doing it when the FEMETRO team passes; I have never been afraid.*

*P10: Getting tested is always frightening because when the FEMETRO team passes, they find the disease among people who didn't even know they had this disease.*

**Why do you think some people are afraid to get screened for sleeping sickness?**

*P5: The fear is when they think the disease comes from the injection, when you are pricked.*

*P9: For others, it's because this disease is shameful; so they are ashamed because people will mock them.*

*P4: Others also think about the harmful effects of this disease, but that was before; now the disease no longer has these effects.*

**Thank you.**
