# Supplementary material for: Barriers to integration of passive screening for sleeping sickness in Bibanga Health District, Democratic Republic of the Congo
Source: PLoS Negl Trop Dis. 2026 Apr 8;20(4):e0014179. doi: 10.1371/journal.pntd.0014179 (PMC13089886; doi:10.1371/journal.pntd.0014179)
Supplement: S1 File — (ZIP) [file pntd.0014179.s001.zip › S1_Verbatim transcripts/1_AS_BUFUA/AUD.3_ENT_PRESICODESA_BUFUA.docx]

**INTERVIEW WITH COMMUNITY LEADERS OF THE BIBANGA HEALTH ZONE**

**Audio N°3: Interview with the PRESICODESA of the Bufua Health Area**

**I. Knowledge of Sleeping Sickness**

**What is your opinion on the existence of sleeping sickness in your community?**

*In our health area, sleeping sickness exists and causes us suffering because it is one of the diseases that has exhausted people and made them suffer. When you are afflicted with this disease, it is a source of great hardship, especially in previous years, when you could remain on leave for six months without any activity, undergoing treatment (ombrage), eating, and washing yourself without being able to do anything else.*

**What are the signs that make you suspect sleeping sickness?**

*The first sign is that you will see the person sleeping deeply. The second sign is loss of consciousness. Another sign is that the person begins to gain weight, and their gait changes such that they are unable to walk normally. When we see these signs, we immediately suspect that the person has sleeping sickness.*

**Is there a treatment for sleeping sickness? (Is the disease curable?)**

*Sleeping sickness is curable; there is treatment available, and one can be cured.*

**Is your health center capable of screening for sleeping sickness?**

*Although the Équipe Cadre de Zone (ECZ) and the trypanosomiasis team have trained the staff, I observe that the health centers are not capable of diagnosing or treating sleeping sickness. They treat other illnesses, and only when there is no improvement do they refer the patient for sleeping sickness examinations at the trypanosomiasis center. That is where all the diagnosis and treatment take place. Here, we cannot detect sleeping sickness.*

**II. Perception of Health Services**

**When you feel ill, here in the village, where do you first go to seek a solution? (Church, traditional healer, or modern medicine?)**

*When I feel unwell, based on what we have learned, if I take treatment for even two days without success, I must go to the health center for examinations. If the illness is severe, they give me a referral letter for treatment at a larger hospital.*

**When you observe, based on the signs mentioned (referring to some signs cited by the group), that a person has sleeping sickness, what do you do to find a solution?**

*When I notice that a person is showing the signs mentioned above, as PRESICODESA, I will involve their relatives, the people living with the person in the same household. If it is the father showing the signs, I will address the mother; if it is the child, I will address the parents, asking them to bring the child to the sleeping sickness center. If they refuse, I must report the case to the health center.*

**How do you appreciate the services offered by the health center you attend in the village?**

*I appreciate the services at our health center because we do not always have money on hand. For example, recently when my wife was sick, I did not even have the money to take her for care, but in the evening I found that she had gone to the health center. When I asked her how she managed, she told me that she had explained to the nurse that I would come and pay, and they gave her treatment. Two days later, we found some money and paid part of the debt. This is why we encourage people not to stay at home when they are sick, and not to resort to self-treatment or traditional healers, because these actions can lead to other, more serious illnesses.*

**How do you appreciate the distance traveled to reach the health center?**

*The distance between home and the center is not excessive; it is acceptable for everyone in the health area.*

**How do you appreciate the treatment you receive at the health center?**

*The treatment at the center is very good, because even when you do not have money, you are always welcomed, and you are treated while you wait for the duration of the treatment. You can then look for money little by little and come to pay.*

**How do you appreciate the cost of consultation and care at the health center?**

*The cost of consultation is affordable.*

**Are you aware that screening examinations for sleeping sickness are free of charge?**

*I am convinced of this; I myself suffered from this disease when I was in my fourth year of secondary school (humanities), and I was treated free of charge.*

**Are there any problems that prevent community members from attending the health center for care?**

*The difficulty in the community is the lack of money. This constitutes a barrier to accessing care, despite the awareness-raising efforts to encourage community members not to delay at home with the illness. There are always those who say, "I cannot go because I don't even have a cent for the syringe; how will I settle the bill if they ask me for money?" But despite all this, there are always those who are understanding and others who are not. Some even go to the center but do not complete the treatment, stopping because the child has recovered and started playing. Then they return, pleading for the nurse to intervene again when the child has a severe form of malaria or meningitis.*

**What are your suggestions for improving access to health care services in our health area/health zone?**

*I recall a partner that supported the health zone with supplies. At the end of each month, the structure would provide something to the partner. At that time, there was a reduction in the cost of care due to the availability of inputs in the pharmacy. There were no problems with prescriptions or purchasing products on the market. Currently, the prescription system causes hardship for the community because the products are expensive. This is what frightens people and prevents access to care. If today there are still partners doing this, the RECOs (community relays) will raise awareness in the community, and these kinds of problems will no longer exist.*

**III. Perception of HAT Integration**

**Are you aware that the health center of this health area has integrated sleeping sickness control activities? If so, how did you receive this information?**

*Yes, I am aware because the IT (integrated technician) informed me that there was training for RECOs in Bibanga regarding fly trapping.*

**Why is the integration of sleeping sickness screening into this health center necessary for the community?**

*The integration is necessary because it involved the population in control and trapping activities.*

**Why, in your opinion, do some people choose to get screened for sleeping sickness elsewhere rather than here?**

*People still think about what this disease used to be called: the disease of sorcery. They believe that if you get screened in your village, the sorcerers in the village will cast a spell on you. That is why they used to go to get examined elsewhere.*

**In your opinion, what should be done to improve access to sleeping sickness screening through health centers?**

*It is awareness-raising. Through awareness-raising, we can instill this culture in the community.*

Thank you.
