# Supplementary material for: Barriers to integration of passive screening for sleeping sickness in Bibanga Health District, Democratic Republic of the Congo
Source: PLoS Negl Trop Dis. 2026 Apr 8;20(4):e0014179. doi: 10.1371/journal.pntd.0014179 (PMC13089886; doi:10.1371/journal.pntd.0014179)
Supplement: S1 File — (ZIP) [file pntd.0014179.s001.zip › S1_Verbatim transcripts/1_AS_BUFUA/AUD.4_FG_HOMMES_BUFUA.docx]

**FG WITH MEMBERS OF THE BIBANGA HEALTH ZONE COMMUNITY**

**Audio N°4: FGD with men from the Bufua Health Area**

**I. Knowledge of Sleeping Sickness**

**Do you know a disease that makes the person who has it fall asleep at any time and uncontrollably? What do you call it in your language? What are the different names of this disease and what do they mean?**

*P1: Yes, we know the disease called sleeping sickness, which when it infects someone, the person is always tired and sleeps a lot.*

*P3: No, there is no other name for this disease, only sleeping sickness.*

*P8: We have also heard of the disease of drowsiness.*

*P2: The meaning of these two names is: "sleeping" for sleeping sickness and "drowsing" for the disease of drowsiness.*

*P8: We say this because the person who has this disease dozes off at any moment and drools; it is that person whom we say has sleeping sickness.*

**Apart from the fact that the person has uncontrollable sleep at times, do you know any other signs attributed to this disease?**

*P3: The signs we see are that the person has red eyes, and their gait is not dynamic—that is, they are tired. Since we know this, if we see such a person, we take them to the health worker. They have red eyes with blood in them.*

*P7: We see other people with a large number of stretch marks on the body, and the person scratches themselves a lot. If that person goes to the health center, they must be tested for sleeping sickness.*

*P5: As has just been said, if someone has this disease, we see stretch marks everywhere and the body swells; they scratch all over their body, and this happens when the person has been bitten by the insect of sleeping sickness.*

**Where does this disease come from, and how is it transmitted to humans?**

*P6: Sleeping sickness comes from the bite of flies when you walk through dense areas in the forest.*

*P10: That fly is an insect that flies quickly. If it bites you, you will feel that your body is no longer normal; you get goosebumps and a tingling sensation at the bite site, and that is when you catch the disease.*

*P5: This disease comes from this fly, which is the vector. It is called the tsetse fly. If it bites a sick person and then bites another, that is how the disease is transmitted.*

*P2: It is transmitted within households when we have bedbugs in the home. They bite one person after another, or even mosquitoes do.*

**Are there ways to protect oneself against sleeping sickness?**

*P5: Yes, such means exist. We are given mosquito nets to catch these flies. We have been taught that when the flies are caught, we take them to the center. Currently, due to a lack of these nets, there is a proliferation of these flies, and even in our homes, since there are no more nets, there is a proliferation of mosquitoes that bite us. If they bite a person with this disease, then all of us in the house are at risk of catching it.*

*P9: Yes, we can avoid this disease through environmental hygiene: clearing our surroundings, avoiding stagnant water puddles, and keeping the area around our showers clean.*

**II. Perception of Health Services**

**What do you do here in the village when you feel sick? (Where do you go to find a solution?)**

*P2: In my neighborhood, we have solidarity. Even if the parents are absent and a child falls sick, we take that child to the health center for care. We tell the nurse to start treatment, and when the family arrives, they will have their money. We have no delays in this regard.*

**When you think, based on the signs mentioned (recall some signs cited by the group), that a person has sleeping sickness, what do you do to find a solution?**

*P10: First, among the signs, there is also the cervical lymph node. We even see when the FEMETRO team passes through the village, they palpate the neck to look for lymph nodes. Then, when a person presents the signs, we take them to the center for examinations to confirm whether it is this disease.*

**Do you know the structures that organize or conduct screening for this disease? If so, which ones?**

*P4: In our area in Katanda, we have a sleeping sickness center for FOMETRO. That is where all examinations and treatment for sleeping sickness are done.*

*P9: There is also the Bibanga hospital; that is where we used to go before for sleeping sickness. Now, even Bibanga sends people here to Katanda to the FOMETRO center.*

**How do you assess the services offered by the health center you attend in the village?**

*P1: No, we cannot give false comments. The health center in the BUFUA neighborhood—I am the neighborhood chief speaking to you—this health center welcomes everyone well. Even if you do not have a single franc, you are treated first, and the money comes later.* *There is no difficulty.*

*P3: Yes, we are satisfied with the services offered.*

*P8: Yes, the service with our nurse is as if we were family.*

**How do you assess the distance to travel to reach the health center?**

*P1: On this side, there are no problems; the distance is not great for the population of the BUFUA neighborhood; everyone always comes here.*

*P5: Yes, it is as he said.*

**How do you assess the waiting time before being received by the health center staff?**

*P10: The waiting time poses no problem at our health center. For example, if a visitor comes, they are received very well, and they will not be delayed.*

*P7: As he just said, we receive the sick visitor first before attending to others.*

*P5: When there is an emergency case, we take care of that case first. Moreover, when there is a serious case, we ask another nurse to attend to it, and the work goes very quickly.*

*P9: When we come to the center for treatment, it goes very quickly. No one is kept waiting.*

**How do you assess the treatment you receive at the health center?**

*P4: When we come to the center, the nurse's job is to perform examinations. If he discovers the disease, he provides treatment. Healing is up to God, because we see people go even to the secondary hospital or to Bibanga; there are doctors there, but sometimes people die.*

*P6: It is as he said: our nurse treats well. If he sees that the case is serious and beyond his capacity, he refers the patient to the hospital where there are doctors.*

**How do you assess the availability of the health center nurse when you need them?**

*P1: In BUFUA, we do not worry about that. Our staff is always available, which even attracts patients from other neighborhoods to come here.*

*P2: We have several nurses here. Whatever time you come, you will always find a nurse working.*

**How do you assess the cost of consultation and care at the health center?**

*P3: With us, whatever you bring, you are received first; whether it is little or a lot, you are received first.*

*P1: I first thank God for this work here. As has just been said, we receive the sick person first even if they do not have the money. It is only afterward that the patient's family comes to pay, whether it is 3000 or 4000 francs. But the essential thing is that the patient is cured.*

*P10: I reiterate what the chief just said: the price is affordable, but it also depends on each person's means. You might see that they ask for 4000 francs required for the child's treatment, and the person starts lamenting in front of the nurse. Since the nurse knows they are our regular patients, he will give the treatment, and afterward the person will come and pay little by little until they finish both the treatment and the debt.*

**Are you aware that sleeping sickness screening examinations are free?**

*P9: We know; it is free.*

*Is there any problem that prevents the community from attending the health center for care?*

*P9: Yes, lack of money constitutes a barrier to attending the health center.*

*P6: Financial impossibility prevents people from getting care. Some people even pawn their belongings to receive care; all this is due to lack of means.*

*P3: We have already seen someone brought to the health center in critical condition. When asked, "All this time, where were you?" they reply that there was no money for care, which is why they were treating them at home with traditional remedies.*

*P7: There is no other barrier than money, because if we talk about transport, there is no distance of more than a 30-minute walk to reach the center. At least if the patient has difficulty walking, otherwise there is only the problem of money.*

**What are your suggestions for improving access to health care services in our Health Area/Health Zone?**

*P5: We want you to think of us, to buy products for our pharmacy.*

*P2: That they supply our pharmacy with products.*

*P10: We want products, because often it is the prescription that makes people flee. You come, they ask you for 4000 francs, and then they give you a prescription for 25,000 francs.* How can someone bear that?

**III. Perception of Sleeping Sickness and Screening**

**How do you feel within the community if you are told that a certain person has tested positive for sleeping sickness after examinations?**

*P2: It makes me very sad, despite progress in care. We see people suffering less than before when they were given the treatment that left people with sequelae, but it still brings sadness.*

*P4: For those of us who have already lost family members due to this disease, when we learn that the disease has become easy to treat—that one can treat someone and they recover—I feel a sense of joy. Because if they are diagnosed, there is treatment and recovery.*

*P7: Sleeping sickness used to trouble us before because of its treatment, which was dangerous. Now that there is a good treatment, for my part, I feel joy because if someone is found to be sick, it is an opportunity to prevent the disease from spreading; we will treat them.*

**To what do you attribute the fate of sleeping sickness?**

*P2: Sleeping sickness is like any other disease that can infect humans.*

*P6: We learned about this disease in school; it is like all other diseases, like malaria when the mosquito bites you. It is the same with this disease: when that fly bites a sick person and then bites you too.*

**Does sleeping sickness cause fear when you hear about it?**

*P9: Yes, the disease causes fear because it kills more easily; the person who catches it can die.*

*P3: It frightens us in the sense that it makes people go mad.*

**Do you think you would go for screening at a health center/general referral hospital when you present signs suggestive of sleeping sickness?**

*P4: It is not easy, because due to the fear of this disease, the person may start asking themselves questions like, "Why does this happen to me?"*

*P8: I would accept, and I would go for examination. If the disease is confirmed, I would take the treatment. Moreover, nowadays, they give a little money for food; I too would like to experience that.*

**Why, in your opinion, are some people afraid to be screened for sleeping sickness?**

*P1: People are afraid of the history of the disease, the damage caused by this disease, the deaths, the sequelae.*

*P5: The fear comes from the history of the disease: if I am declared positive, will I live, or is it death? That is the reason many are afraid.*
