# Supplementary material for: Barriers to integration of passive screening for sleeping sickness in Bibanga Health District, Democratic Republic of the Congo
Source: PLoS Negl Trop Dis. 2026 Apr 8;20(4):e0014179. doi: 10.1371/journal.pntd.0014179 (PMC13089886; doi:10.1371/journal.pntd.0014179)
Supplement: S1 File — (ZIP) [file pntd.0014179.s001.zip › S1_Verbatim transcripts/1_AS_BUFUA/AUD.5_ENT_IT_BUFUA.docx]

**INTERVIEW WITH HEALTHCARE PROVIDERS OF THE BIBANGA HEALTH ZONE**

**Audio N°5: Interview with the Head Nurse of the Bufua Health Area**

**1. Knowledge of HAT Control Strategies:**

**Could you tell us about the strategies used by your Health Area to reduce the prevalence of sleeping sickness in the Bibanga Health Zone?**

*Thank you. Here, we have barriers that are used at different levels. Firstly, at the community level, tsetse fly traps are installed, which has reduced the flies that used to bite us. At the same time, there was also awareness-raising on bush clearing; the local youth were able to help reduce the number of flies that transmit the disease. Secondly, there is also screening carried out at two levels. First, here at the center, we perform screening using the algorithm. There is also the mobile team that passes through the Health Area from time to time to screen. At the same time, patients screened positive for HAT are treated. Treatment is also free, which helps reduce prevalence. For those who test CATT positive or RDT positive, we follow up to see if it remains positive or if it can be confirmed after some time. In short, that's what has helped reduce the disease.*

**Since you were assigned to this Health Area, have you ever diagnosed HAT in your center? If not, why? If yes, how do you do it?**

*Yes, we have already done so, and if I'm not mistaken, we have already had more than 3 cases. For the diagnosis, we received cases, we suspected trypanosomiasis, and we performed an RDT which came out positive. We referred the patient to the KATANDA CDTC where the disease was confirmed. They received treatment and were cured.*

**Have you ever encountered resistance when referring a patient for confirmation of a suspicion you made? If yes, what did you do to convince them to go to the HAT confirmation center?**

*Well, here we do not provide treatment. I mentioned that we refer to the CDTC Katanda. Afterwards, if the disease is confirmed, we receive feedback that such and such a patient is positive. We also then provide psychological support. You see, there are also patients who think it's a mystical disease, perhaps they think... That's not true. For those who have doubts, we provide psychological support and reassure them to take the treatment. We follow up, just to confirm that they have taken the treatment, but we do not have the treatment available in our facility.*

**2. Perception of HAT Integration**

**In your opinion, when we talk about integrating sleeping sickness control activities into Primary Health Care, what do you think of?**

*Integration was, firstly, a briefing. Providers first had to be briefed on screening, because we do the screening. After the briefing, we needed to be supplied with tests, then a reporting model. After that, we also receive supervisory visits to see how we perform the technique and whether we respect the algorithm, things like that. So what I expect from integration is simply the briefing, the performance of RDTs, and reporting.*

**Do you think integrating screening and diagnosis alone is sufficient to eliminate HAT in the Bibanga Health Zone, or are other complementary strategies needed? If not, why? If yes, which ones?**

*Yes, for me, integration also needs to include community involvement in monitoring trypanosomiasis because it is the community that lives with the disease. Often with a lot of awareness... So I meant to say that the RECOs themselves should also conduct community-based active case finding because currently, trypanosomiasis screening is only done at the facility level. Having community-based screening at the grassroots level could help eliminate trypanosomiasis.*

**In your opinion, was it necessary to implement these activities at this specific time? Why?**

*It was justified, because the disease was present and had caused challenges. When they implemented it, we saw that patients suffering from sleeping sickness were treated. That's important because we had cases. Sometimes, if they hadn't done it, the cases that were already infected could have contaminated others, and it could have turned into an epidemic. Now that this technique exists, it helps, and the community is calm; it's no longer a problem.*

**How are the HAT control activities implemented in the Minimum Activity Package of your Health Center beneficial for you and your facility?**

*Yes, it is beneficial, especially for us providers. It's beneficial for diagnostic confirmation. When you receive a patient, if you miss the diagnosis, if it's HAT and you think it's malaria and you treat for that, there will be no success. So it's beneficial for us, for confirming diagnoses and for proper patient management.*

**How are the control activities implemented in the Minimum Activity Package of your Health Center beneficial for the community in your Health Area?**

*Yes, it is beneficial. Moreover, if I remember correctly, it's a disease that has left a legacy among patients in the community. You see someone who was fine; if they are not treated in time, they develop behavioral disorders and become useless in the community. With that, you could end up with many useless people in the community who will always be beggars. But when there is treatment and care, someone takes an interest in what they have and engages in development; it is also a factor that promotes development.*

*Since this activity was integrated, we have screened patients who were confirmed and treated. That's already good. There are cases that were screened RDT positive; we follow them closely, and I think that in a while we can be done with the disease.*

**What do you think about the time you spend screening a suspected HAT case in your Health Center compared to daily routine activities? Does this time represent a loss of income for you and your facility?**

*It's our job. We are here for the health of the community; we help them. But we do have some difficulties. I would like to speak regarding the government. I am a state agent; if the government supports me, why should I complain? But when we complain, it's because the government does not support us well. But it's our daily job; we will always do it. It is not a loss of income.*

**3. Perception of HAT Elimination**

**In your opinion, what do you think of when we talk about eliminating HAT?**

*When we talk about eliminating HAT, it means, well, it's as the word suggests: it means the disappearance of the disease. We want the disease to disappear. Now, with this, we treat so that people can no longer become infected. It's just about prevention and treatment; that's what I think.*

**Do you think eliminating HAT is an urgent matter in the Bibanga Health Zone?**

*Yes, it is really urgent because it hinders the community. You see, if you arrive in a community where you find 50 people with this disease, with behavioral disorders, they no longer work, they are useless for anything. You see all that; it's a factor of underdevelopment.*

*You see what happened in 2020. Our Health Area borders a forest called Kapongo on that side, and all the people who come from that side are victims of this disease. With the flies also present, that's what led us to have cases in the year 2020.*

**In your opinion, what is the most effective way to eliminate HAT? Why is this method more effective than others?**

*In my opinion, there is one thing I really appreciated in the treatment of sleeping sickness. It's the per os (oral) treatment that the community accepts easily. When we compared the old product, which is well-known, ARSOBAL, you see, a sick person who receives that product is left with sequelae. In the community, if you are seen acting strangely, they say you act like someone who took ARSOBAL. But with the new oral treatment, it's good. However, I would also like to suggest that even if the disease could be eliminated, this treatment should be made available in our facilities for surveillance so that if there is a case, it can be managed just like we do with malaria management. Malaria management does not require going elsewhere; it's done on the spot. We find the disease and treat the person.*

*What do you do at your level to make HAT elimination a reality by the 2030 horizon?*

*At our level, things have already been done, but I would like to insist on the community. You see that screening is voluntary. We need to explain to the community that they must undergo voluntary screening after a certain time, and also explain the signs of the disease: if someone shows such a sign, come directly for screening. With that, it can work. Moreover, 2030 is far; I could say even by 2023.*

*I will talk about community involvement because with community involvement, many things go very well.*

**4. Community Accessibility to HAT Screening Services**

**What do you think about the attendance rate at the Health Center by the community? (Guide towards possible obstacles)**

*At our level, the community does attend our center, but we can talk about the community's difficulties. A person may be sick but lacks the means to come for care. Apart from financial means, there are also shortages of essential medicines, such as we experienced in recent years. These are difficulties, not barriers, because we do have patients, as you can observe. Notice that we have patients. In our area, the entire population lives within 2 km of the Health Center, so distance does not prevent patients from attending the center. At 00h? Accessibility is easy, especially since it's the town; people are right here.*

**What do you think about the availability of HAT screening in your Health Center? (Guide towards obstacles)**

*Currently, it's okay, but if I recall, in the two previous years, we experienced shortages of HAT RDTs for more than six months. It was many months, but the problem wasn't at our level; it was at the supply level. I don't know what blocked it for that to happen. It's true that this could have constituted a barrier to integration, but at our level, we continued to refer suspected cases to the CDTC even without RDTs.*

**How do patients perceive a positive HAT RDT result when they came for consultation suspecting malaria?**

*The population is already used to it; even when you talk to them about it, it doesn't bother them much. I don't really see patients who panic. But I know the case of a former patient who exclaimed, "Why must I suffer from this again, especially since I haven't traveled much lately?"*

**What do you do in a situation where a person refuses to believe the result of a positive HAT RDT after examination?**

*There are two methods: We provide explanations with examples. The second, if they do not get confirmation quickly and do not get treated in time if they have the disease, we explain the consequences to them.*

**How do you judge the acceptability of going to a referral facility for confirmatory diagnosis by an RDT-positive suspect?**

*For people who have already experienced the disease in their family and have seen the consequences, they do not hesitate as soon as you tell them to go to the confirmation center. But for someone who has only heard about it, they find it hard to believe, they go back into the community and might even try to run away. For those, we even send the RECOs to raise awareness and bring them back to the center.*

**What prevents the community in this Health Area from accessing the care offered by your Health Center?**

*I would say that the barriers we can find in our Health Area are, firstly, the ownership of the activity by the community itself. Because the community is the victim; they need to take ownership of the activity of getting screened voluntarily. For us providers, we need to constantly multiply awareness-raising activities on the signs of trypanosomiasis and also on getting screened regularly. Since we have the CDTC located right in the town of Katanda, if there are signs, we can refer them; there's no problem. It's just the ownership of activities by the community that remains... because there are communities that accept, and others think maybe it's demonic. We need to explain to them. That's the issue at that level.*

*Ownership means first accepting that the disease exists, accepting screening as well. Because there are some communities; the disease exists, but they think it's demonic; perhaps they need to go pray. But while they are going for intercession, the fly can bite them and they can infect others. It's about... understanding the disease and getting screened at our level is good, but for us providers, we are available 24/7; as soon as there is a case, we can screen and manage.*

*Another thing, you should know that in the Bufua Health Area, there are more than ten traditional practitioners. That is also a... one of the obstacles, because they are not briefed on the signs of trypanosomiasis. They should also be briefed because, just like us, they also receive patients. They need to be aware of the signs of trypanosomiasis; they also need to be able to refer a case they think might have the disease. This is a difficulty at our level that needs to be addressed.*

*There are also barriers that cannot be overlooked, which is the poverty of the population. You see, we are a healthcare facility, and in the healthcare facility system, when a patient comes, they first have to pay for the consultation form, you see. A patient may have the disease, but the consultation form is payable. They arrive and don't have the money to pay for the form; that's a barrier, you see. The lack of means means they might go back home. The time they spend trying to get the money to be consulted first... That's one of the barriers we also have in our Health Area. You see, a patient who lacks means and stays at home is a barrier, because someone should be able to get screened directly without spending anything, since screening is free.*

**What do you suggest to improve the utilization of the Health Center by the communities?**

*Former patients who were victims can play a role in sensitizing other new cases. In addition to that, community involvement needs to be supported with technical support, including essential medicines.*

*If the center is supported with medicines and also if the price is reduced, so that the price is accessible to the community. If the price is really much cheaper, that would be good.*

**Thank you.**
