# Supplementary material for: Barriers to integration of passive screening for sleeping sickness in Bibanga Health District, Democratic Republic of the Congo
Source: PLoS Negl Trop Dis. 2026 Apr 8;20(4):e0014179. doi: 10.1371/journal.pntd.0014179 (PMC13089886; doi:10.1371/journal.pntd.0014179)
Supplement: S1 File — (ZIP) [file pntd.0014179.s001.zip › S1_Verbatim transcripts/1_AS_BUFUA/AUD.6_ENT_AC_THA_BUFUA.docx]

**INTERVIEW WITH FORMER HAT PATIENTS IN THE BIBANGA HEALTH ZONE.**

**Audio N°6: Interview with a former patient from the Bufua Health Area**

**I. Perception of Sleeping Sickness and Screening:**

**What is your opinion on the existence of sleeping sickness in your community?**

*Yes, it exists here in KATANDA.*

**In your view, what is the origin of sleeping sickness?**

*When we go to our fields wearing a black shirt, this insect can bite you randomly because of the color that attracts it. Or, if you pass through a bushy area, that's where the flies are hidden and they bite people.*

**Is sleeping sickness dangerous for those who contract it?**

*Sleeping sickness can be dangerous for those who do not follow the prohibitions after their treatment. For example, drinking alcohol and smoking. If the person does not respect these prohibitions, they have compromised their treatment, and there will be consequences. The danger is that the person can start to lose consciousness or develop behavioral disorders, as I have seen in others. I have also seen limb tremors.*

**Was it easy to suspect sleeping sickness, as one might do for other diseases like malaria, which you might suspect even without consulting healthcare services at the health center or general referral hospital?**

*For sleeping sickness, it is not easy. You always have to go to the health center or the hospital for tests to confirm it.
The signs are prolonged sleep and fatigue. Apart from that, I do not know other signs.*

**What motivations prompted you to get screened for HAT at a health facility?**

*What prompted me was the malaise I was experiencing. I always felt very tired, and since I work in distant fields, I was told it was due to the labor. But one time I had a high fever, and when I went to the health center after examinations, they gave me a transfer note for the sleeping sickness diagnostic center.*

**Could you describe the pathway you took from when you first felt ill until the moment sleeping sickness was diagnosed? Approximately how long did it take before being diagnosed?**

*I am a RECO [Community Health Relay?] in KATANDA 2. What we have always been told is that when you feel sick, if you take treatment for two days and there is no change, you must come to the health center for examinations. So, for me, since I wasn't feeling well, I took pills on the first day and then the second day without any change, I went to the health center. I had the illness for about a month, but the pathway to diagnosis took about a week or a week and a half.*

**What was your feeling when you were told you had sleeping sickness?**

*For me, I had no particular feeling because it is a disease like any other.*

**In your opinion, why are some people afraid to get screened for sleeping sickness?**

*Previously, people were afraid of the lumbar puncture and the number of days of hospitalization. But the fear is also when you are told that there are microbes in the blood; others think they are going to die, especially when it is announced to them in public. However, regarding sleeping sickness, it is said that it is like all other illnesses.*

**II. Perception of Health Services**

**When you feel sick, where do you go first to seek a solution? (Church, traditional healer, or modern medicine?)**

*When I do not feel well, I start by going to the pharmacy. I buy the products myself. After two days of taking them, if there is no change, I go to the health center for examinations.*

**Where were you screened and treated for sleeping sickness?**

*I was at our center, and then they told me that my blood was not good; I needed to go to the sleeping sickness center. When I arrived, the FEMETRO [organization, likely involved in disease control] people took my blood again. It was then that they said I had sleeping sickness, and that is where I received my treatment.*

**Before being diagnosed and treated for sleeping sickness, where did you go first for care? (Elaborate: why did you leave that place for another?)**

*When I started the treatment, I thought it was just a fever. But when I noticed after two days that there was no change, I went to the health center. For now, I still feel better, apart from this flu that is bothering me.*

**How do you appreciate the services offered by the health center you attend in the village?**

*The service at our health center here is truly appreciable. They receive us very well. Even if you do not have money, they receive you first, treat you, and then you can go find the money to pay afterward.*

**How do you appreciate the distance traveled to reach the health center?**

*No, the distance is not too long, and even if it is long, for a sick person, it does not matter.*

**How do you appreciate the waiting time to be received by the health center staff?**

*For me, it does not pose a problem. Since they know I am a teacher, if I come, they let me go ahead, and I receive treatment because I have to go and teach.*

**How do you appreciate the availability of the health center nurse when you need them?**

*Availability is good here. Whether you come during the day or in the middle of the night, there is always someone here.*

**How do you appreciate the cost of consultation and care at the health center?**

*It is a fair price; it does not prevent us from paying.*

**Are you aware that screening examinations and the management of sleeping sickness are free wherever you may be?**

*Yes, I know. I myself am a former patient; I know all about it.*

**Is there a problem that sometimes prevents you (or the members of your household) from attending the health center for care?**

*For me, I find no barrier that prevents me from attending the health center. As for money, we are in a farming area, and we know how to raise livestock. So even when there is no cash, I know I can sell even a rooster to solve this problem.*

**What are your suggestions for improving access to healthcare services in our Health Area/Health Zone?**

*I wish for us to be built a good building for the health center and for our neighborhood chiefs to raise awareness effectively within the community so that everyone knows that when they are sick, the health center is where they should go.*

**COMMENT**
Sleeping sickness is like any other illness; people should not be afraid to get screened.
