# Supplementary material for: Barriers to integration of passive screening for sleeping sickness in Bibanga Health District, Democratic Republic of the Congo
Source: PLoS Negl Trop Dis. 2026 Apr 8;20(4):e0014179. doi: 10.1371/journal.pntd.0014179 (PMC13089886; doi:10.1371/journal.pntd.0014179)
Supplement: S1 File — (ZIP) [file pntd.0014179.s001.zip › S1_Verbatim transcripts/1_AS_BUFUA/AUD.7_FG_FILLES_BUFUA.docx]

**FG WITH MEMBERS OF THE BIBANGA HEALTH ZONE COMMUNITY**

**Audio No. 7: FGD with Girls from the Bufua Health Area**

**I. Knowledge of Sleeping Sickness**

**Do you know a disease that makes the person who has it fall asleep at any time and uncontrollably? What do you call it in your language? What are the different names for this disease and what do they mean?**

*P6: Sleeping sickness;
P3: We don't know any other terms besides that one;
P8: Yes, if someone has sleeping sickness, we say they have the '9:6' disease because their head doesn't function properly; they have a disorder;
P7: The somnolence disease because the person dozes off at any time…*

**Apart from the fact that the person experiences uncontrollable sleep at times, do you know any other signs attributed to this disease?**

*P1: As signs, we can see a high fever, headaches, and also memory loss;
P4: We can also see a person who sleeps at any time, even while working, as well as behavioral disorders as if they were crazy.*

**Where does this disease come from and how is it transmitted to humans?**

*P2: It comes from flies and mosquitoes if we don't use a mosquito net;
P6: Also very small insects;
P8: There are also forest insects and mosquitoes that bite people….*

**Are there ways to protect oneself from sleeping sickness?**

*P6: (....) we do not know the mode of transmission;
P5: Sometimes we think it's when mosquitoes bite, they leave microbes in the body….*

**II. Perception of Health Services**

**What do you do here in the village when you feel sick? (Where do you go to find a solution?)**

*P2: To find a solution, we go to the Health Center or the hospital;
P1: It's at the Health Center that we find a solution;
P7: Also to the hospital so we can have tests done;
P8: Sometimes we look for a solution elsewhere before going to the Health Center. For example, with a cough, there are fruits like lemon and others that treat it well. If there is no change, then we go to the Health Center;
P6: (.....) like with a cough, sometimes you can take modern medicine without success, but when you take lemon, you feel a change. If there is still no change, you need to go to the Health Center for tests because that's where you can find out;
P10: We can buy a product from the pharmacy and take it. It's when there is no change that I go to the Health Center.*

**When you think, based on the signs mentioned (reiterate some signs mentioned by the group), that a person has sleeping sickness, what do you do to find a solution?**

*P4: We will accompany them to the Health Center for tests to identify the disease;
P9: We will bring them to the Health Center for tests;
P6: Since it's sleeping sickness, we will take a CSF (cerebrospinal fluid) sample because that's where we see the microbes;
P5: Why go elsewhere, like to church? Because at church they don't treat; they pray there. But you can pray and there is no change, which is why you always need to go to the hospital;
P9: When we take the patient to church, they don't even know the disease. They will pray, but they won't tell us what disease they are suffering from*. What will we do?

**Do you know the structures that organize or carry out screening for this disease? If yes, which ones**?

*P1: Yes, at KATANDA 2;
P3: At the secondary hospital which is on the main road;
P7: At the referral hospital;
P6: At the BIBANGA referral hospital…*

**How do you appreciate the services offered by the Health Center you frequent in the village?**

*P2: People are different. Sometimes you find a nurse who sulks at people; he looks at you as if you have no value in his eyes. But there are others who are very welcoming; as soon as you arrive, they start asking questions to find the causes of the illness;
P5: When I accompanied my friend, we were well welcomed and received. Since she couldn't speak, I answered the questions, and it went well;
P1: Our Health Center here is wonderful. When we are sick, even if we don't have money, we are received and given treatment. Afterwards, we go and find the money to pay…*

**How do you appreciate the distance to travel to reach the Health Center?**

*P10: The distance is acceptable for everyone, even for those who live down below;
P6: There is no long distance for those who are in BUFUA. At least for those in other neighborhoods, if they are not satisfied with the treatment they receive at home and they want to come here, then it becomes far…*

**How do you appreciate the waiting time before being received by the Health Center staff?**

*P8: It's quick; the waiting time is short;
P2: It goes well and quickly;
P1: It's also quick for me…*

**How do you appreciate the treatment you receive at the Health Center?**

*P9: Yes, we are treated well and are always satisfied;
P6: Yes, it's as she just said…*

**How do you appreciate the availability of the Health Center nurse when you need them?**

*P10: The availability is good;
P5: Sometimes they can step away for a short time to wash or eat, but when you call them, they come quickly;
P3: For me, they didn't come on time;
P2: I have noticed that the men come quickly, but the women are always slow. Sometimes we call her, she doesn't come, and it's the man who comes quickly in place of the one who should be there.*

**How do you appreciate the cost of consultation and care at the Health Center?**

*P7: The cost is affordable and low, but people are bad; they don't pay all the fees and always leave without paying everything;
P6: She is right. For example, I was supposed to pay 6500 francs. I had given 3000 francs, and the rest remains unpaid to this day;
P4: The cost is good…*

**Are you aware that tests for screening sleeping sickness are free?**

*P9: We know. Even when the FEMETRO team comes, they examine us without payment. Even if they find that you have the disease, they take you to the Health Center; you don't pay there. All the charges, even for food, are free…*

**Is there a problem that prevents the community from frequenting the Health Center for care?**

*P6: For sleeping sickness, people are afraid of the lumbar puncture. Especially if they puncture you and then don't find the disease, you are left with pain;
P3: For other illnesses, it's due to a lack of money. When we don't have the means, we don't go for care. We buy the product at the pharmacy and take it. That's why we stay home;
P2: For other people, it's negligence; they simply refuse to go for the FEMETRO check-up, even when the team is there…*

**What are your suggestions for improving access to healthcare services in our Health Area/Health Zone?**

*P9: (....) look at our center. If they build a good building for us, that would be good because this one already has cracks;
P10: In our center, we don't even have products. When you come at 10 PM, they will give you a prescription to buy products. Which pharmacy will you find open at that hour? We want our pharmacy to be stocked with products. Even if we are going to buy them, we want it to be here and not elsewhere;
P7: Our center has no beds, and all the mattresses we have are full of bedbugs. We want them to be replaced…*

**III. Perception of Sleeping Sickness and Screening**

**How do you feel within the community if you are told that a certain person has tested positive for sleeping sickness after examinations?**

*P2: We feel very bad. Because if it's our friend, we ask ourselves questions: how are we with her every day, where could she have gotten this?
P5: We all have the same answer; we feel bad because of what happened to our friend;
P7: It hurts a lot to learn that a loved one is sick; it's the same with sleeping sickness.*

**To what do you attribute the fate of sleeping sickness?**

*P4: There is what comes from a bad spell. You will see the person being treated, but they do not heal. Eventually, they go mad or start losing consciousness. But normal illness also exists; that's the one where, when treated, the person heals;*

**Does sleeping sickness scare you when you hear about it?**

*P8: Sleeping sickness is scary; it's a contagious disease. I'm afraid of being contaminated too. It's a rare disease in the population, but it is too dangerous;
P10: It's scary. If someone has this disease, you have to be careful, even when you visit them. You must not get too close to them; we fear death;
P6: It's scary, but I cannot run away or abandon my friend. If my friend has it, I will go visit her. If we have to die, we will die;*

**Do you think you would go for screening at a Health Center/General Referral Hospital if you presented with signs suggestive of sleeping sickness?**

*P1: I cannot because I am not convinced that I have it;
P3: I will accept because one needs to be diagnosed to confirm or refute what the nurse said;
P9: I also agree to be examined for sleeping sickness. When I come to the Health Center, it's because I am sick; I need to be cured. The disease needs to be discovered, and I be treated;*

**Why, according to you, are some people afraid to get screened for sleeping sickness?**

*P10: It's a serious disease, that's why it's scary;
P6: Many people are afraid of the injection, especially the lumbar puncture;
P8: People are afraid because the product given is toxic; it makes your head spin, and you risk becoming '9:6' (mentally impaired);
P2: This product is very toxic. When you start treatment, you will be forbidden to shout. If you shout, the product reacts, and you risk losing consciousness;
P4: Sleeping sickness exists. The normal form heals with modern medicine. The one that comes from bad spells does not heal; it progresses until it makes the person crazy.*
