# Supplementary material for: Barriers to integration of passive screening for sleeping sickness in Bibanga Health District, Democratic Republic of the Congo
Source: PLoS Negl Trop Dis. 2026 Apr 8;20(4):e0014179. doi: 10.1371/journal.pntd.0014179 (PMC13089886; doi:10.1371/journal.pntd.0014179)
Supplement: S2 File — (ZIP) [file pntd.0014179.s002.zip › S2_Verbatim transcripts/2_AS_KATANDA1/AUD.10_FG_FILLES_KATANDA1.docx]

**FG WITH MEMBERS OF THE COMMUNITY OF THE BIBANGA HEALTH ZONE**

**Audio No. 10: FGD with Girls from the Katanda 1 Health Area**

**I. Knowledge of Sleeping Sickness**

**Do you know a disease that makes the person who catches it sleep at any time and uncontrollably? What do you call it in your language? What are the different names of this disease and what do they mean?**

*P5: Sleeping sickness*

*P1: Drowsiness sickness;*

*P7: We don't know another name, it's only those two.*

*Apart from the fact that the person at times has uncontrollable sleep, do you know other signs attributed to this disease?*

*P8: The person makes too much noise, they talk to themselves;*

*P4: The person sleeps too much during the day and suffers from insomnia at night;*

*P3: The person starts to talk a lot;*

*P5: The person complains too much about headaches…*

**Where does this disease come from and how is it transmitted to humans?**

*P7: Sleeping sickness comes from the bite of the fly or the mosquito; these flies are especially found in the bush where there is a lot of undergrowth; they are not found here in the village; if it bites you, we notice the blood; it is by these signs that we recognize it;*

*P6: It's as she said, it comes from flies and mosquitoes. You see how we have mango trees; that's where flies and mosquitoes hide, and when you come to rest under it, the fly can bite you and give you the disease;*

*P10: Transmission is when I have the disease, the fly comes and bites me and then bites another person; they also catch this disease…*

**Are there ways to protect oneself from sleeping sickness?**

*P6: By clearing the plot; if there are bushy areas, you have to cut them;*

*P5: Since we girls really like the river, we have to do communal work where we are active so that the place is clean and clear, far from the bush;*

*P1: Avoiding flies in the house;*

*P9: Maintaining cleanliness in the house;*

*P4: After the rain, we also see mosquitoes in puddles; you have to empty and dry out this water because it creates flies and mosquitoes; afterwards, they come to bite us and cause the disease.*

**II. Perception of Health Services**

**What do you do here in the village when you feel sick? (Where do you go to find a solution?)**

*P5: When a person falls sick, on the first day we start by giving them medications; if they do not recover, we can take them to the nearest health zone or to a dispensary to be examined or treated; first, we do the tests and when we find the disease, we start treatment and the vaccine;*

*P8: If someone starts getting sick, their mother can also take them to the church where they are used to praying; we can pray and sometimes there is healing;*

*P9: For others as well, if the sickness starts, we can go to the pharmacy and buy medications; but if there is no change, we take the sick person to the church and there too if there is no change, afterwards we must still take them to the hospital to be treated.*

**When you think, based on the signs mentioned (reiterate some signs cited by the group), that a person has sleeping sickness, what do you do to find a solution?**

*P3: We must take them to the hospital so they can be examined;*

*P8: Yes, it's always to the hospital so they can examine them;*

*P10: When we see a person with these signs of sleep, we take them to the sleeping sickness hospital so they can be examined there;*

*P9: There is also a person who received medication for sleeping sickness; there was no change, we even see them go mad; what else can we do, do we leave them?*

**Do you know the structures that organize or carry out screening for this disease? If so, which ones?**

*P4: Yes, we take them to the sleeping sickness hospital;*

*P6: I know at the secondary hospital and at Bibanga;*

*P7: Yes, it's always there;*

*How do you assess the services offered by the health center you frequent in the village?*

*P1: As for our hospital here, it is not pleasant to come at night with a sick person because they don't even have medication if treatment needs to start; they just give you a prescription and that night you have to knock on the doors of pharmacies in the market to buy the product;*

*P6: Another person can bring a sick child; instead of starting treatment, they leave the child and give the prescription to the one who brought them so they can go get the products, and they do not attend to the child;*

*P9: In this center, they like to write. You'll see, they write here, they write there; there is never a moment when we bring a sick person, treatment starts, and then something else afterwards; it is always writing, at the risk of losing someone's life;*

*P10: Another person may have a case of illness and even prefer to go to Bibanga instead of coming to spend their time here.*

**How do you assess the distance to travel to reach the health center?**

*P6: The distance is not a problem because the time you will spend here can even exceed the time it takes to get to Bibanga;*

*P5: We come from different corners, but the distance to reach the center is not long.*

**How do you assess the waiting time before being received by the health center staff?**

*P9: The waiting time for the center here at our place is enormous and tiring; you can come with a sick person, you are there but no one looks at you, and if they want to look at you, they see you as if you were a rotten thing. You are there as if there is no one; they sit at their table and start writing; you arrived at 11:00 AM, they receive you at 5:00 PM;*

*P8: Consider also the case of a person who is not from the area and comes from the interior; their means do not allow them to pay for care, because everything they find is expensive. The money is not enough for everything they ask for, and they have no more means; they have to return from where they came to get more money, whereas in Bibanga, even with the little you have, if you pay a deposit, they will first take care of you and after treatment, they keep you so you can pay the full amount.*

**How do you assess the treatment you receive at the health center?**

*P3: Sometimes you arrive, they treat you well, and you go home happy, but other times what they do is not pleasing at all and you go home worried;*

*P5: Another difficulty we encounter is when you come with an appendicitis problem; they just send you to Bibanga; their job is just to wait for you to come back so they can operate;*

*P6: Another case: you might see, for example, someone with tuberculosis; they are asked to pay a certain amount of money and given an appointment; afterwards, when they return, they are told we do not have the tools for these tests.*

**How do you assess the availability of the nurse at the health center when you need them?**

*P10: There are also times when the caregiver is not on site; we have to call them by phone so they can come; sometimes they come quickly, sometimes they take their time;*

*P9: Other times you can come to the center at 11:00 PM and you find no one; you leave the sick person to look for the caregiver who only shows up at 4:00 AM;*

*P2: Like a center that is next to my home, the caregivers have divided the days; when you arrive at the center with a sick person, you have to go get the caregiver, and if we call the one who is not on duty that day, it is really a problem for them to come see the case, and during this time your sick person becomes even weaker;*

*P1: I would also like to add this: there are centers where they have divided the hours. You come for a consultation; the one you find tells you that they have already finished, but the one who is supposed to come arrives very late, and during this time you are there waiting for the caregiver to arrive to be treated.*

**How do you assess the cost of consultation and care at the health center?**

*P4: The consultation form is not expensive; it's 500 francs, but if we start with tests, each test has a price; the rapid diagnostic test you pay for, the stool test has a price, the thing to put under the armpit you also pay for; now, if your case requires a transfusion, there is a separate price; for treatment as well, there is a separate price;*

*P2: The cost is not excessive, because if you go there to the hospital for surgery, you will pay a lot; you even pay for the bed you sleep on; here they ask for little;*

*P8: Sometimes someone might agree to pay even this money, but they do not have it on hand; if they ask to start treatment and they will come to pay, they are refused. They only want them to get the full amount before treatment, even though they are exhausted.*

**Are you aware that tests for screening sleeping sickness are free?**

*P6: We know that everything is free; they even give food for free;*

**Is there a problem that prevents the community from attending the health center for care?**

*P7: Yes, due to lack of means; for example, I am sick but I lack money; I will stay at home;*

**What are your suggestions if we are to improve access to health care services in our health area/zone?**

*P2: We need a pharmacy with products so that if a person is sick, instead of giving them a prescription, we can treat them directly and promptly;*

*P10: Firstly, I ask that caregivers have love for the sick people who come to the center;*

*P9: That caregivers improve the reception at the center.*

**III. Perception of Sleeping Sickness and Screening**

**How do you feel in the community if you are told that a certain person has tested positive for sleeping sickness after examinations?**

*P8: It hurts and worries us, because we fear being contaminated;*

*P3: We keep our distance from the sick person;*

*P9: It scares us because sleeping sickness is like COVID; it's contagious;*

*P6: It hurts because someone close to me has a bad disease;*

*P5: It hurts us because there is a risk that we too may be contaminated;*

*To what do you attribute the fate of sleeping sickness?*

*P3: It is especially a curse from wicked people;*

*P4: It can also be witchcraft;*

*P7: For others, it is also caused by family problems;*

**Does sleeping sickness scare you when you hear about it?**

*P10: It scares me because it is very contagious;*

*P6: It scares me because we have cultivated many things in our fields, and like the mosquitoes that hide there, the mosquitoes that bit them can bite us too and transmit the disease to us;*

*P9: It scares me because I have never seen a person with this disease, and when I am told it is that, it will scare me to be with them;*

**Do you think you would go for screening at a health center/general reference hospital if you are presented with signs suggestive of sleeping sickness?**

*P1: I must accept because I need to be treated;*

*P2: I do not accept because the person telling me has not confirmed it; they are not sure;*

*P5: For me, I would go to confirm; after examination, if it's not that, they will tell me, and if it is the disease, I accept so they can treat me;*

**Why, according to you, are some people afraid to get screened for sleeping sickness?**

*P2: They are right; someone who does not have it, when they are told to go for an examination, they can arrive at the center and come into contact with those who have it; you see, they also become sick;*

*P6: They are afraid because it is a bad disease; if you are told you have that, you see how shameful it is;*

*P8: If you are told you have that, everyone will start to avoid you.*

**Thank you.**
