# Supplementary material for: Barriers to integration of passive screening for sleeping sickness in Bibanga Health District, Democratic Republic of the Congo
Source: PLoS Negl Trop Dis. 2026 Apr 8;20(4):e0014179. doi: 10.1371/journal.pntd.0014179 (PMC13089886; doi:10.1371/journal.pntd.0014179)
Supplement: S2 File — (ZIP) [file pntd.0014179.s002.zip › S2_Verbatim transcripts/2_AS_KATANDA1/AUD.11_FG_GARCONS_KATANDA1.docx]

**FG WITH MEMBERS OF THE COMMUNITY OF THE BIBANGA HEALTH ZONE**

**Audio N°11: FGD with boys from the Katanda 1 Health Area**

**I. Knowledge of Sleeping Sickness**

**Do you know a disease that makes the person who catches it sleep at any time and uncontrollably? What do you call it in your language? What are the different names for this disease and what do they mean?**

*P1: We call it sleeping sickness;*

*P4: (....) we call it maladie du sommeil in French;*

*P9: We call it sleeping sickness because it makes people sleep;*

**Apart from the fact that the person has uncontrollable sleep at times, do you know of any other signs attributed to this disease?**

*P10: (....) body weakness;*

*P7: (....) loss of consciousness;*

*P6: Sleeping a lot;*

*P4: The way of doing things, the person becomes abnormal; if you tell them to do one thing, they do another;*

*P3: The presence of lymph nodes on the body;*

**Where does this disease come from and how is it transmitted to humans?**

*P2: It comes from unsanitary conditions, from overgrown vegetation;*

*P3: It comes from flies, when the fly bites you;*

*P7: Transmission occurs through blood contact using a razor blade that has injured a person during haircutting, and if that same blade is used to cut another person's hair;*

*P8: When the fly bites a sick person and then bites another healthy person to transmit the disease;*

**Are there ways to protect oneself from sleeping sickness?**

*P1: Maintain cleanliness in our surroundings and clear the vegetation;*

*P2: (....) when we clear the bushes, we make sure that this fly no longer comes to bite us;*

**II. Perception of Health Services**

**What do you do here in the village when you feel sick? (Where do you go to find a solution?)**

*P9: We take him to the health center;*

*P8: We take him to the hospital;*

*P6: (....) we take him to the hospital, as has just been said;*

**When you suspect, based on the signs mentioned (reiterate some signs cited by the group), that a person has sleeping sickness, what do you do to find a solution?**

*P4: We take him to the health center for examinations to find out what illness it is;*

**Do you know the structures that organize or carry out screening for this disease? If so, which ones?**

*P8: At the sleeping sickness center;*

*P10: One can also take someone to the FEMETRO team when they pass through the neighborhoods;*

**How do you assess the services offered by the health center you frequent in the village?**

*P5: Our health center does not receive the sick; instead, they receive money. If you don't have money, they will watch you die;*

*P6: They don't care about people; they care about money. If they cared about people, they would try to provide a payment deferral for a patient's care; you would see how the patient would die in their presence;*

**How do you assess the distance to travel to reach the health center?**

*P9: The distance is good for everyone;*

*P7: No, for example, the people of Bakwa MASELA, they are far away and there is no center there; it even requires a motorcycle to take a patient;*

**How do you assess the waiting time before being received by the health center staff?**

*P3: They waste our time with bureaucracy; someone is sick, and they first send you over there for the file, which takes a lot of time, and meanwhile the illness gets worse;*

*P6: What my brother just said, I find that's exactly it; they delay the patient, especially to ask for money, and if there is no money, they don't pay attention to you;*

*P4: I support what the others have said because if you come with a case of a child needing a transfusion, they will make you go through every door without having anything ready, even risking losing the child in your hands; there is no quick reception here;*

**How do you assess the treatment you receive at the health center?**

*P10: Before, we had good treatment, but not now, why? Because things have changed. You come, they ask you for money for care, then they give you a prescription, so you do everything; you buy the products, they ask you for this, you do it; in the end, you even pay a fine of 50,000 francs. You look to find out the reason for these 50,000 francs, and you cannot. Is it just for an injection that they ask for all this money?*

*P4: We are treated well; we are cured of our illnesses, but it is not with their products. You come sick, they prescribe the prescription, as my brother just said, you go buy the products, they treat you, and you get better. It is with your own products, but before it was not like this; we had not known this: go pay for the products, the IV at the pharmacy; all of that was included in the invoice. They ask you for a lump sum to pay, and everything is included; you only start following the treatment;*

*P8: I want to add. You can come with a patient; they ask you for 5,000 francs; you think that these 5,000 are for all the care. Afterwards, they give you a prescription; you get to the pharmacy, and 50,000 francs remain there. That is why we avoid going there and prefer to get treated in BIBANGA;*

**How do you assess the availability of the health center nurse when you need them?**

*P4: Sometimes we find them, but there are days when you come with a patient and you are forced to leave the patient to look for the caregiver;*

*P2: For minor cases, we find the caregiver, but for a case like a transfusion, to find the lab technician, that's when you will lose the child;*

*P6: It's like we just said; you come with your patient, and you start spending your airtime to call the technician, and when he comes, he also starts giving you a prescription to go find the blood bag at the market. Sometimes you find that the pharmacies are closed; what will happen? Won't the patient die? All of this bothers us and puts us in difficulty;*

**How do you assess the cost of consultation and care at the health center?**

*P5: They don't ask for a lot of money; the file costs 500 francs;*

*P1: I say it is a lot because, besides the file fee, they don't know that when we come to the health center we are suffering and unable. They should treat us first without asking for anything. It's what they ask us for on top of the file fee that is a lot;*

*P3: I would like to add this: when someone comes to the health center, they have already accepted everything, even if it means paying 100 dollars. But what is important is to receive the patient and start the treatment; the rest can be arranged later. But for them, it's not like that; if you don't give, they tell you that they are not losing anything;*

**Are you aware that tests for sleeping sickness screening are free?**

*P8: We know, we are aware;*

*Is there a problem that prevents the community from frequenting the health center for care?*

*P4: The person may be in financial difficulty; they have no means to come for care or to the hospital;*

*P9: Only because of money;*

*P3: Because of money;*

*P7: Yes, there is also a problem in our health centers, the bad language of our nurses; that makes other people, instead of coming to the health center, go to the small clinics in our neighborhoods because there they are nice to the patients;*

**What are your suggestions for improving access to health care services in our Health District?**

*P10: (...) for sleeping sickness, the suggestion is that nurses go through the neighborhoods to examine household by household to discover those who have it and those who do not. Those who have it, we take them to the hospital for treatment;*

*P9: I ask that when nurses receive a patient, if they ask for 25,000 francs, that it should cover all the care, that there should no longer be the problem of a prescription to go buy products at the market;*

**III. Perception of Sleeping Sickness and Screening**

**How do you feel within the community if you are told that a certain person has tested positive for sleeping sickness after examinations?**

*P8: It hurts us very much because this disease makes one's head spin; when we learn that such a friend has this disease, it worries us; we wonder, how did he catch this disease? I am worried because everything we used to share is over; he will no longer understand me;*

*P5: It hurts us because it is a contagious disease; just as my friend caught it, that's how another can also catch it, and so on, and it spreads among all the young people in the neighborhood; that is why we are worried and we ask ourselves the question of how this disease arrived in this neighborhood;*

**To what do you attribute the fate of sleeping sickness?**

*P4: In fact, even when we ask ourselves the question of where it comes from, it is because it is beyond us; we don't really know the cause;*

*P1: I can say that it is a disease like Coronavirus, which was in Mbuji-Mayi; a person leaves here and arrives in Mbuji-Mayi, where there are many people coming from different corners; when they brush shoulders with someone who has it, they catch it. When they come back here, now I am not aware; we brush shoulders, and it catches me too. It is not witchcraft because I too can brush shoulders with another person and transmit it to them;*

**Does sleeping sickness scare you when you hear about it?**

*P7: It is scary because it makes you go crazy;*

*P6: It is scary because we see those who have it; we see how they behave;*

*Do you think you would go to get screened at a health center/general referral hospital if you are presented with signs suggestive of sleeping sickness?*

*P9: I agree to get tested because running away would cause my own death;*

*P8: I would like to say what my brother just said;*

**Why, according to you, are some people afraid to get screened for sleeping sickness?**

*P3: Whoever refuses is bewitching themselves; they need to find out if I have this or not;*

*P7: Everyone who runs away, it is just the fear of the injection;*

*P4: Others run away because they are ashamed of this disease; they think that if the presence of the disease is confirmed, everyone in the neighborhood will mock them;*

*P9: Another might run away because they have this disease; you know well that this disease makes one's head spin; at that moment, whatever you tell them, they will not accept it easily.*

**Thank you.**
