# Supplementary material for: Barriers to integration of passive screening for sleeping sickness in Bibanga Health District, Democratic Republic of the Congo
Source: PLoS Negl Trop Dis. 2026 Apr 8;20(4):e0014179. doi: 10.1371/journal.pntd.0014179 (PMC13089886; doi:10.1371/journal.pntd.0014179)
Supplement: S2 File — (ZIP) [file pntd.0014179.s002.zip › S2_Verbatim transcripts/2_AS_KATANDA1/AUD.12_ENT_PRESICODESA_KATANDA1.docx]

**INTERVIEW WITH OPINION LEADERS OF KATANDA 1.**

**Audio N°12: Interview with PRESICODESA**

**I. Knowledge of Sleeping Sickness:**

**What is your opinion on the existence of sleeping sickness in your community?**

*When we talk about sleeping sickness, we see a person who sleeps, who sleeps all the time. It is a disease that exists here in KATANDA with proof; if we go to the sleeping sickness treatment center, we will not fail to find at least one patient, which proves the disease is still present.*

**What are the signs that make you suspect sleeping sickness?**

*Yes, signs such as severe headaches, memory loss leading to madness, and then we suspect sleeping sickness.*

**Is there a treatment for sleeping sickness? (Is the disease curable?)**

*Yes, there is treatment, and the disease is curable. If you bring a patient, they will be cured very well as if there was no problem.*

**Is your Health Center capable of screening for sleeping sickness?**

*Regarding our Katanda 1 Health Center, what I know is that it refers people directly to the building next door where examinations and treatment for sleeping sickness are done. But for screening at Katanda 1, I have never seen it.*

**II. Perception of Health Services:**

**When you feel ill, here in the village, where do you go first to seek a solution? (Church, traditional healer, or modern medicine?)**

*As for me, as soon as I feel that something is wrong in my body, I quickly run to the Health Center so that I can be examined and we can know what illness I have and I can receive treatment for my cure.*

*Regarding going to churches or traditional healers, I am the president of community health workers. Our role is to sensitize the community so that in case of illness, the Health Center is the first place where one should start because it is at the Health Center that one can be told what they are suffering from, since that is where examinations, whether of blood, stool, or urine, are done.*

**When you think, based on the signs mentioned (refer back to some signs cited by the group), that a person has sleeping sickness, what do you do to find a solution?**

*If a person shows the signs we mentioned, we send them directly to the CDTC (Sleeping Sickness Treatment Center) for examinations. Because if it is sleeping sickness, care is free; they feed you very well, you pay nothing.*

**How do you assess the services offered by the Health Center you frequent in the village?**

*As for me, I have already frequented various health services with my children and their mothers, some for childbirth, others for treatment. My observation is that the welcome is always satisfactory. It is as if your visits to the centers encourage the healthcare providers to change their behavior.*

**How do you assess the distance traveled to reach the Health Center?**

*For our community in general, the distance is short, so that in case of illness the patient can arrive at the center without difficulty. If someone dies on the way, it's simply because death exists, but the distance is short. About a 25-minute normal walk for someone living in the most remote corner.*

**How do you assess the treatment you receive at the Health Center?**

*There are times when we receive good treatment, but there are also times of concern. Concern, why? It is not related to the quality of treatment, but it's when you come for a specific illness hoping to receive treatment for that illness, but they give you a prescription to go and buy the products. That is where the concerns begin. But regarding the work itself, they do it well.*

**How do you assess the cost of consultation and care at the Health Center?**

*The cost of care is low and appreciated by all, but I know there are also those who are destitute. For example, I am a teacher and I have a field, but you can find people whose lives are really difficult, who even go into debt at the Health Center because of this small cost.*

**Are you aware that screening examinations for sleeping sickness are free?**

*Yes, I am sure of that; it is information that we ourselves disseminate within the communities whenever we talk about sleeping sickness.*

**Is there a problem that prevents community members from frequenting the Health Center for care?**

*The barrier is when the community has no means; at that point, a person might stay home and refuse to go for care because they don't have the means to pay. But our role in the community is to break down this barrier. We sensitize the population to frequent the Health Center even when they have no means. We encourage them to even enter into payment commitments with the center, and the center agrees to give treatment on credit, and the patient honors their commitment after care.*

**What are your suggestions if we need to improve access to health care services in our Health Area/Health District?**

*Suggestions, yes. The management team of the structure needs to be trained, that is, the healthcare provider and the others. Additionally, the community health workers also need to be trained so that together we can get involved in the fight, eliminate this disease, and reduce the number of deaths.*

**III. Perception on HAT Integration:**

**Are you aware that the Health Center of this Health Area had integrated sleeping sickness control activities? If yes, how did you get this information?**

*Yes, I am aware of the integration. It was through the healthcare provider who needed community health workers for training on vector control. It was on that occasion that we were informed of this, and it was on that same occasion that our community health workers were involved.*

*No, regarding integration in terms of screening activities, I am not aware. All I know is that our Health Area refers cases, but for screening, I am not aware.*

**Why is the integration of sleeping sickness screening into this Health Center necessary for the community?**

*Yes, integration is necessary because when the community is sick, it is here at the center they come, and they are accustomed to the healthcare provider and the nuns. If you tell them to go to the other side, they think they will have to spend much more. It is in this sense that integration is beneficial. It is also a way to accelerate elimination because there is synergy in work if we start doing in our centers what was previously only done at the CDTC (Sleeping Sickness Treatment Center).*

**Why, according to you, do some people go to get screened elsewhere for sleeping sickness rather than here?**

*It's simply a matter of preference. Someone might just go elsewhere because they have high regard for that healthcare provider, or because that healthcare provider is their brother, so they will go for favors. That is what I observe.*

**According to you, what should be done to improve access to sleeping sickness screening through Health Centers?**

*I like your question. You see, even in your introduction, the way you presented the statistics with a single case in 2019, we expected it to drop further in 2020. Unfortunately, we find ourselves with 15 cases. This shows that integration will greatly help, in the sense that if someone presents only headaches, we do the examinations. Now, we need to think about supporting community health workers in each Health Area, according to your means, to encourage them to get involved in the fight. There is a lot to be done, especially for trapping.*

**I thank you.**
