# Supplementary material for: Barriers to integration of passive screening for sleeping sickness in Bibanga Health District, Democratic Republic of the Congo
Source: PLoS Negl Trop Dis. 2026 Apr 8;20(4):e0014179. doi: 10.1371/journal.pntd.0014179 (PMC13089886; doi:10.1371/journal.pntd.0014179)
Supplement: S2 File — (ZIP) [file pntd.0014179.s002.zip › S2_Verbatim transcripts/2_AS_KATANDA1/AUD.13_ENT_IT_KATANDA1.docx]

**INTERVIEW WITH HEALTHCARE PROVIDERS OF KATANDA 1**

**Audio N°13: Interview with the IT (Integrated Team)**

**I. Knowledge of HAT (Human African Trypanosomiasis) Control Strategies**

**Could you tell us about the strategies used by your Health Area (HA) to reduce the prevalence of sleeping sickness in the Bibanga District Health (DH)?**

*We have strategies such as environmental sanitation. In other Health Areas, they used RECOs (community relays) for trapping, but I haven't seen that in my HA. That's what I know. Unless our RECOs are trained to start trapping, they could catch flies and bring them to the center.*

*There is screening at our HA level; we have HAT RDTs (Rapid Diagnostic Tests). For every patient presenting with fever or headaches, that's an entry point for screening for sleeping sickness. Sometimes the mobile unit helps us conduct these tests.*

**Q (How is the screening process conducted?)**

*First, the person comes for a consultation. If they have a fever, we start with a malaria RDT. If it is negative and the person still complains of fever, we then proceed with the HAT RDT. Or if the person has persistent headaches, we always perform the HAT test. If it is positive, we now refer the patient to the CDTC (Diagnosis and Treatment Center) for confirmation.*

*Since you were assigned to this Health Area, have you ever diagnosed HAT in your center? If not, why? If yes, how do you do it?*

*Yes, in 2018 I had some positive RDTs, and I referred them. Later, after examinations, they were said to be negative.*

*How? (Follow-up)*

**Have you ever encountered resistance when referring a patient for confirmation of a suspicion made by you? If yes, what did you do to convince them to go to the HAT confirmation center?**

*Our facility is just a few meters from the CDTC. We ourselves go with the patient to present them to the confirmation center for further analysis.*

**II. Perception of HAT Integration**

**In your opinion, when we talk about integrating sleeping sickness control activities into Basic Health Services (SSP), what does that mean to you?**

*When we talk about integration, it means we want to treat sleeping sickness like any other disease, adding it to the other activities we are already carrying out in our facilities.*

*Regarding integration, I see that there is still work to be done, because the disease is still present. There are people who suffer from it but refuse to go where screening is done to know their health status. Sometimes they go into the bush and take the disease there; we thought the disease was already finished. Recently, we have still seen cases; I saw HAT patients at the treatment center. We thought we were nearing the end, but the disease is still there. First, we need to improve the conditions for screening in our communities; then things will get better.*

*The first thing is to support us with many traps. The people in the community can work in the areas where people wander in the bush, where there are forests, to catch the flies. If such techniques are available, I think it could work.*

*The center continues to screen to find patients, but in a way, patients do not present to the health center on time. They always arrive late, at an advanced stage. Because if the center has inputs, it works; the center cannot work when there are no inputs. If there are inputs, the center works. The problem is always in the community. We need to educate and sensitize them so they can come to the center if they see certain signs, if they feel unwell, they can first come to the center so we can examine them.*

*Q (Follow-up)*

**Do you think integrating only screening and diagnosis is sufficient to eliminate HAT in the Bibanga Health Zone, or are other complementary strategies needed? If not, why? If yes, which ones?**

*If you have other activities to add, you can always add them; I don't know of other activities.*

*Patients always come from the community. If we also educate the community to work under such conditions or in such ways, they will understand; the community will always follow.*

**In your opinion, was it necessary to implement these activities precisely at this moment? Why?**

*They were right because our facilities are entry points for diseases; it's there that we can still find sleeping sickness in some patients.*

**In what ways are the HAT control activities implemented in the Minimum Activity Package (PMA) of your health center beneficial to you and your facility?**

*Yes, it is beneficial. If there is a treatment failure at this level, I do screening; the test guides me to do this, and I refer the patient to the right path to find healing. Moreover, the work has improved. Previously, there was one treatment; now there is another. In the past, patients took medication that caused many deaths due to nutrition issues; they took the medication without eating. But recently, we see that we take care of the patients and support them in every way. There is a great change.*

**In what ways are the control activities implemented in the PMA of your health center beneficial to the community of your Health Area?**

*There are benefits because in my HA, apparently, I haven't had patients as we did in the past. They have done us a good service.*

*What do you think about the time you spend screening a suspected HAT case in your health center compared to routine daily activities? Does this time represent a loss of revenue for you and your facility?*

*According to our code of ethics, we are here for the patients, and we work for them. This screening is part of our daily activities.*

*As I said, we are here for the patients; it is not a waste of time for us. Moreover, it is advantageous because I continue to learn a lot from their history.*

*Q (If…—interruption)*

**III. Perception of HAT Elimination**

**In your opinion, what comes to mind when we talk about HAT elimination?**

*Elimination—I think that when we say elimination, it means we no longer find a single positive HAT case, meaning zero cases. But we also need to consider elimination from the program's perspective: when there are no more patients, they will no longer be paid.*

**Do you think HAT elimination is an urgent priority in the Bibanga Health Zone?**

*I don't believe it should be eliminated in the near future. As long as people wander in the bush, and we have flies in the bush, I don't think so.*

*I think that if we proceed gradually, as we are doing, the disease will eventually end.*

**In your view, what is the most effective means to eliminate HAT? Why is this means more effective than others?**

*(……) Yes, if there is a way to find other materials to add to the existing ones, you should always add them and also motivate the people working so they work correctly. If the program manages things properly—for example, with the RDTs, we experienced a stockout for a long time. Only a few months ago did we receive RDTs. When the MCP (Head of the Health Zone) was at a meeting in Bibanga, we raised this issue; she went to get them to supply the Health Zone. That's when we started being supplied.*

*If we want to add things, specialists should add other elements.*

**What do you do at your level to make HAT elimination a reality by the 2030 horizon?**

*So that we no longer speak of sleeping sickness, we must focus on environmental sanitation—that is what I see.*

**IV. Community Accessibility to HAT Screening Services**

**What do you think about the community's attendance at the health center?**

*There are people who suffer but lack the means to reach the health center. There are also those who arrive with whatever little they have, and we always receive them.*

*Some say that they ask for too much money there; that's what I know.*

**What do you think about the availability of HAT screening at your health center?**

*When the tests are not available, it is a barrier to referral. As I told you, we perform the test, and when the test is positive, we refer to the CDTC.*

**How do patients perceive a positive HAT RDT result when they came for consultation suspecting malaria?**

*You can't just tell the person outright like that. You can say that the test I performed shows such and such a sign, and so, to properly confirm this disease, we will accompany you to perform additional tests that we don't have here. There, it will properly confirm what we are looking for.*

**What do you do in a situation where a person refuses to believe the result of a positive HAT RDT after examination?**

*We have never encountered resistance.*

**How do you assess the acceptability of going to a referral facility for confirmatory diagnosis by a suspect with a positive RDT?**

*There is no problem here; the confirmation center and our center are side by side.*

**What prevents the community of this Health Area from accessing the care offered by your health center?**

*I can speak about one barrier. If a person has convulsions at home, instead of bringing them to the center, they prefer to first take them to church or to traditional healers so they can pray or work on them for demonic spirits. This contributes to failures.*

**What do you suggest to improve the community's use of the health center?**

*We will negotiate with the community and find a compromise with them regarding flat-rate pricing. For example, we set a fixed price for children and a fixed price for adults. That is for simple illnesses; for serious illnesses, it will require more resources.*

*My final word is that if you support us with inputs and also train the RECOs in community sensitization, that can help us work properly.*

**Thank you.**
