# Supplementary material for: Barriers to integration of passive screening for sleeping sickness in Bibanga Health District, Democratic Republic of the Congo
Source: PLoS Negl Trop Dis. 2026 Apr 8;20(4):e0014179. doi: 10.1371/journal.pntd.0014179 (PMC13089886; doi:10.1371/journal.pntd.0014179)
Supplement: S2 File — (ZIP) [file pntd.0014179.s002.zip › S2_Verbatim transcripts/2_AS_KATANDA1/AUD.8_FG_FEMMES_KATADA1.docx]

**FG WITH MEMBERS OF THE COMMUNITY OF THE BIBANGA HEALTH ZONE**

**Audio No. 8: FGD with women from the Katanda 1 Health Area**

**I. Knowledge of Sleeping Sickness**

**Do you know a disease that makes the person who contracts it sleep at any time and uncontrollably? What do you call it in your language? What are the different names of this disease and what do they mean?**

*P10: It's sleeping sickness;
P7: The drowsiness;*

**Apart from the fact that the person has uncontrollable sleep at times, do you know other signs attributed to this disease?**

*P10: There are signs; you can see that someone has sleeping sickness if they have intense headaches. In another person, they develop a mental retardation, and if you speak to them, they answer you about something else. If tests are done, they will find sleeping sickness. Another sign: the person sleeps a lot with fatigue;
P8: In other people, cervical lymph nodes are found;*

**Where does this disease come from and how is it transmitted to humans?**

*P2: Someone can catch sleeping sickness from the bite of the sleeping sickness insect called the tsetse fly;
P3: Transmission occurs when this fly bites a sick person and then comes to bite another who did not have it; they also catch this microbe;*

**Are there ways to protect oneself from sleeping sickness?**

*P6: Only with drinkable and injectable products;
P4: In the past, we saw that in places where we drew water, they had installed traps to catch the flies there. Then we were taught that we should always wear white clothing because it repels this insect;*

**II. Perception of Health Services**

**What do you do here in the village when you feel sick? (Where do you go to find a solution?)**

*P9: In case of illness, some people start with indigenous products, and when there is no change, they turn to the health center to find out what the underlying cause is;
P7: When a person falls ill, the first thing is to go quickly to the hospital for tests. When blood, stool, and urine tests are done, the disease is found and treated;*

**If a person thinks, based on the signs mentioned (refer to some signs cited by the group), that they have sleeping sickness, what do you do to find a solution?**

*P3: At the hospital;
P4: At the sleeping sickness center;*

**Do you know the structures that organize or carry out screening for this disease? If yes, which ones?**

*P10: At FEMETRO, by the team that comes with the vehicle;
P8: By the people from FEMETRO who pass through our neighborhoods; they palpate our necks to see those who have lymph nodes;
P6: And also at the secondary hospital;*

**How do you appreciate the services offered by the health center you frequent in the village?**

*P8: We are received well and treated; when you have your money, you are consulted and given treatment;
P2: The service is well organized, and at our place, we are sometimes received even when we don't have the money, but what is lacking is the medication. We are always given a prescription, even for a minor illness;
P1: We are treated well, but there is only one thing lacking: they do not perform tests apart from malaria tests. As for the welcome, I was received even though I didn't even have 10 francs; I paid afterwards.*

**How do you appreciate the distance to travel to reach the health center?**

*P3: The distance depends on everyone's geographical location. Those who are close to the center take less time than those who are far away;
P4: The farthest distance from the center is 30 to 40 minutes on foot;*

**How do you appreciate the waiting time before being received by the health center staff?**

*P5: The waiting time is long. They make us waste time. When you are already there, they look at you as if you weren't there. They go in and out doing nothing while you are sick;
P6: You can arrive at the center with a very sick child; they are there making useless comings and goings. It hurts us very much and it is tiring; that is why we prefer to go to BIBANGA because of that;
P9: Sometimes you can bring a child who is convulsing, and they start making you go back and forth: go get the catheter, go for tests. Sometimes when you cry, they ask you, "What are you crying for, you fool?" while you are crying from suffering. For me, they do not welcome us well;
P4: What is shocking is when you call the nurse to ask them to see how the child is doing, they answer you harshly, "I don't do this job";*

**How do you appreciate the treatment you receive at the health center?**

*P10: Treatment here is the prescription. If you don't have the money to pay for your prescription, you're finished; your problem is not treated;
P9: Even if the medication is in the pharmacy, if you tell them, "Treat me first, I will pay," it's no. Even if you tell them, "Keep this phone, I will come back for it with the money," for them, it's only money. It can happen that you even lose your patient when they could have cured them and asked for the money afterward;
P5: I will add to what they just said about the medication that is in the pharmacy but they refuse to give. They are also right, let me explain: I can ask the nurse to treat my child, and I will come and pay. And when the treatment starts, the child feels a bit better, and we flee the hospital without paying. Who is left to pay for me? Is it the nurse or the one who fled? That is why we who come for care and the caregivers need to have consistency between promises and actions;*

**How do you appreciate the availability of the nurse at the health center when you need them?**

*P8: Yes, the availability is good. As soon as you arrive, the caregivers are always there;
P7: There are times when it is empty. I myself came once and found no one there, especially at night;
P10: I once found a child who had just come from school convulsing during the day. I brought them to the health center and there was no one there at that time. I went to look for the nun at the convent to help, and when she came, she gave an injection to calm the child, you see. And if she hadn't been present at the convent... Afterward, I went to get their parents;
P6: Sometimes we find them, and sometimes we find no one;
P2: The problem is when they are not there. We call them on the phone, but instead of coming quickly, they walk slowly and can even take an hour;*

**How do you appreciate the cost of consultation and care at the health center?**

*P1: For me, it's too much. It needs to be lowered;
P3: The consultation price, we want it lowered;
P8: For me, I am not in favor of this price; I want it lowered;
P5: I find they are right because these papers are printed materials; to get them, they also spend money. What we pay is 1000 francs. If it is lowered, how much will we pay? For me, this price is acceptable;*

**Are you aware that tests for sleeping sickness screening are free?**

*P4: It's free; we don't pay;
P6: It's free all the time, even when FEMETRO comes by;*

*Is there a problem that prevents the community from frequenting the health center for care?*

*P8: Lack of money can prevent someone from frequenting the health center;
P9: When someone doesn't have money, as we said earlier, how will they come to the center because they won't be treated for free?;
P5: Also negligence, because negotiation is also possible. We are human. The nurse, when you speak to them nicely and reassure them that you will come and pay, they can understand and do it. Now, if you are negligent and stay at home, how will it work? But there are people who loiter at home. Only when you come to tell them, "You are sick, why are you loitering at home? Come on, let's go to the health center," it is then that they become aware.* **What is that? Is that not negligence?;***P1: Sometimes there are people who tell us that so-and-so does not want to go for care because the caregivers do not use respectful language towards us;*

**What are your suggestions if we need to improve access to health care services in our Health Area/Health District?**

*P6: Firstly, we want there to be consideration; we want to be considered as responsible people as well, because each of us has our own household. Secondly, we want there to be medication in the pharmacy. That way, if we are given a prescription, we can buy from them instead of going to the market.
P9: For me, I only ask that if a patient doesn't have money on the spot, they can treat them first to even calm the fever. And if someone asks to leave an object first, treat my child, that the caregivers have a good heart to understand us. We don't always have money in our houses every day.*

**III. Perception of Sleeping Sickness and Screening**

**How do you feel in the community if you are told that a certain person has been diagnosed positive for sleeping sickness after the tests?**

*P3: For me, it is a feeling of satisfaction because they will be treated in time and the disease will come to an end;
P4: I agree with what they said;
P8: Sometimes the feeling is sadness; we even ask ourselves questions to know why this disease;
P6: This disease makes one go mad. A person who was with you yesterday, you see them with their clothes in their hands. It hurts, and you even ask yourself questions to know if they have also reached this stage, and who will take care of their children;*

**To what do you attribute the fate of sleeping sickness? (What do people say causes it?)**

*P7: Some say that this disease originates from witchcraft;
P9: Sometimes we ask ourselves questions: how can a person who does not work in their fields in the deep forest where there are insects that bite and transmit the disease catch it? That is why they say it is witchcraft, but for me, that's not it.*

**Does sleeping sickness cause fear when you hear about it?**

*P7: It causes fear. For example, my husband, they had this disease in their family. Four people from the same parents had this disease; two died directly from this disease. Now, the memory of what happened scares me when sleeping sickness is mentioned. Moreover, the needle used for the lumbar puncture scares me. For the survivors of this disease, we suffered a lot with them because of the restrictions (taboos). All of this still scares me when sleeping sickness is mentioned;
P10: The fear does not only come from what is recounted, as we have just heard, but the name of this disease is frightening, even if you are not sick. Once, the FEMETRO team found me with lymph nodes on my neck. When they did the puncture (lymph node aspiration), they didn't find the disease, but I had pain in my neck for nearly six months. At their next visit, I asked them to do a lumbar puncture on me, and that disturbed me too much, to the point that I am still afraid today. That is what creates the fear;*

**Do you think you would go to be screened at a health center/general referral hospital when you present signs that suggest sleeping sickness?**

*P8: I would accept because when you are already at the hospital, you are obliged to follow everything you are told;
P3: I must accept and go so that they can examine me, and if it is the disease, they treat me so that I can be cured;
P4: I also must accept; I will get myself examined;
P10: This disease makes one stubborn. It's enough that they say you have sleeping sickness; you get angry and start arguing with the caregivers: "You saw me with this disease? You are the ones who put this in my body. This is witchcraft. I am going home." The person accepts with difficulty;*

**Why, in your opinion, are some people afraid to be screened for sleeping sickness?**

*P5: They are afraid of the injection;
P7: They are also afraid of prolonged rest without working. If they find me with this disease, my activities will stop.*
