# Supplementary material for: Barriers to integration of passive screening for sleeping sickness in Bibanga Health District, Democratic Republic of the Congo
Source: PLoS Negl Trop Dis. 2026 Apr 8;20(4):e0014179. doi: 10.1371/journal.pntd.0014179 (PMC13089886; doi:10.1371/journal.pntd.0014179)
Supplement: S2 File — (ZIP) [file pntd.0014179.s002.zip › S2_Verbatim transcripts/2_AS_KATANDA1/AUD.9_FG_HOMMES_KATANDA1.docx]

**FG WITH MEMBERS OF THE COMMUNITY OF THE BIBANGA HEALTH ZONE**

**Audio N°9: FGD with men from the Katanda 1 Health Area**

**I. Knowledge of Sleeping Sickness**

**Do you know a disease that makes the person who contracts it sleep at any time and uncontrollably? What do you call it in your language? What are the different names of this disease and what do they mean?**

*P1: When we see a person with a disease that makes them sleep deeply and uncontrollably, we say they have sleeping sickness?*

*P4: The disease of drowsiness;*

*P3: In French, it's called sleeping sickness;*

*P7: Yes, there is a meaning, because for every person there is a time when they must sleep, which is at night. So it is called "lubunga" or "tulu" because you can see the person dozing or sleeping throughout the day. In our language, these are synonyms; when a person sleeps deeply at a time not intended for sleep, we say they have sleeping sickness;*

*P8: Sleeping sickness means that this person sleeps at any time; they have no fixed hours for sleep; they sleep constantly and experience fatigue;*

**Apart from the fact that the person experiences uncontrollable sleep, do you know other signs attributed to this disease?**

*P9: You will see that this person has intense headaches, they cry because of it, and they lose weight;*

*P5: Another sign is madness; the person starts talking to themselves;*

*P2: Fatigue;*

*P1: In addition to what has been mentioned, we also see the person leaving the community to live in the forest; we start looking for them to restrain them;*

**Where does this disease originate, and how is it transmitted to humans?**

*P10: It comes from the fly called the tsetse fly and is transmitted when the tsetse fly bites you;*

*P8: It also comes from bushy areas like the forest. In these places, the flies hide, and when people pass by, this fly can bite them and transmit sleeping sickness;*

*P7: It can also be transmitted by mosquitoes;*

*P6: It is a specific fly that carries this disease; not all flies. The fly that has bitten an animal with this disease is the one that transmits it when it bites a person;*

**Are there ways to protect oneself from sleeping sickness?**

*P8: Yes, we must avoid being bitten by this fly. We should wear a white shirt when we go to the field or to the river where there are flies.*

*P4: Here in our community, we require the community to clear the underbrush where they work, because these flies hide where there is an accumulation of many trees and grasses. Additionally, we need to request the traps we had before; now I no longer see them.*

**II. Perception of Health Services**

**What do you do here in the village when you feel ill? (Where do you go to find a solution?)**

*P3: As I am a RECO [Community Health Relay], it is we who refer people to the Health Center for examinations and treatments; we go to the Health Center;*

*P1: As we are RECOs, if we notice signs of illness in someone, we inform the center; the center sends people to assess the case, or we refer the case to the center, and if it is found to be sleeping sickness, we refer them to the sleeping sickness hospital;*

*P4: As a neighborhood chief, in all the meetings we hold, even at the territory level, we are taught that if a person is sick, even if they want to go to church for prayer, after the prayer they must be referred to the Health Center for appropriate care. We cannot find a sick person interned at the church; there was a time when we recorded many deaths in churches; they were kept there for prayer, but the illness was not known;*

**When you think, based on the signs mentioned (recalling some signs cited by the group), that a person has sleeping sickness, what do you do to find a solution?**

*P3: First and foremost, we refer such a person to the Health Center for confirmation of sleeping sickness, because here in our community, sleeping sickness has killed many people. So these signs prompt us to refer such a person to the Health Center;*

**Do you know the structures that organize or carry out screening for this disease? If yes, which ones?**

*P10: There is the mobile team that moves from one neighborhood to another, examining the community. If they find the disease, they send you to the sleeping sickness center for treatment.*

*P3: We, the RECOs, if we find a person showing signs, we refer them to the trypanosomiasis hospital for examinations, because someone who has the parasite of the disease, you will see they have headaches. If you go to the trypanosomiasis hospital, you will find benches for patients and you sit down; they will ask you what you want, and if you explain, they will let you in for examinations. If they find the disease, they keep you for treatment, and if they do not find it, they will tell you to return home;*

*P4: What we know here is that if we see signs suggestive of sleeping sickness, we refer to the sleeping sickness hospital because the staff there are very experienced in sleeping sickness;*

**How do you appreciate the services offered by the Health Center you attend in the village?**

*P6: (……) Yes, when a person arrives at the center for examinations, they are received by the staff, and they are asked to pay for the medical record, or they are seated somewhere, and when the staff finishes what they are doing, they receive the person, conduct the examinations, and prescribe treatment. Treatment is not given before knowing the illness;*

*P1: I understand. As a RECO, we each have assigned households. When we visit households, if I find a case of illness, I sensitize them and take them to the Health Center. The reception is always good. If there is a debt, I personally sign a discharge because I know the patient's address; at the appropriate time, I find the person and collect the money to pay the debt according to the commitment. For us, the service offered is truly good;*

**How do you appreciate the distance traveled to reach the Health Center?**

*P4: Regarding distance, all distances are normal because in our territory, the distribution of Health Areas was done in such a way that distances are not long so that in case of problems, everyone can manage to reach the center;*

**How do you appreciate the waiting time before being received by the Health Center staff?**

*P10: Usually, even when we are in class, we take attendance; we follow alphabetical order, name by name, until your turn comes for your name to be called. It is the same when we come to the center or the hospital; we find those who arrived before us, and we wait for our turn to be received. The waiting time does not really pose a problem;*

*P5: I add this: the caregivers receive the patients very well because in our community, we organize meetings, and when there is a problem, we raise it and discuss it, and then we bring the amendments to the IT [referring to a health official] so they can correct it. So there is no issue on this side;*

*P6: Here we have a reception service; as soon as a person arrives, they are received, and the medical record is created. After that, the patient goes for consultation and to the laboratory for examinations, then to the treatment room where they will receive their treatment;*

**How do you appreciate the treatment you receive at the Health Center?**

*P4: We are satisfied with the treatments. Here, if the caregivers find that they cannot treat this illness, they send you to the hospital, but other cases they treat very well;*

**How do you appreciate the availability of the Health Center nurse when you need them?**

*P8: The caregivers here are always present when we come. They have divided the working hours so that the facility is never without a caregiver. When we come, we know that there are caregivers; there are no days when we find the center without a caregiver;*

*P9: I would also like to mention something: the caregivers have a set schedule so that the one who comes in the morning replaces the one who worked the night shift. Around 1:30 PM, they are also replaced by another nurse who will work the evening shift, to be relieved by the night shift nurse;*

**How do you appreciate the cost of consultation and care at the Health Center?**

*P10: It depends on the financial means of each person, but the price of the consultation record at our center is affordable for everyone; it neither increases nor decreases; it is always 500 francs [Congolese francs];*

*P7: On this point, the price of the record is acceptable; it does not bother us;*

**Are you aware that screening examinations for sleeping sickness are free?**

*P1: We know because when the mobile team comes to screen the population, it is we who announce and sensitize the community. It is free; they ask for neither a paper nor anything else;*

*P2: It is free. What I am telling you, I experienced myself with my neighbor who was sick. When he was examined at the sleeping sickness center, he was received free of charge; the treatment and even food were free. A good meal, better than what he eats at home;*

**Is there any problem preventing the community from attending the Health Center for care?**

*P9: The obstacle in the community comes from financial difficulties; lack of means. Someone can die simply due to lack of means. They are sick but lack even the 500 francs to come to the Health Center, and we have already seen this;*

*P7: As he just said, a person can lose their life because they lack 100 francs to buy tablets at the pharmacy. If they lack 100 francs, how can they have 500 francs for the medical record?*

*P10: I tell you what I know. Due to inability, there are people in the community who are treated with roots and plants at home. They do not come to the Health Center due to lack of means. Others are in churches. This is why I greatly appreciate the new system where the State helps us; we see the suffering of people who suffer from sleeping sickness decreasing. It is good if you advocate for us regarding other illnesses; otherwise, people will die due to lack of means;*

**What are your suggestions if access to health care services in our Health Area/Health Zone is to be improved?**

*P4: For the health of our community in general, if the State could take measures like those taken for sleeping sickness where total care has become free. We want this care in all our Health Centers: free treatment, free medication. Then the population will not delay at home when they are sick, and deaths in the population will decrease;*

*P3: We want training on awareness-raising for sleeping sickness; many people are still ignorant. As we do for pregnant women, we must do it for this disease;*

*P6: We also want the number of trappers to be increased and for them to be motivated to reduce the flies;*

**III. Perception of Sleeping Sickness and Screening**

**How do you feel in the community if you are told that a certain person has tested positive for sleeping sickness after examinations?**

*P4: When we hear this news in the community, everyone is sad; it pleases no one. Our wish is that when the mobile team passes through for screening, they find nothing; that brings joy. But if we hear that so-and-so's child or such-and-such a person has this disease, it frightens us all. We do not want this disease to still be here.*

*P8: This disease killed my wife, who was treated at Kasansa when they were treating with ARSOBAL [melarsoprol]. For me and for many people, it is a demoralizing disease. If sleeping sickness is detected even in your child, you start praying that they do not die. That is how we think; it is not really a good disease.*

*P9: Also, when we hear that so-and-so's child has this disease, we are worried and we ask ourselves the question: where did they get it? Do they go to the river early in the morning? Do they work in bushy areas with too many palm trees? This disease truly disturbs us.*

*P6: We do not want to hear about this disease. There are people who have never seen its effects, but only hear about it; we have experienced it closely. Let this disease end.*

**To what do you attribute the fate of sleeping sickness?**

*P5: Take my example. I farm between two rivers, Mutuayi and Bufua. I am at high risk because there are many trees and undergrowth. Sometimes I find myself alone working in the field, and at times I am surprised to be bitten by insects, the fly we call here at the center the "neglected tropical disease." We have many of them, and they bite us. That is how you can one day find that this fly has bitten you. We do not work in good places.*

*P3: This disease here in Katanda is common, and we all know that it exists. Even if it catches someone, we know it exists.*

*P6: The regret we have when a person has this disease, we ask ourselves if it is some demon, truly some demon that comes to cause this difficulty. But we know it comes from the fly; it is just a way of exclaiming, "Kaaa, what demon is that!"*

*P8: Sir, we were already shown that when we work in places that are not good, we should wear white shirts to avoid these insects, because when they see the color white, they flee and go away. If they see you in dark clothing, they come to bite you. That is why many people use white shirts in the fields to avoid this disease. And many people were afraid when they saw at the sleeping sickness center the number of people suffering from this disease.*

**Does sleeping sickness cause fear when you hear about it?**

*P10: Yes, it causes fear. The way we saw people with this disease frightened us greatly, but the system that the State has recently put in place has succeeded.*

*P9: It is truly a success; we are no longer afraid. Even if I catch it now, I am no longer afraid.*

*P6: We were afraid of the way people died and of the way Arsobal killed even more than the disease. Now, with what the Congolese State has done for treatment, we are no longer afraid. Even if a person is struck by this, they will run to the healthcare staff and receive good treatment.*

**Do you think you would go to be screened at a Health Center/General Reference Hospital if you present with signs suggestive of sleeping sickness?**

*P8: Currently, I would go for screening with joy. I lost my wife because of that product, ARSOBAL. I took her to Kasansa in good condition and returned from there with a corpse. But now the disease is easily curable.*

*P4: Now people have the courage to go to the Sleeping Sickness Center for screening. Before, it was that product, ARSOBAL, that caused fear. In my neighborhood, there are children who experienced stunted growth due to ARSOBAL. Now, with the system of new and good products, in addition to free care, it is good. People were truly afraid. When it was announced that the FOMETRO agents were there, some would not come; they said, "Your people who carry that disease..." and they would go and hide. Currently, when we finish the day with nothing found, the population is happy, and we too, no one is afraid.*

**Why, in your opinion, are some people afraid to be screened for sleeping sickness?**

*P5: They are afraid because they still have the old system with the old products in mind.*

*P6: Others are afraid of the lumbar puncture; this injection is very painful.*

*P8: When you see people who are afraid of a simple injection, only the lumbar puncture... this also causes fear because of everything involved; if you are punctured, you will spend many days unable to straighten up.*

**Thank you.**
