# Supplementary material for: Barriers to integration of passive screening for sleeping sickness in Bibanga Health District, Democratic Republic of the Congo
Source: PLoS Negl Trop Dis. 2026 Apr 8;20(4):e0014179. doi: 10.1371/journal.pntd.0014179 (PMC13089886; doi:10.1371/journal.pntd.0014179)
Supplement: S3 File — (ZIP) [file pntd.0014179.s003.zip › S3_Verbatim transcripts/3_AS_TSHILULA/AUD.15_FG_FEMMES_TSHILUILA.docx]

**FGD WITH MEMBERS OF THE COMMUNITY OF THE BIBANGA HEALTH DISTRICT**

**Audio No. 15: FGD with Women from the Bakwa Tshiluila Health Area**

**I. Knowledge of Sleeping Sickness**

**1. Do you know a disease that causes a person to sleep uncontrollably at any time? What do you call it in your language? What are the different names for this disease and what do they mean?**

*P9: Sleeping sickness;*

*P8: What is known is sleeping sickness;*

*P5: It's malaria; it makes you sleep when you are undergoing treatment;*

*P1: The sleeping microbe;*

*P9: It is called sleeping sickness because it makes you sleep at any time;*

**Apart from the fact that the person experiences uncontrolled sleep, do you know any other signs attributed to this disease?**

*P10: The most prominent sign is drowsiness and a dazed look;*

*P7: The person loses memory and becomes like a mad person;*

*P6: Weakness;*

*P4: The person starts talking excessively and exhibits behavioral disturbances;*

**Where does this disease come from and how is it transmitted to humans?**

*P1: It is the fly; it comes from flies that land on unsanitary places and then land on humans;*

*P2: When these flies land on the food we consume;*

*P3: When one does not wash their hands;*

*P6: Transmission occurs when one uses the same cup to drink water without cleaning it;*

*P7: Also when eating from the same plates;*

*P9: In bushy areas, the insects that are hidden there, if they sting, that is how they transmit the disease;*

*P5: Also through mosquito bites;*

*P10: In the field where we work, there are large flies with two colors, green on the head and yellow on the abdomen. Once you see this fly, you should not even allow it to come near you. For us farmers, you must even abandon that field and start a new field elsewhere;*

**Are there ways to protect oneself from sleeping sickness?**

*P10: For me, I would say, as I just told you about this fly, you cannot do otherwise when you see it in the field; you simply have to flee that place to avoid it;*

*P1: Remove dirt around the house, wash, and organize belongings to prevent the presence of these flies and keep them away. It is in this way that we can avoid the disease;*

**II. Perception of Health Services**

**What do you do here in the village when you feel sick? (Where do you go to find a solution?)**

*P2: When I fall sick, for me, there is no other place to start. I go directly to the health center to find out what is bothering me;*

*P3: When illness begins in a person, the person rushes to the hospital. There, one is examined to discover all the diseases operating in the body. There is no other place to start;*

*P6: When I have a sick child, for example with a fever, I do not rush quickly to the health center for that. I might first buy Novalgin and give it to them, and if there is no change afterward, that is when I have to go to the health center for treatment;*

*P4: Sometimes the person also goes to church so that prayers can be said for them;*

**When you think, based on the signs mentioned (recall some signs cited by the group), that a person has sleeping sickness, what do you do to find a solution?**

*P7: When the signs of sleeping sickness are already present, we take the person and bring them to the hospital so that they can be examined to discover the source of the problem, and we try to treat them to calm the situation;*

*P6: Others go to church;*

*P5: Those who go to church do so afterward, when modern medicine finds no solution and the disease is unknown. Otherwise, we always start at the health center to seek to understand what is bothering them;*

*P9: When there are behavioral disturbances, we seek to know the origin. If it is a normal disease, we treat it; if the origin is, for example, that the person had touched fetishes, the solution lies with the fetish healer or at church;*

**Do you know the structures that organize or conduct screening for this disease? If yes, which ones?**

*P4: It is at the health center, and if at the health center they do not discover the illness, they issue a referral note to the large hospital;*

*P5: We take them to FEMETRO for examination; that is the major center we know;*

*P1: If we see signs of sleeping sickness, we can also go to Bibanga; there we must seek to know if it is sleeping sickness or another disease;*

*8. How do you assess the services offered by the health center you frequent in the village?*

*P6: Thank you for the opportunity to speak. The service, yes, I can say it is good, but there are too many weaknesses on the maternity side. We need to renew the team of birth attendants. Those we have do not work well and are inexperienced. Sometimes a woman comes to give birth, but there are insults and negligence. Sometimes the parturient gives birth all alone, and the attendant comes only to tidy up the baby. There is no proper welcome;*

*P2: I will speak as you have designated me. In this health center, the welcome is good, but only for examinations. Apart from blood tests, others like stool and urine tests lack materials; it is a hardship for us;*

*P7: It is always the problem of examinations. Look, since I started treatment, there has been no change. Why? It is because no other examinations were done to know where the disease is hiding and to treat me. Since they did the blood test, they do not know how to do other examinations to see inside the abdomen, but I continue to take the treatment. Now, what treatment are they giving me? That is truly the hardship we have;*

*P8: We have services without a doctor, and this poses a difficulty because our nurses are groping their way through treatment. If doctors could be sent to us, even if they come twice a week;*

*P9: We once received a doctor who came when we were given tokens. We found many diseases that were hidden, and the whole community was happy. If you do that again, it would be good;*

*P3: Doctor, look at me. I came for treatment, but the health center has no medications. I was given a prescription, and I bought this product in Katanda, but I still pay other fees here. If they had a pharmacy with products, it would be good. Let them provide products on site. For now, even if your illness is severe, they will give you a prescription to buy products in Katanda or Kalambayi, and if you do not have the money, what are you going to do?*

*9. How do you assess the distance traveled to reach the health center?*

*P7: For example, I take one hour to reach the health center. I have to take my treatment in the morning and evening; it is too much. There are even others who live farther than I do;*

*P10: The distance depends on the village where you live. I am in Bakua Tshiluila, which is very close to the center, but others, for example from Bakwa Njiba, are too far;*

*10. How do you assess the waiting time before being received by the health center staff?*

*P1: If you find many people, you will wait, but there are days when there are not many people. Even if there are many people, if you came before others when they start, you will not wait;*

*P7: Sometimes we are made to wait for other people. They tell you that you must wait until there are three or four people, then they will receive you. It sometimes takes a long time because you do not even know when the others will come. It can take even 30 or 45 minutes;*

**How do you assess the treatment you receive at the health center?**

*P6: What can I say about the treatment? Sometimes we are treated well, but there are also times when you come with a child, they do everything, and there is no change. They give you a paper, and you leave for Bibanga;*

*P9: The problem is not our nurses, but at the health center there are no doctors, no X-ray, no ultrasound. How are they going to see other diseases that are hidden?*

*P8: My son has been here for a week now; apart from the fever, I see no change;*

*P2: Sir, all this is because there is no laboratory. We are treated like this by trial and error. They do everything based on your explanations. When you say the child had a fever all night, they take the product for fever and inject without knowing what caused the fever. You see, if they have not given the product that reaches the site of the disease, how will the person heal? It could be another disease, but they give the product for a different disease;*

**How do you assess the availability of the health center nurse when you need them?**

*P3: One time I came at night; I found only the birth attendants at the health center. The caregiver had spent the night at home; we went to fetch them;*

*P10: When you come at 6:00 PM, you will find no one. They say you came late;*

*13. How do you assess the cost of consultation and care at the health center?*

*P4: They ask us for 5000 francs; that is what is even displayed on the wall. But when you add the prescription, it becomes a lot;*

*P6: This amount is too expensive; we don't have that kind of money every day;*

*P3: It exceeds our means, especially for us who have prescriptions. We pay the same amount as those who do not have them. They receive their products, but we bring our own products and still pay like the others;*

**Are you aware that screening tests for sleeping sickness are free of charge?**

*P10: We are aware of that;*

*P8: We are informed;*

*P7: I do not know how it works;*

*P3: We know; my son was treated in Bibanga, and I did not pay even for the bed;*

**Is there a problem that prevents the community from frequenting the health center for care?**

*P1: Yes, a person may be prevented by lack of means. Here, we depend on the products of the field. In this period when we have ripe corn in the field, it is good. After this period, it is the kasua banga (lean season). In that period, even 200 francs poses a difficulty for the community;*

*P7: For me, because of the distance, when we are sick, we first seek a solution locally. We buy the product; if the illness does not go away, we come to the health center. We come only when there is no change or in case of severity;*

**What are your suggestions for improving access to health care services in our health area/health zone?**

*P6: My suggestions are as follows: first, that they send us doctors and also send us other birth attendants. We want those who do not insult us and who work well;*

*P9: We also want them to have ultrasound and X-ray to see diseases that are hidden;*

*P8: We want doctors because when they do not find the disease, they send us too far away with the risks along the way. Sometimes the patient loses their life even before we arrive;*

**III. Perception of Sleeping Sickness and Screening**

**How do you feel in the community if you are told that a person has tested positive for sleeping sickness after examinations?**

*P5: It worries us. We ask ourselves the question of where this bad disease comes from. It is a bad disease because, you see, you treat someone, and after their treatment, they remain with sequelae. That is why I say it is a bad disease;*

*P4: I do not feel well; I ask myself how this disease could catch such a person. It makes me uncomfortable;*

*P2: Sir, it makes us feel regret. You know that if this disease catches a man, his whole body is fatigued, even his genital organs. The man will no longer be close to his wife. That is why it worries us;*

**To what do you attribute the fate of sleeping sickness?**

*P9: This disease comes from the bite of a bad insect;*

*P7: There is the normal disease as she said, but there is also bad luck from sorcerers. Even if treated, there is no cure;*

*P10: As was just said, the disease that comes from God, when treated, the person heals. But the one that comes from people of the world of darkness, even if the caregivers do what they can, there is no cure;*

**Does sleeping sickness frighten you when you hear about it?**

*P4: Yes, it is a frightening disease because it leaves you nine to six (i.e., at death's door);*

*P8: Because the person becomes mad;*

**Would you consider going to be screened at a health center/general referral hospital when you present signs suggestive of sleeping sickness?**

*P9: I will accept and ask God to help me so that I may heal;*

**Why do you think some people are afraid to get screened for sleeping sickness?**

*P6: They are afraid. Some say that the sorcerers who are present will cast a spell on me so that I am declared positive;*

**Thank you.**
