# Supplementary material for: Barriers to integration of passive screening for sleeping sickness in Bibanga Health District, Democratic Republic of the Congo
Source: PLoS Negl Trop Dis. 2026 Apr 8;20(4):e0014179. doi: 10.1371/journal.pntd.0014179 (PMC13089886; doi:10.1371/journal.pntd.0014179)
Supplement: S3 File — (ZIP) [file pntd.0014179.s003.zip › S3_Verbatim transcripts/3_AS_TSHILULA/AUD.16_FG_GARCONS_TSHILUILA.docx]

**FGD WITH MEMBERS OF THE COMMUNITY OF THE BIBANGA HEALTH DISTRICT**

**Audio N°16: FGD with boys from the Bakwa Tshiluila Health Area**

**I. Knowledge of Sleeping Sickness**

**Do you know a disease that makes the person who catches it sleep at any time and uncontrollably? What do you call it in your language? What are the different names of this disease and what do they mean?**

*P1: Sleeping sickness;
P4: The disease of drowsiness;
P5: Sleeping sickness means a disease that makes you sleep incessantly;
P4: It is the disease that prevents people from being active; the person does nothing but sleep, even while working;*

**Apart from the fact that the person has uncontrolled sleep at times, do you know of any other signs attributed to this disease?**

*P6: The person becomes loquacious (talks excessively);
P7: (......) It causes physical weakness;
P8: The person no longer has the strength to work;
P2: The person exhibits behavioral disorders;*

**Where does this disease originate and how is it transmitted to humans?**

*P5: The disease comes from insects found in our forests or from mosquitoes that bite and transmit the disease;
P6: There are insects that bite, or even mosquitoes, that bite a sick person and then go on to bite a healthy one;
P8: There are flies that we call the sleeping sickness fly; this fly can land on the feces of a person who has sleeping sickness and then come and land on food; that is how it transmits the disease;
P1: It is this fly; when it bites someone, it injects a fluid that contains the microbe of sleeping sickness;*

**Are there ways to protect oneself from sleeping sickness?**

*P9: To avoid this disease, you should eat your meal while it is still hot;
P10: You should sleep in a good place, under a mosquito net, to avoid mosquito bites;
P1: You must avoid the bush on our plots of land;
P5: Since these flies live near water and in overgrown areas, you need to clear these areas and maintain the paths leading to them to avoid the flies;
P2: We must avoid staying near these places where the flies live;*

**II. Perception of Health Services**

**What do you do here in the village when you feel sick? (Where do you go to find a solution?)**

*P1: It happens like this: when the illness just starts, we might go to a pharmacy and buy medicine, or we might use herbal teas; if the fever continues and there is no change, we go to the health center (CS);*

**When you think, based on the signs mentioned (reiterate some signs cited by the group), that a person has sleeping sickness, what do you do to find a solution?**

*P5: We take them to the hospital for tests to find out what the underlying cause is;
P8: We ask them to go for care and treatment, but if they lack the means, they may refuse to go and seek other solutions;*

**Do you know the structures that organize or carry out screening for this disease? If so, which ones?**

*P10: The structure in our area is FOMETRO in KATANDA;
P9: There is also the FOMETRO team that comes every year; if we have a patient with the signs, we take them to this team;
P4: If we see our friend with the signs, we do not take it upon ourselves to take them to KATANDA; we have our health center here; it is the nurse who will refer them to FOMETRO.*

*How do you appreciate the services offered by the health center (CS) you frequent in the village?*[No response recorded in the transcript]

**How do you appreciate the distance to travel to reach the health center (CS)?**

*P8: There is no long distance because in every area far from the health center there are Community Health Workers (AS) like in Nkimba; and in a village up on the hilltop, there are Community Health Workers who support this center;
P1: For us, who are beyond Nkimba, the distance to reach the center is enormous;*

*How do you appreciate the waiting time before being received by the health center (CS) staff?*

*P6: Here, as soon as you arrive, you are received and treated;
P3: The welcome is good; as soon as you arrive, you are received, a file is created, and they start asking you questions about the illness. You might be kept waiting a little, but it is not a long time;*

**How do you appreciate the treatment you receive at the health center (CS)?**

*P5: We receive good treatment; although I have never come for treatment myself, I accompany sick people; they receive good treatment;
P6: The treatment is good for most cases;
P1: The treatment is good, but for cases of anemia, the transfusion poses a problem in that the interventions are carried out with a lot of delay. Donors to help anemic patients are not available, and the center does not have the product for that;*

**How do you appreciate the availability of the nurse at the health center (CS) when you need them?**

*P10: On this point, it is especially with the nurses; if they go home to come back here, it is a problem;
P9: Whenever I come to the center, I find the caregivers; it poses no problem;
P6: For us, the nurses are always present permanently, at any time;
P3: The work is shared according to the hours; there is the one who works in the morning, they are replaced around noon, and the one who comes at 6 p.m. works until the morning;*

**How do you appreciate the cost of consultation and care at the health center (CS)?**

*P2: The cost is affordable;
P5: It depends on each person's means; some people find it expensive, and there are still people who think that even the 500 francs for the registration form is a lot; but others, on the other hand, find this price affordable;*

**Are you aware that the tests for sleeping sickness screening are free?**

*P1: It is known to everyone; we do not pay;
P4: I know that we do not pay;
P3: I do not know;*

**Is there a problem that prevents the community from frequenting the health center (CS) for care?**

*P7: Yes, a person may be sick and want to get treated, but they lack the means to come to the center;
P8: Someone may be sick but lacks someone to bring them for care if they are too weak to walk alone;
P2: Someone may be prevented from frequenting the center by the fact that the treatment received the last time at the center was unsatisfactory, and they were referred to Bibanga without being cured; as they lack means, they think going to the center would be a waste of time while waiting for the means to go to Bibanga where the care is of better quality.*

**What are your suggestions if we need to improve access to health care services in our Health Area (AS)/Health District (HD)?**

*P9: The first thing: if we could think about equipping our laboratory;
P3: Also, the center does not have a laboratory technician;
P5: We want a doctor to be assigned to us to start performing surgeries/operations on us;*

**III. Perception of Sleeping Sickness and Screening**

**How do you feel within the community if you are told that a certain person tested positive for sleeping sickness after the examinations?**

*P10: I feel very bad when I hear this, because my friend who has this—if the mosquito bites him and then comes and bites me too, you see what that means. It scares me when I think about it;
P6: Also, since it's a friend, if I share the same glass of water with them, they could transmit the disease to me;
P5: It worries me a lot because this disease is transmissible even if we are not friends; the fact that the fly can bite that person and then bite us;
P8: It pains me because the person can be left with after-effects; they can lose their senses; because I see many people who have suffered from this have their minds affected; that is why it worries me;*

**To what do you attribute the nature of sleeping sickness?**

*P1: It is a disease that is natural; it is God who created all diseases;
P5: I say it is not natural; insects are the cause, and it can be cured. On the other hand, AIDS can be treated but is not curable; that is what we can call a natural disease;*

*Does sleeping sickness frighten you when you hear about it?*

*P4: It frightens you when you have already seen the effects in a person who has suffered from it;*

**Do you think you would go to get screened at a health center (CS)/general reference hospital (HGR) if you show signs suggestive of sleeping sickness?**

*P3: I would go get tested to have confirmation, and if it is the case, I would be treated;
P6: I would accept because we need confirmation, and if it is that, I need to be treated;*

*Why, according to you, are some people afraid to get screened for sleeping sickness?*

*P7: It is fear of the lumbar puncture (the "CSF" injection/needle);
P8: Others refuse because they lack means and think that in Katanda, you need to have food, whereas I do not have any;
P5: There are also people who have never received an injection since birth; with what is said about the size of the lumbar puncture needle and how painful it is, they are afraid.*

**Thank you.**
