# Supplementary material for: Barriers to integration of passive screening for sleeping sickness in Bibanga Health District, Democratic Republic of the Congo
Source: PLoS Negl Trop Dis. 2026 Apr 8;20(4):e0014179. doi: 10.1371/journal.pntd.0014179 (PMC13089886; doi:10.1371/journal.pntd.0014179)
Supplement: S3 File — (ZIP) [file pntd.0014179.s003.zip › S3_Verbatim transcripts/3_AS_TSHILULA/AUD.17_FG_FILLES_TSHILUILA.docx]

**FGD WITH MEMBERS OF THE BIBANGA HEALTH DISTRICT COMMUNITY**

**Audio No. 17: FGD with Girls from the Bakwa Tshiluila Health Area**

**I. Knowledge of Sleeping Sickness**

**Do you know a disease that makes the person who catches it sleep uncontrollably at any time? What do you call it in your language? What are the different names for this disease and what do they mean?**

*P3: It's sleeping sickness;
P4: It's the disease of drowsiness;
P2: It's a disease that makes you sleep and the person loses consciousness;
P6: It's also because the person cannot control themselves and dozes off at any time;
P8: The person dozes off even while they are cooking, that's why it's called sleeping sickness;*

**Apart from the fact that the person has uncontrollable sleep at times, do you know other signs attributed to this disease?**

*P2: There are signs such as memory loss;
P4: You will see a person who was normal; at some point, their behavior changes. When asked to do one thing, they do another;
P8: You can also see a person who no longer has physical strength and who starts sleeping all the time, and even to eat, they no longer eat, only sleep;
P9: Another person becomes overly talkative and speaks nonsense;*

**Where does this disease come from and how is it transmitted to humans?**

*P6: We were taught that there is a fly that carries this disease. Once that fly bites you, you catch the disease, but I don't know the mode of transmission;
P1: Our grandparent had this disease. When they were taken to the treatment center, their accompanying caregiver was also diagnosed positive for this disease at the same treatment center. Another person was sent to care for both patients. Now, I don't know if it was because they shared food from the same plates or if it was something else;
P3: It's because they had "light blood" (i.e., were susceptible);*

**Are there ways to protect oneself from sleeping sickness?**

*P6: We only know how to avoid malaria, but for this disease, we only know that there is a fly that bites, especially in the forests around Bena Kalenga, that's where you find many of these flies, but no one has shown us how to protect ourselves;
P9: Like she said, no one has told us what to do to protect ourselves;*

**II. Perception of Health Services**

**What do you do here in the village when you feel sick? (Where do you go to find a solution?)**

*P10: We just go to the health center;
P6: When I am sick, I come to the health center, but there are other people who go directly to the pharmacy to buy products;
P3: Sometimes if someone is sick, for example, they have a headache, they come to the health center, and they are asked to buy paracetamol. After taking it, they get better. Next time they have a headache again, will they go to the health center? No, they already know what to do; they will go to the pharmacy and buy paracetamol. It's only if there is no improvement that they will consult again at the health center;
P8: There are also other illnesses that are easily treated with herbal teas, like coughs;*

**When you think, based on the signs mentioned (reiterate some signs cited by the group), that a person has sleeping sickness, what do you do to find a solution?**

*P7: For the signs of sleeping sickness, you have to start with the big hospitals, where this disease is treated;
P10: This disease affects the head a lot. If you tell the person to go to the hospital, they won't accept because they have become stubborn. For me, I will give advice to their family members so that they are taken to the health center for tests to find out what is bothering them;
P9: For me, we must take them to the health center because that's where tests are done to know what disease they are suffering from;*

**Do you know the facilities that organize or conduct screening for this disease? If yes, which ones?**

*P1: At Katanda, at Belor's place;
P4: Belor's place is at the secondary hospital; he's the one who works in the laboratory;
P5: It's always there at Belor's place;*

*How do you appreciate the services offered by the health center you frequent in the village?*

*P7: We notice that despite the building and the beds, there are cases that are not treated here. Why? Because there is no doctor. So, a transfer is made to Bibanga if we cannot cure the patient;
P8: Our health center offers good services. We are welcomed well, we are treated well, and the caregivers are kind to everyone;
P5: I add this: we can say that everything is fine, but with the exception of, uh, if I come with appendicitis, will they operate on me? They will arrange a transfer to Bibanga because there is no doctor here. That's what the health center cannot offer us;*

**How do you appreciate the distance traveled to reach the health center?**

*P10: Yes, there is a considerable distance, such as for Nkimba and Bena Kalenga. There are people who spend the whole day here at the health center because the distance doesn't allow them to return to the village and come back for the evening treatment. When they receive the morning treatment, they stay there waiting for the evening treatment before returning to the village once and for all;*

**How do you appreciate the waiting time before being seen by the health center staff?**

*P7: They do their work well. Because if you come and find them doing something else, as soon as you present a case, they stop what they were doing to attend to you;
P3: Here with us, they don't keep people waiting; it goes very quickly;*

**How do you appreciate the treatment you receive at the health center?**

*P2: We are treated well. Whenever I fall sick, if I come here, I am treated and afterwards I feel well;
P6: The treatment is good, but there are other illnesses that they cannot treat; they arrange a transfer for us to follow up treatment in Bibanga;*

**How do you appreciate the availability of the health center's nurse when you need them?**

*P7: There are four nurses. I have never seen a day when the health center was closed, meaning there was no nurse. Even on Sundays, while others go to church, one is designated to work;
P9: I see that every day there are always nurses, but at night I don't know if they are always there;*

**How do you appreciate the cost of consultation and care at the health center?**

*P8: For those who set these fees, they find it acceptable. Yes, it's good because last week I was sick, I paid 5000 francs and 500 francs for the medical record until the end of treatment;
P3: I find this price affordable;
P5: It's good for the consultation, but if you need an IV drip, you have to pay for the test and they ask you for additional fees; that's when it becomes a lot;*

**Are you aware that tests for sleeping sickness screening are free?**

*P10: We know that. Every year we even see the FOMETRO team passing by and examining people; it happens for free;*

**Is there a problem preventing the community from attending the health center for care?**

*P5: Depending on each person's means, a person may lack funds;
P3: Depending on means, even if the treatment costs 2000 or 2500 francs, you will see that there are people who lack even 500 francs to buy paracetamol;
P6: It's only money. Distance doesn't exist when you have money. If I have money, even if I am told to go to Bibanga now, I will get there because I have money;*

**What are your suggestions for improving access to healthcare services in our health area/zone?**

*P2: We want a doctor here with us so we can stop the transfers to Katanda and Bibanga. That way, if someone has a serious illness, the doctor can perform tests and start treatment;
P7: When we organized the vaccination campaign against COVID-19, they sent a doctor here. You should have seen the number of people who came just because there was a doctor present. Moreover, the fees hadn't even increased, but if they send you there (to Katanda or Bibanga), they ask for a lot of money;*

**III. Perception of Sleeping Sickness and Screening**

**How do you feel within the community if you are told that a certain person has tested positive for sleeping sickness after examinations?**

*P8: It hurts a lot, and it makes us feel regret;
P6: For me, it makes me cry; I cannot bear to see my friend or my sister suffering;
P3: It makes us uncomfortable to see a person who was with you catch a bad disease that might leave them with after-effects and disorders;*

**To what do you attribute the affliction of sleeping sickness?**

*P10: The teacher (moderator) said earlier that it comes from a fly. I cannot attribute it to anything else; it's always that fly that brings us this disease;
P1: When a person has malaria, we say it's from mosquitoes. So, we cannot attribute this disease to anything else; it's that fly that bites people;*

**Does sleeping sickness cause fear when you hear about it?**

*P4: Yes, it scares us just to see a person dozing off all the time;
P8: It causes fear because most people who have suffered from it, when we see them, there is always a place like the 9-6 (likely referring to a specific area or condition), but recently, I don't know why the disease seems to be decreasing noticeably;
P1: It causes fear because we see people who have suffered from it showing signs of mental retardation even after recovery;
P2: The name itself is not good; that's why it's scary. It's that name that causes fear;*

**Do you think you would go for screening at a health center/general referral hospital if you presented with signs suggestive of sleeping sickness?**

*P7: I would accept screening because tests must be done; if the disease is confirmed, they will treat me so I can recover;
P8: I must go get examined while it is still in the early stages. That way, I will take my treatment and the disease can go away for good without leaving me with mental disorders;
P5: I would accept going to Katanda to get it over with once and for all;
P1: I would go because the one who is sending me to do these tests knows that the solution to my illness lies where they have sent me. If I refuse, I am not a healer who can cure myself;*

**Why, according to you, are some people afraid to get screened for sleeping sickness?**

*P6: This disease will make you so stubborn that even if they come with a motorcycle to take you, you will see that the person does not accept, and afterwards they even disappear, and you start looking for them everywhere;
P2: Even where they go, you might find them fast asleep. You tell them to get on the motorcycle to go to the health center, and they tell you, "I am not sick;"
P10: It's the fear of the disease. Someone might say, "At my age, how could I have gotten this disease?"*

**Thank you.**
