# Supplementary material for: Barriers to integration of passive screening for sleeping sickness in Bibanga Health District, Democratic Republic of the Congo
Source: PLoS Negl Trop Dis. 2026 Apr 8;20(4):e0014179. doi: 10.1371/journal.pntd.0014179 (PMC13089886; doi:10.1371/journal.pntd.0014179)
Supplement: S3 File — (ZIP) [file pntd.0014179.s003.zip › S3_Verbatim transcripts/3_AS_TSHILULA/AUD.18_FG_HOMMES_TSHILUILA.docx]

**FGD WITH MEMBERS OF THE COMMUNITY OF THE BIBANGA HEALTH ZONE**

**Audio No. 18: FGD with men from the Bakwa Tshiluila Health Area**

**I. Knowledge of Sleeping Sickness**

**Are you familiar with a disease that makes the person who contracts it fall asleep uncontrollably at any time? What do you call it in your language? What are the different names for this disease and what do they mean?**

*P2: We call it sleeping sickness or somnolence sickness;*

*P3: These are the two names that exist in our language;*

*P10: Sleeping sickness because the person who suffers from it sleeps incessantly;*

*P6: The word lubungu means to always be sleeping;*

*P7: It means someone who is drowsy;*

**Apart from the fact that the person has uncontrolled sleep at times, are you aware of other signs attributed to this disease?**

*P10: The signs are numerous, but I can mention two. Firstly, sleeping sickness causes headaches, swollen lymph nodes (ganglia), physical weakness (asthenia), and lower back pain (lumbago); others can continue with the rest;*

*P8: I can also add that the person experiences mental disturbances and delirium;*

*P6: Someone takes on a strange demeanor and begins to gain weight;*

**Where does this disease come from and how is it transmitted to humans?**

*P1: Regarding sleeping sickness, I have already seen a very large fly that moves very fast; it is said that if it stings you, you catch sleeping sickness;*

*P3: We call this fly the sleeping sickness fly;*

*P9: In French, it is called the tsetse fly;*

*P5: Transmission occurs through blood; when this fly stings a sick person, it will then sting another person to inject the blood;*

*P8: To our knowledge, there are no other means of transmission; only this fly plays that role;*

**Are there ways to protect oneself from sleeping sickness?**

*P10: Yes, one can avoid the disease. Firstly, one must avoid staying where there is an overgrowth of palm trees, or where it is overgrown with dense trees and bushes;*

*P2: One must get screened every time mass screening is organized;*

*P4: For me, to avoid it, I have already seen things placed in the form of mosquito nets; if we have those and place them in overgrown areas, because overgrown areas are where these flies live, then we can avoid the disease;*

*P7: These mosquito nets are called traps; they capture the flies;*

*P1: If we eliminate all the flies, then we have also eliminated the disease, because they are the ones that transmit the disease;*

**II. Perception of Health Services**

**What do you do here in the village when you feel sick? (Where do you go to find a solution?)**

*P7: When a person falls ill, to find a solution, they go to the caregiver who is close to their home;*

*P10: It is our job—I am a community health worker (relais)—and this is what we teach the community. We tell them that when the body is unwell, even with a minor illness, one must go to the health center (CS) to be seen by a caregiver and present your situation;*

*P5: Usually, here in the village, people resort to the pharmacy for fevers or any other minor ailment. They buy paracetamol or dolaren, which they take for two to three days; that is people's habit. It is only when there is no change that they think about going to the health center;*

*P3: We in the community each act in our own way. Let's talk a bit about someone with a stomach ache; they quickly resort to a local solution, "KALAMATA"—an herb found everywhere. They will grind it, mix it with salt, and take it. This can go on for weeks or months. It is only when they see that it's too much and this solution isn't working that they go to the health center late. All this is the difficulty we, the community, bring to the nurse, sometimes with complications;*

**When you think, based on the signs mentioned (referring to some signs cited by the group), that someone has sleeping sickness, what do you do to find a solution?**

*P7: We have said here that we often arrive at the health center late. Usually, the solution is to start alone, either with the pharmacy or with herbal medicine (phytotherapy); others even go to the church. If there is no change, then we come to the health center;*

*P6: For me, one must go quickly to the health center; that is my way of seeing it;*

*P10: For the signs of sleeping sickness, the solution is to go quickly to the health center;*

**Do you know the structures that organize or carry out screening for this disease? If so, which ones?**

*P5: For us here, there is only the Katanda Secondary Hospital and the Bibanga General Referral Hospital (HGR). But since Bibanga is far from here, we often limit ourselves to Katanda.*

*How do you appreciate the services offered by the health center (CS) that you attend in the village?*

*P10: Thank you for the question. Here at our Tshiluila health center, we are facing difficulties. When I am sick, I go to the health center hoping that tests will be done to discover the cause of what I am feeling. But when samples are taken, they are sent to Katanda or Bibanga; it's a long distance. This means the service is incomplete; we do not have a laboratory technician. The second point, as we have said, is that after taking medicinal plants for two to three months without a solution, when I finally go to the health center for treatment with pain, it is found to be acute appendicitis. Instead of intervening because my life is already in danger, I am transferred to Bibanga General Referral Hospital (HGR). I think you know the distance. If we had a doctor at the health center, this problem would have been resolved on-site. Now I have to travel, and the means of transport is a motorcycle. You came by this same road and felt the difficulties yourselves with all the jolts. Even before reaching Katanda, perforation will occur followed by death. But if there were a doctor on-site, they would operate, and I would be healed. Others can also add;*

*P4: Yes, I am pleased with the intervention of Mr. Samuel Kazadi; these were the words I also had in mind. We do not have a doctor here for our care;*

*P7: We come to the health center for treatment. When we arrive, we pay for the medical file and the treatment. After paying, we are given a prescription to go and buy the products. This means that a large center like this does not have medications. And when you are given this prescription, you have to go all the way to Katanda or Kalambayi to find that product;*

**How do you appreciate the distance to travel to reach the health center (CS)?**

*P4: Yes, there is distance which constitutes a burden for the community. Take people living in Bena Kalenga, in Bena Nsumba; it takes 45 minutes or 1 hour for a non-sick person. Considering that the patient is physically weak, it is already a difficulty for the community;*

*P1: It's true that our brothers just mentioned live too far away, but there are health posts that are close to them, and that could help before arriving here;*

**How do you appreciate the waiting time before being seen by the health center (CS) staff?**

*P5: It doesn't take much time. Someone might say it takes a long time, but you have to consider when you arrived: are you alone, or did you find other patients ahead of you? And at the time you arrived, perhaps another person is in the consultation room, and you cannot see two people at once;*

**How do you appreciate the treatment you receive at the health center (CS)?**

*P6: The treatment is good; the difficulty is always what we have already said. When we come, we need healing. But when for this healing a prescription is required, that is where people say we are not treated well;*

*P2: I say that they treat well; I myself have been sick two or three times, and each time I have been treated, I was satisfied;*

*P9: I appreciate the treatment well. We had difficulties here with blood transfusions, but since we trained a nurse in transfusion, our children no longer have this problem. I say it is good;*

*How do you appreciate the availability of the nurse at the health center (CS) when you need them?*

*P5: The availability of caregivers is problematic only at night. During the day, they are there; it's only at night that there are times when you find no one;*

*P8: I say that the caregivers are available at all times at the Tshiluila health center. They have organized their hours so that there is 24-hour availability;*

*P7: For our health center in Tshiluila, there is no night when you do not find a caregiver, unless the person looking for them does not know where to find them. There is a room next door where the nurse spends the night, and if you knock on the door, they will open for you at any hour;*

**How do you appreciate the cost of consultation and care at the health center (CS)?**

*P3: The consultation only costs 500 francs (Congolese Francs). I think even the most disadvantaged can pay that, apart from other fees;*

*P4: Regarding the consultation, it is satisfactory;*

**Are you aware that tests for sleeping sickness screening are free?**

*P8: It is known that for sleeping sickness, everything is done free of charge. And when the diagnosis is confirmed, wherever you are sent, you are taken care of, even for meals. You only go there with the shirt on your back;*

*P5: We know; this message is given to the population by the mobile team each time they come to our villages for testing;*

**Is there a problem preventing the community from attending the health center (CS) for care?**

*P6: Some always say they lack means, because the consultation fee does not cover the treatment;*

*P7: The community likes free services. Even if today it is said that care costs 500 francs, they will come with 100; that's how it is;*

*P1: There are many barriers. Apart from the lack of means, I can also cite beliefs. We have religious sects that do not come to the health center, and even their wives give birth at home. Even if you exhort them, they will tell you that they can never set foot in your centers. Another barrier is the number of small-scale healers in our villages; they are very numerous and keep patients trapped in their practices. You come with a certain illness; instead of referring you, they will tell you, "I will cure your illness," and even when it becomes complicated, as long as they still see money, they will keep you with them. It is only when it becomes so complicated that they send you to Bibanga. The same goes for traditional healers;*

*P4: I add to my brother's points on barriers; there are several. It's like someone who knows that a certain plant extract can cure a certain illness; when they see their brother wanting to go to the health center, they themselves become the barrier. They will tell them, "You will waste your money unnecessarily; just take the extract from this plant." There are also fetishists in the villages who never believe in modern medicine; they also prevent those who listen to them from attending the health center;*

**What are your suggestions for improving access to healthcare services in our Health Area/Health District?**

*P1: For me, I would like all tests, including confirmation and treatment for sleeping sickness, to be done here. I tell you that if I am told, "It seems like you have this disease," I will run away. But if I am informed, "After testing, we found sleeping sickness," I will accept treatment. So give our nurse everything necessary;*

*P2: My wish is that, just as you have worked to improve care conditions, you continue so that this disease becomes like all others, with simple and easy treatment that we can take for 3 days to a week;*

*P10: We need to eliminate the flies to avoid this disease in the community; just send us the traps;*

**III. Perception of Sleeping Sickness and Screening**

**How do you feel in the community if you are told that a certain person tested positive for sleeping sickness after examinations?**

*P7: There is anxiety when it is announced that someone has sleeping sickness. People think about the old treatments, whereas things have already changed—no leave of absence, no deaths. The people treated do everything and eat everything during treatment and even after their treatment;*

*P10: Yes, there is anxiety because it is a disease with a sad history. But it is also news that gives hope because if the disease is not found, imagine how the person will suffer. Since the disease has been found, we will put an end to their suffering, and the care is free;*

*P8: When it is announced that a certain person in the village has sleeping sickness, it is very painful. But because the disease is known, we know that they will be treated and cured; that is also another feeling; we will wish them a good recovery;*

**To what do you attribute the fate of sleeping sickness? (i.e., do you think it is caused by a curse or supernatural forces?)**

*P4: It is a disease like any other; even a fever can kill someone;*

*P10: One cannot attribute the disease to any sort of curse when we know where it comes from. The problem is to clean up our environment to avoid the bite of the fly that causes this disease;*

**Does sleeping sickness cause fear when you hear about it?**

*P7: Yes, this name continues to cause fear. I say this because we see that when the mobile team comes to our villages, participation is low. Only 50 or 40% of the population come for testing; the other 60-65% are in flight. And if you ask them why, they will tell you that these people who came are the ones who transmit germs through transfer. What makes them say that? It is fear.*

*P5: The fear is still there, perpetuated by certain people who had already lost a family member under inexplicable conditions during treatment with ARSOBAL (melarsoprol). Moreover, most think the disease does not exist in the village, but that it is the mobile team that brings the disease in their injections. That is why they are afraid of the needle;*

*P3: One of the reasons is the prolonged "garage"—that is, you must no longer carry out your activities for a long time. Some people think that the disease is caught when you get close to the mobile team, so you must stay away from this team, hence the flight to the countryside, far from the village during mass screening;*

**Do you think you would go for screening at a health center/general referral hospital if you presented with signs suggestive of sleeping sickness?**

*P9: One must go because that is where we will discover if it is sleeping sickness or another disease. If it is sleeping sickness and you refuse to go, it's your problem; when the microbes multiply, that's how people develop mental disorders;*

*P7: For me, I must go because if I do not go, I will not be treated, and I will not be cured; I must go;*

*P1: I would prefer that the nurse gives me this information in a veiled way. That is, they should not mention the name because it is a name that causes fear; it might make me run away. If they simply tell me, "Go to Katanda for other tests," that is better than telling me, "It seems you have sleeping sickness. Go to Katanda for confirmation." If they say that, I will not go.*

**Why, according to you, are some people afraid to get screened for sleeping sickness?**

P5: Those who are afraid do so because of many things we have already mentioned, but we can also add the fear of the lumbar puncture.

**Thank you.**

**COMMENTARY**

Comparison between HAT before NECT and after NECT;

There is a major change according to what we learn; there are new molecules that do not require patients to observe prohibitions and do not require leave of absence;

The difference is that before, long distances were traveled for treatment; today, a patient can be treated here with us;

There is a difference between the 1st treatment and the current treatment because today a patient can be treated at home and without conditions;

The "garage" (period of inactivity) we experienced after treatment no longer exists; everything that caused fear no longer exists.
