# Supplementary material for: Barriers to integration of passive screening for sleeping sickness in Bibanga Health District, Democratic Republic of the Congo
Source: PLoS Negl Trop Dis. 2026 Apr 8;20(4):e0014179. doi: 10.1371/journal.pntd.0014179 (PMC13089886; doi:10.1371/journal.pntd.0014179)
Supplement: S3 File — (ZIP) [file pntd.0014179.s003.zip › S3_Verbatim transcripts/3_AS_TSHILULA/AUD.19_ENT_PRESICODESA_TSHILUILA.docx]

**INTERVIEW WITH OPINION LEADERS OF THE BIBANGA HEALTH ZONE**

**Audio N°19: Interview with PRESICODESA from Bakwa Tshiluila**

**I. Knowledge of Sleeping Sickness**

**What is your view on the existence of sleeping sickness in your community?**

*Sleeping sickness did exist in Tshiluila, but for the moment I cannot confirm because recently, when the mobile unit came for screening, we did not see any cases.*

**What are the signs that make you suspect sleeping sickness?**

*The signs are well-known, for example, swelling of the face (puffiness), the person sleeps at any time, persistent headaches that do not respond to treatment. The signs are numerous, and if one is examined, sleeping sickness can be diagnosed.*

**Is there a treatment for sleeping sickness? (Is the disease curable?)**

*Yes, treatment exists. I have already seen people who were treated and were cured.*

**Is your Health Center capable of performing screening for sleeping sickness?**

*There is only one place that I know of, because here in our area there is no testing for this disease; it is only available in Katanda.*

**II. Perception of Health Services**

**When you feel ill, here in the village, where do you go first to seek a solution? (Church, traditional healer, or modern medicine?)**

*It depends on the difficulties within the community. Someone can fall ill and stay at home for many days; if they lack money, they may not even manage to get to a healthcare provider quickly. A person may also use their own chemical formulas with plants. Certainly, there are also people who are kept in churches where they are being prayed for.*

**When you think, based on the signs mentioned (recall some signs cited by the group), that a person has sleeping sickness, what do you do to find a solution?**

*Our role is to awaken people’s awareness. The body does not have a spare part. We sensitize people so that if there is something wrong with the body, they must go to the nurse. It is the nurse who knows what to do, and when the situation exceeds his competence, he knows where to refer you. He sends you where you need to go for treatment. But if you go to the nurse late, and are unable to explain to the nurse what you are feeling, what will you do? We help the community. Even when a person lacks means, we facilitate their care by making a commitment with the nurse so that the person can come and pay after the treatment.*

*For signs of sleeping sickness, we refer the community to Katanda, and it is known by all that the sleeping sickness center is in Katanda, next to the secondary hospital.*

**How do you appreciate the services offered by the Health Center you attend in the village?**

*The service is good, but there is one thing I mentioned at the beginning: when a patient arrives, they must be received by the nurse. They do a rapid diagnostic test (RDT) to see if the person has malaria. But when tests like stool or urine analysis are needed, it requires that the sample be sent from here to Katanda. All this is because there is no one capable of doing this work to tell the person they have this or that other disease. However, when the results arrive, the work is done correctly.*

**How do you appreciate the distance traveled to reach the Health Center?**

*Regarding the distance, it is acceptable because people leaving from Bena Kazadi or Tshipuka to go to Kalenga will find a health post before arriving at the Health Center. So, even if someone has a high fever, they will receive emergency treatment along the way. Therefore, everyone is at a reasonable distance.*

**How do you appreciate the treatment you receive at the Health Center?**

*We receive good treatment that even attracts people from outside the area. This Health Area is appreciated for its treatment; people even come from villages in the Kalambayi Health Zone: Bajila Membela, Kanampumbi, Bashiya Kabuya. All these people come here for treatment.*

**How do you appreciate the cost of consultation and care at the Health Center?**

*For treatment such as for malaria, the cost is reasonable according to the center’s pricing schedule. However, we see in the community parents who administer malaria treatment at home on their own, without a prescription, just to reduce the fever. Now, when they notice the illness is worsening, they come quickly with the child to the center in this critical state. The child may already be anemic and then require a transfusion, and there is a prescription for supplies. At that point, you see them crying that the center is starting to ask for a lot of money. That is the difficulty with the community.*

**Are you aware that tests for sleeping sickness screening are free?**

*Yes, the tests and the treatment for sleeping sickness are free. It is known, even when the mobile unit comes, we see how they operate. We pay nothing. Even when a patient is diagnosed, everything is provided at the center for their treatment. If it is a patient that must be taken to Katanda for treatment, there they are even given food. Everything is free for sleeping sickness.*

**Is there a problem that prevents community members from attending the Health Center for care?**

*The main barrier in the community is money. Someone may lack just the small amount needed for treatment and stay at home because of it. You have to understand life in the community. You tell someone with a sick child to go for care, and they answer that they have no money; they lack just 5000 Francs and they stay at home with the sick child. That is the biggest barrier.*

*Other barriers in the community are the so-called "baminganga" (traditional healers/quacks) that are found everywhere in our villages, like Red Cross members who seem to help, but what they give does not help the community; it increases the burden, and the illness worsens. These are the cases we often receive. When you ask the question, "Where were you all this time with the child?" the answer is, "We were at home." And when asked, "Who was treating the child?" they say nothing. But after two days of treatment, when the child recovers, it is then that the testimonies start coming out of their mouths.*

*Another problem posed by the community concerns the use of syringes for injections. The community says that the baminganga in the village use a single syringe for the entire course of treatment. But here at the center, it is always "buy a syringe, buy a syringe." They complain about this even though it is for their own good, and it creates a barrier because elsewhere, for even a two-week treatment, it is the same syringe.*

*Another barrier is the elderly women in the villages who insert their fingers into children's throats to apply ash from certain plants, aiming to cure certain illnesses like fever and diarrhea. Mothers are imprisoned by these women who apply ash to children morning and evening. It is only when the condition becomes serious and the child becomes agonized that they come to the center.*

**What are your suggestions if we need to improve access to health care services in our Health Area/Health District?**

*In our Health Area, we do not have a laboratory technician for tests. We want to have one. Secondly, concerning the products prescribed to patients, you yourself saw the difficulty you faced on the road with your motorcycle, but the community makes this journey on foot to get the products from Katanda because we do not have a pharmacy here. These are the two problems that could contribute to the development of our center.*

**III. Perception of HAT Integration**

**Are you aware that the Health Center in this Health Area has integrated sleeping sickness control activities? If so, how did you get this information?**

*We are informed, but for the community, to get up and leave the house just to get screened, they only do that when they feel sick and come to the center. Now, if you dare say to a person, "Let’s go get screened for sleeping sickness," they will tell you, "Do you want to transmit sleeping sickness to me?" or "There is another problem you are looking for against me." Then they might not even accept that sleeping sickness exists.*

**How is the integration of sleeping sickness screening into this Health Center necessary for the community?**

*It is necessary for prevention, in the sense that I could be bitten by the fly in the field. To know that I am well, that my health has no problem, I need to get screened at the center for sleeping sickness. It is after that that I will know I have no problem.*

**Why, in your opinion, do some people go to get screened for sleeping sickness elsewhere rather than here?**

*It depends on each person’s behavior. There are people who, when it is announced in the village that there is screening, take their tools and go to the fields. But those who accept, they always respond and they get screened. It is not 100% who accept screening; there is a portion that exhibits this refusal behavior.*

*But there are also people who think that sleeping sickness is a mystical illness that can be transmitted to a person by occult means during screening. These people avoid getting screened in their villages because they think that this or that village sorcerer could take advantage to transmit the disease.*

**In your opinion, what should be done to improve access to sleeping sickness screening through Health Centers?**

*This requires sensitization. You mentioned that the disease had significantly decreased in 2019 and then resurged in 2020. We want the involvement of the State, like in the era of tokens, where household-by-household checks were done to discover those who had not been screened. In addition, trapping: if we are given traps to place in our villages, in places where women draw water, we will reduce the flies. But since there are no more traps in the community, the flies multiply, and there is no way to end the disease.*

**I thank you.**
