# Supplementary material for: Barriers to integration of passive screening for sleeping sickness in Bibanga Health District, Democratic Republic of the Congo
Source: PLoS Negl Trop Dis. 2026 Apr 8;20(4):e0014179. doi: 10.1371/journal.pntd.0014179 (PMC13089886; doi:10.1371/journal.pntd.0014179)
Supplement: S3 File — (ZIP) [file pntd.0014179.s003.zip › S3_Verbatim transcripts/3_AS_TSHILULA/AUD.20_ENT_IT_TSHILUILA.docx]

**INTERVIEW WITH HEALTHCARE PROVIDERS OF THE BIBINGA HEALTH District**

**Audio N°20: Interview with the IT of the TSHILUILA Health Area**

**I. Knowledge of HAT Control Strategies**

**Could you tell us about the strategies used by your Health Area to reduce the prevalence of sleeping sickness in the Bibanga Health District?**

*Yes, uh, regarding strategies, there are active screenings carried out by the Ngandajika mobile team. Apart from that, there is also environmental sanitation by the community, which involves clearing grass and such to combat the disease vector. Besides that, there is also patient management, meaning the more we manage those who are sick, the more we reduce the risk of contamination. That's it for the control methods.*

*And...*

*So at the Health Area level, for the screening process, we use the HAT RDT. When a patient arrives presenting signs like fever, headache, etc., first we screen them with the malaria diagnostic test. If it's positive, they are immediately placed on malaria treatment. If it's negative, then we proceed with the HAT RDT. If that is also positive, the patient is referred to the confirmation center because we do not confirm the disease here. Also, if a patient arrives with recent behavioral disorders, meaning less than six months, we must also test them with the HAT RDT. If positive, it's the same thing; we refer them. And even for those who tested positive for malaria RDT and were put on treatment, if their treatment does not respond or there is treatment failure after proper management with the appropriate malaria medication, and signs like fever and headache persist, we must also perform the HAT RDT. So this is how we proceed for HAT screening.*

**Since you were assigned to this Health Area, have you ever diagnosed HAT in your center? If not, why? If yes, how do you do it?**

*Yes, we do it. I take the case of a patient who came with complaints suggestive of malaria or HAT. Since here, if the malaria test is positive, we don't go directly to the HAT test. We screened him; he was positive for malaria and took the correct treatment. After this treatment, the signs persisted, so we thought to perform the HAT RDT, and it was positive. That's how we referred him to the HAT center.*

*How...*

**Have you ever encountered resistance when referring a patient for confirmation of a suspicion you made? If yes, what did you do to convince them to go to the HAT confirmation center?**

*Yes, I believe if I were to list them, it might be three or four cases. To convince them, we give them the message, we show the importance of getting diagnosed in time and receiving treatment for HAT, which is why we are referring them. So we break the resistance with counseling, but some still resist.*

*At our level, when they need to go to the management center, we issue a referral slip that we give to the family. Since the center does not have transportation means, the cost falls on the patient, which also means some cases we refer refuse due to lack of transport.*

**II. Perception of HAT Integration**

**In your opinion, when we talk about integrating sleeping sickness control activities into Basic Health Services, what do you think of?**

*When we talk about integration, it depends, but since the topic is HAT, first of all, integration happens at different levels. You can integrate just active screening, like here with us. When integration is done well, the problem is, as you mentioned, integrating the service. Integration happens, but the problem is following up on what we have integrated, ensuring the activities run normally. That's the big issue. But initially, this integration that took place in 2016, if I'm not mistaken, just after the mission, activities were almost abandoned. Facilities no longer had HAT RDTs. I believe even those who had integrated trapping, I think that was hardly practiced anymore. This means you can integrate, but if you don't sustain your activities, it can become complicated. That's all I can say there.*

*When you integrate passive screening, it means the patient themselves, feeling the signs of the disease, goes to the health facility. So they arrive, and it's up to the nurse to do the screening. That's passive screening; the patient moves to the center.*

*And...*

**Do you think integrating screening and diagnosis alone is sufficient to eliminate HAT in the Bibanga Health Zone, or are other complementary strategies needed? If not, why? If yes, which ones?**

*Yes, other activities need to be integrated. I'm saying integration alone is not enough. For instance, vector control can help eliminate the disease. The method depends; we can control through trapping or environmental sanitation. Even if we do passive screening, as long as the vector is not targeted, the disease will remain; it won't be eliminated. So we must start by eliminating the vector that transmits the disease. Simultaneously, there is screening. And even then, we had integrated screening, but some centers were targeted for confirmation. Instead of having just one CDTC, or I don't know if there are two for the Health Zone, to reduce the distance between the screening and confirmation centers, some centers were designated for diagnosis, but this was not done.*

*Another activity to add is awareness-raising and communication. The community needs to learn what HAT is first, and to do this, the RECOs should be involved in this activity. So you see, screening is needed, awareness-raising and communication are needed, vector control is needed, and confirmation centers need to be brought closer. To make myself understood, awareness-raising and communication are activities that exist but need support.*

**In your opinion, was it necessary to implement these activities precisely at this moment? Why?**

*I would say it wasn't wrong, because it was necessary to start and see what could follow. First, screening was placed in the centers, but despite this integration, difficulties still arise. So I would say that if we added the other activities I listed, it would help.*

**In what ways are the HAT control activities implemented in the Minimum Activity Package of your Health Center beneficial for you and your facility?**

*The only benefit is the realization of the activities. There is no other benefit like remuneration or anything else. These are activities integrated like all the others. It helps the community more, but for the health worker, it just, uh, helps develop their knowledge of sleeping sickness and also master certain practices related to the activity. Regarding the facility, it just increases the package, the number of packages the facility realizes.*

**In what ways are the HAT control activities implemented in the Minimum Activity Package of your Health Center beneficial for the community of your Health Area?**

*Regarding the community, it's beneficial because it helps reduce the disease rate in the community. When we screen more, we are reducing the number of cases.*

**What do you think about the time you spend screening a suspected HAT case in your Health Center compared to routine daily activities? Does this time represent a loss of income for you and your facility?**

*Well, as I said, the more activities you add, the more it's an added workload. It just requires organization, how to plan activities. Nevertheless, it's an additional workload.*

*Well, here I would say it's not a loss of income. I say it's just a planning issue. Since it's an activity of the Minimum Activity Package like the others, we plan for it. There aren't specific days for it, no, no. It's an activity like what we do for malaria. Indeed, as I said earlier, it's an additional workload, so it requires effort to harmonize all the activities we have. It requires a bit of effort. Sometimes when we have many activities to carry out, we might not do others well, which sometimes requires... we need to review the staff numbers because the more activities there are, the more we need to review the staff and truly consider the involvement of all members. So that's all.*

*If...*

**III. Perception of HAT Elimination**

**In your opinion, what do you think of when we talk about HAT elimination?**

*When we talk about HAT elimination, well, I understand that we will reach a point where there are no more cases. We screen everyone and they are negative. It should take time to declare the disease eliminated. The notion of time is that we reach a point where we have zero cases of the disease, uh, trypanosomiasis.*

**Do you think HAT elimination is an urgent matter in the Bibanga Health District?**

*I would say it is urgent because this disease is also decimating the population. It really is urgent.*

**In your opinion, what is the most effective way to eliminate HAT? Why is this method more effective than others?**

*I can summarize the methods into three. We need to focus on prevention, treatment, and screening. All three are necessary; I would even say they are complementary. We cannot remove any one of them. We must work with all three, but focus more on prevention. Because now cases are decreasing somewhat, and we still wonder when there were many cases. I take the example of this Health Area when there were still so many. The mobile team would come, and there were over 20 cases, etc. There were really resources. Now with the decrease, we find that activities are almost abandoned, I can say that, prevention activities. There is almost treatment and screening, but the means for vector control – I see that it doesn't exist, yet it is the means that can lead us to eliminate the disease. We must support this vector control method much more, trapping. Uh, also communication, as I said, awareness-raising, we must support that too. In awareness-raising, we must tell the community to eliminate the breeding sites of these flies, to have knowledge of the disease, to go for screening when feeling sick. This will lead us to elimination. As you are not unaware, sleeping sickness is a disease still surrounded by stigma in the community. Sometimes it's attributed to witchcraft, to curses, and if someone has it, they think it's a spell cast by evil people. We must work with the community to remove all these misunderstandings they have about the disease. You see that this requires supported awareness-raising.*

**What do you do at your level to make HAT elimination a reality by the 2030 horizon?**

*At our level, we have passive screening and also awareness-raising, but it's not really of good quality. I mean awareness-raising that is not carried out as it should be. I'll take the example that compared to other activities, when there is a supported awareness-raising activity, you'll see that this activity goes somewhat differently than one that is not supported. What does that mean? The relays who go into the field take this as an indicator; we evaluate how the activity goes. This can lead them to intensify communications, messages, home visits, etc. So that's where I say it happens, but the way it unfolds is not as intended, yes.*

**IV. Community Accessibility to HAT Screening Services**

**What do you think about the attendance rate at the Health Center by the community?**

*Well, the attendance, I can say, is good but not very good. There are some obstacles. The Health Area covers a somewhat large area; some communities come from quite far, up to about 16 km away. So geographical coverage already poses a problem. Apart from that, there are a number of health posts that have been set up in a truly anarchic manner, which do not transmit data. So we have no control over them; we have no reports on what happens there. This is also among the obstacles causing us to lose some patients. Apart from that, there is also, I would say, the perception in the community because we have Baba sects here; they do not consult the Health Center. When they are sick, they treat themselves with something. Additionally, there are financial barriers. So someone may be sick but thinks, maybe they went last time with money and didn't settle the bill; they think if I go back with the debt from last time, they decide to stay home and sometimes even die because they lack money.*

**What do you think about the availability of HAT screening in your Health Center?**

*Availability is a very serious problem. I remember when the study was still ongoing, the supply chain was truly respected. Since the study ended, it's like I told you before, activities were almost grounded. We were no longer supplied with inputs; sometimes even reports were not requested. On our side, we continued working; sometimes we would put zero if the RDT was not performed. I even remember in 2020 or 2021, the coordinator of the PNTHA came to Bibanga; we spoke with her during the monitoring meeting. She promised that the RDTs would come, but it still took time. That means there were still no RDTs in the health District.*

**How do patients perceive a positive HAT RDT result when they came for consultation suspected of malaria?**

*It's more complicated for patients who are not, uh, who are not severe. For a patient who is somewhat well, who walks, who has strength, they accept it with difficulty, really with difficulty.*

**What do you do in a situation with a person who refuses to believe the result of a positive HAT RDT after examination?**

*(....)*

*To convince them, we always tell the patient that we have screened them, and now they need to go for confirmation of what we did at the Health Center. Well, some accept. I say those who don't, there's even one who maybe came with those signs; you release them, but the next day you find them already seeking care elsewhere. They say I went there, they told me this and that, that it's sleeping sickness, but I don't feel anything, I feel fine...*

**What prevents the community of this Health Area from accessing the care offered by your Health Center?**

*Well, the stock-outs of medicines. Since we experienced medicine stock-outs, facilities have started to fend for themselves. They always want to gain something from the patients. That's one of the barriers.*

*I also think that supporting the facility with tracer medicines and also supporting healthcare providers' remuneration, because if providers are properly taken care of, I think it won't shock them much. Maybe we can just tell a patient who doesn't have means: if you don't have money, just come; when you have it, you'll come and pay.*

*You see, when there are medicines, the patient only pays for the consultation; we give them the medicines. Now, when the patient comes, you consult them; they pay us the consultation fee; they themselves go pay for the medicines on the prescription; you see, it's somewhat double.*

*The second cause is the lack of remuneration for health workers by the State. You see, if they are hungry, they will try to profit something from the patient. But if they are well paid, they won't do that. This is a barrier that blocks access to care.*

**What do you suggest to improve the utilization of the Health Center by the communities?**

*I will insist even more on awareness-raising, because anything we can do for the community without them is truly working against them. So we must truly inform the community about all the activities we are carrying out. But if activities happen at the Health Center level and the community is not informed, this activity will inevitably fail. So we must put more resources into awareness-raising with our RECOs. We must fight against vectors. We must make screening inputs available. And, as far as possible, if transportation means could be provided for those patients who have to leave the screening center to go to the confirmation center, this would certainly help eliminate sleeping sickness.*

**Thank you.**
