# Supplementary material for: Barriers to integration of passive screening for sleeping sickness in Bibanga Health District, Democratic Republic of the Congo
Source: PLoS Negl Trop Dis. 2026 Apr 8;20(4):e0014179. doi: 10.1371/journal.pntd.0014179 (PMC13089886; doi:10.1371/journal.pntd.0014179)
Supplement: S4 File — (ZIP) [file pntd.0014179.s004.zip › S4_Verbatim transcripts/4_AS_CIBILA/AUD.23_FG_HOMMES_CIBILA.docx]

**FGD WITH MEMBERS OF THE COMMUNITY OF THE BIBANGA HEALTH DISTRICT**

**Audio N°23: FGD with men from the Cibila Health Area**

**I. Knowledge of Sleeping Sickness**

**Do you know a disease that causes the person who catches it to fall asleep at any time and uncontrollably? What do you call it in your language? What are the different names of this disease and what do they mean?**

*It is a disease that afflicts people here, but recently its prevalence has decreased in the population. It is sleeping sickness. It is the disease of drowsiness.*

**Apart from the fact that a person experiences uncontrollable sleep at times, do you know of other signs attributed to this disease?**

*What we know here in Tshitolo is seeing a person, like we are here talking, and they are doing this (gesturing)... they are like that... that's what we know. We also see a person who, if tied with ropes and manages to free themselves, even someone like us here wouldn't be able to restrain them. When they are brought to the health center, they can be diagnosed with sleeping sickness. That is to say, they have behavioral disorders; even if we are talking like this, they are off-topic. There are also signs such as paralysis of the limbs on one side. You also find swollen lymph nodes in the neck. There are still others; you see a person who starts to gain weight and sleep at any time.*

**Where does this disease come from and how is it transmitted to humans?**

*In the past, we set traps everywhere. It was said that the disease comes from the tsetse fly in the forest. People who farm in the forest, this fly can bite them, and they will get this sleeping sickness. When the tsetse fly leaves the forest, it comes and bites a person, and the person catches this disease. The disease is caused by the fluid that the tsetse fly injects into the body of the person who is bitten.*

**Are there ways to protect oneself from sleeping sickness?**

*Since sleeping sickness is also caused by mosquitoes, to be protected you must use a mosquito net or avoid walking in places where these insects are present, such as along rivers because the tsetse fly often comes from water, or in the forest. To truly avoid sleeping sickness or the tsetse fly, you need to clean the environment to avoid anything that causes clutter: flies, mosquitoes, and others. Now, for fields and market gardens, it is you health people who can help us with traps. They are not sold in the market, but recently we don't see them anymore. I have heard about pigs; when they sleep during the day, there are insects that bite them, and when they come to bite a human, they can also catch sleeping sickness. So, spending the night with animals in the same environment can also facilitate the transmission of sleeping sickness. To do better, we should get vaccinated against this disease.*

**II. Perception of Health Services**

**What do you do here in the village when you feel sick? (Where do you go to find a solution?)**

*We come to the health center or the dispensary.* *It is always at the health center.* *We go to the health center, have tests done to find out what the illness is. It is through the tests that the nurse will tell you if you have malaria, worms, or other diseases.* *It depends on the person. Some may start with the church, others with itinerant healers. They have products; if they give me some, I take them first. If I find there's no change, I come to the health center because there they have tests. If the illness is discovered, they will treat me or transfer me elsewhere. Here in Cibila, if I fall sick and don't even have 50 Fc to buy Novalgine, I take different plants we know. I boil them and drink, or I cover myself to inhale the steam. That's what we do sometimes, due to lack of means.* *When I have headaches or fever, the majority here, no one starts by going to the health center. It's always pharmacies; we buy products and start treatment at home. We take them on the first day, the second day. Sometimes it's only when someone comes to alert you, saying you've been taking the products for some time and don't see any improvement. There are also all the plants with a strong odor that we use as a solution to treat various diseases. We can take them for up to a week or more. It's only when there's no change that we rush to the health center. Now, at the health center, when you arrive and you don't have money, they tell you to pay for the consultation form. That becomes a problem. Since I wasn't treated, I always start with the money. Some even get angry and return home to buy products from the pharmacy.*

**When you think, based on the signs mentioned (mention some signs cited by the group), that a person has sleeping sickness, what do you do to find a solution?**

*If the signs are already present, we send such people to FEMETRO because that's where sleeping sickness is treated here in Tshitolo. We will send them to FOMETRO so they can be examined because the equipment to detect sleeping sickness is only available there.*

**Do you know the structures that organize or carry out screening for this disease? If so, which ones?**

*It is at our Cibila health center; that's where there are tests that detect sleeping sickness. The FOMETRO center in Tshibile is where everything is done for sleeping sickness.*

**How do you appreciate the services offered by the health center you attend in the village?**

*At our center, everything goes well. As soon as you arrive, you pay for the form, and treatment begins. I don't want to comment, but I will speak about my case. I had a motorcycle accident. The way I was received and the care I received, I would say they offer good care services. We were born here, we know our center well. There is a good welcome, but one thing is that for diseases they cannot handle, when they give you a referral letter, you don't have the means to travel, and sometimes people die on the way.*

**How do you appreciate the distance you have to cover to reach the health center?**

*It is very difficult. For us in Bakwa Njiba, to reach Cibila, we have to cross two rivers. After rain, we might not cross for a week. How can we manage with a patient who is my size if they cannot walk alone? To reach Kasonga, it's the same with rivers and a distance of a two to three-hour walk. Take the case of Mulenga, which requires a full day's walk. It is serious if we don't find solutions because even with the health posts, if there is a case needing transfusion, they have to refer to Cibila. With this distance, won't the child die on the way?*

**How do you appreciate the waiting time before being seen by the health center staff?**

*For someone who has already arrived at the center, I have never seen anyone complain, even at night. Since there is staff on duty, as soon as you present yourself, you are seen. At night, sleep can be deep, but when people shout to wake the nurse, sometimes I wake him with my phone since I have their numbers. I call. It's easy to wake someone with a phone. Sometimes people are mistaken and come knock on my door, and I direct them. There is no day when the center is without a nurse. Everyone has their own understanding. I don't know if when you arrive at the center, they will immediately give you an injection and start treatment without knowing what you are suffering from. The nurse might have you wait just for a short rest period before listening to you, as you have just arrived. But some people are not understanding; they find this a waste of time and complain about it.*

**How do you appreciate the treatment you receive at the health center?**

*For us, everything starts with tests, which means the treatment we receive is appropriate for the complaint and the illness. If you have a stomach ache, it's after the tests that they tell you what they found before giving you treatment. So, they treat us very well. They treat us, they do, but the difficulty is with the products and equipment. Treatment requires products, but you can arrive at night, and there are no products. At that point, we don't know what to do for the treatment you came to the center for. That is the difficulty.*

**How do you appreciate the availability of the health center nurse when you need them?**

*The nurses are always present, and if they are not at the center, they are not far from it. Sometimes we receive patients coming from very far away who knock on our door or the door of our chief. We inform the nurse, who sees them. Rarely on Sunday, the nurse might leave for a short time for prayer, but it's for a short while. Most of the time, there is always one or two nurses present.*

**How do you appreciate the cost of consultation and care at the health center?**

*Everyone does not have the same standard of living. Some have means, others do not. That's the difficulty when people come for care with the same fee. One person cannot manage to pay, while another pays as if this money was set aside just for that. I answer as the other just said. I might have means. When I come to the center and they ask me to pay, I find the fee reasonable. Then another person comes who is unable to pay the same price for care? That means there is no affordable cost for everyone; it depends on each person's means. I understood your question as speaking about consultation fees, not for all illnesses. With the 1000 Fc they ask for the consultation form, I think it is affordable for everyone. Other fees depend on the severity of the illness from one person to another.*

**Are you aware that sleeping sickness screening tests are free?**

*We are aware of that. It has been a very long time since sleeping sickness care has always been free; we do not pay.*

**Are there any problems that prevent the community from attending the health center for care?**

*Yes, the social environment. Someone can be sick. Their drinking buddies tell them, "Malaria doesn't resist alcohol, just have a drink and you'll see." Influenced by their entourage, they stay home for three days intoxicating themselves with alcohol. Lack of means is a major factor preventing attendance. The example just given about alcohol is known to everyone here in the village. Even if I lack 100 Fc to buy a tablet, I can still drink alcohol without paying anything. I just need to go to where my drinking buddy is, join him, and share what little he has. But it's not the same with hospital care, where you have to start by paying for the form. I will say this: there are people in the village who cannot even pawn a shirt for care. I would say they don't even have 100 Fc in the house to pay for a paracetamol tablet. This situation prevents them from coming to the center when sick. Another thing is bad company. Someone has small means to go to the center, but their company creates a barrier, like the talk about alcohol. They have the flu. Their friend says, "This is a chicken disease, just have a glass of hot alcohol morning and evening, you'll see." Since they have a bit of means, they do this morning and evening. After 3 to 4 days, they realize there's no change, yet they have spent more on alcohol than they could have spent on their healing by going to the center. Another is belief. A minor illness starts, and they look for the sorcerer behind the disease. Leaving the solution elsewhere, they turn to customs, slaughter an animal to sacrifice to ancestors, while the disease is curable with simple treatment. Another obstacle is itinerant healers. You find unqualified people who travel from village to village with their dispensary, pharmacy, everything in a bag, performing circumcisions, administering treatments. All this distracts the communities. Instead of going to the center, someone will say, "I'll wait for the healer who is supposed to pass by on such a day."*

**What are your suggestions if we need to improve access to health care services in our Health Area/Health District?**

*Our center lacks products; the pharmacy is empty. When a patient is brought in, the difficulty is not the money but rather the products missing from the pharmacy. Because sometimes we have to leave the patient to go where we can find the product. Here, we go as far as Bakwa Bowa; you see the distance. Our center is too small. Its capacity is exceeded. There isn't even a place to spend the night if we have a patient to watch over. For me, it's access which is difficult for the entire community of the Health Area. This could be improved by increasing the number of health posts. Since there are six groupings in the sector, if we place even one health post in each grouping, that would be good because there are natural barriers that mean even a person with means and the will to come to the center cannot always make it, like during the rainy season when it's not easy to travel when the rivers are full.*

**III. Perception of Sleeping Sickness and Screening**

**How do you feel within the community if you are told that a certain person tested positive for sleeping sickness after the exams?**

*It's sad news because we know very well the damage caused by this disease in our villages. People died here like animals. If today we talk about this disease, it's as if we are still living in that era. It causes worry, but my feeling is to see them get treated and be cured. When one of us is sick, this disease is an epidemic. We are afraid it will spread in the community. But when the person starts their treatment, we feel joy because our brother will be saved, and we will be spared from this disease.*

**To what do you attribute the fate of sleeping sickness?**

*All diseases have different levels. There is the tsetse fly that transmits the disease, as we said here. But there are also sorcerers who cause the disease and infiltrate it into the community. The disease caused by the tsetse fly is treated and curable, but the one with a mystical source is not curable and often leads to death. Sleeping sickness cannot be attributed to sorcery. It is a disease like any other that can be cured. However, all diseases can kill; it's not just sorcery. Sleeping sickness is like other diseases. When does it become dangerous? It is when after treatment they give discharge, but later when you return, the disease is still there. You return again, and the disease is still there. It is at that point that we see that impure spirits are involved in this disease.*

**Does sleeping sickness cause fear when you hear about it?**

*Yes, it causes fear. We even see some people flee from the mobile team's screenings, saying, "I still have my things that I need to finish. If they catch me with the disease there, what will I do?". It causes fear because people think it's a mystical disease. You can catch it just because you presented yourself where they work, especially if you agree to be examined. People say it is those who come to screen the community who bring the disease in their equipment and inject it into us. That is why they are afraid.*

**Do you think you would go for screening at a health center/general referral hospital when you present signs suggestive of sleeping sickness?**

*Yes, I must accept because when I come to the center, it's to be treated. If the disease I came to the center for is not discovered, I will continue to suffer. When the nurse refers me to another facility for tests, it's because he is limited in terms of testing capabilities. I must go for the tests because it is the only way to discover the disease that is making me suffer and to get proper treatment.*

*Why, in your opinion, are some people afraid to get screened for sleeping sickness?*

**Thank you.**
