# Supplementary material for: Barriers to integration of passive screening for sleeping sickness in Bibanga Health District, Democratic Republic of the Congo
Source: PLoS Negl Trop Dis. 2026 Apr 8;20(4):e0014179. doi: 10.1371/journal.pntd.0014179 (PMC13089886; doi:10.1371/journal.pntd.0014179)
Supplement: S4 File — (ZIP) [file pntd.0014179.s004.zip › S4_Verbatim transcripts/4_AS_CIBILA/AUD.25_ENT_AC_THA_CIBILA.docx]

**Interview with Former HAT Patients in the Bibanga Health District**

**Audio N°25: Interview with a Former HAT Patient from the Cibila Health Area**

**I. Perception of Sleeping Sickness and Screening**

**What do you think about the existence of sleeping sickness in your community?***Yes, the disease exists. When we go towards Lubilanji, that is where there are many tsetse flies. But since the mobile teams started conducting examinations here, there has been a decrease in the number of cases.*

*In your opinion, what is the origin of sleeping sickness?
The disease comes from bushy and dark places, where trees block the sun's rays; at those times, tsetse flies take the opportunity to bite people.*

*Is sleeping sickness dangerous for those who contract it?
It is dangerous because a person can die on the spot. The microbes are different. There are microbes of a red color; a single one can kill a person directly. Whereas with microbes of a black color, a person can resist even with 350 or 360. If the hospital is nearby, someone can survive if tests are done. So, the danger is death.*

**Was it easy to suspect sleeping sickness at home, as is done for other diseases like malaria, which you might suspect yourself even without consulting care services at the health center or general referral hospital?***It all depends on the training. The nurses who studied FOMETRO are different from those in the health centers. Those from FOMETRO have learned the signs to recognize sleeping sickness. But those in the centers, who do not know sleeping sickness, when they run tests, they tell us they found malaria.*

**What were the motivations that prompted you to get screened for HAT at a health facility?**
*What prompted me were the signs announced on the radio: severe headaches, fever throughout the body all night long, and in the morning, heat at the fontanelle. These are the signs that prompted me to consult the center for examinations.*

**Could you describe the pathway you followed from when you first felt ill until the moment sleeping sickness was diagnosed? How much time approximately passed before you were diagnosed?**
*When I felt these signs, I went directly to the center and had not taken anything else. However, I had spent nearly a month without working. Then I came to the Cibila center. KABE conducted the examinations and found the sleeping sickness microbe.*

**What was your feeling when you were told you had sleeping sickness?***When they told me this, my feeling was to quickly find a transport vehicle to go to Mbuji-Mayi for treatment. My reaction was appropriate to what I had already learned on the radio.*

**In your opinion, why are some people afraid to get screened for sleeping sickness?***The fear comes when they see the team coming to do the screenings. Many people think it is this team that brings the disease to inject us with it, and they refuse to be examined.*

**II. Perception of Health Services**

**When you feel sick, where do you go first to find a solution? (Church, traditional healer, or modern medicine?)**
*I am a pastor. When I am sick, I start by praying. But if the case requires seeing a nurse, I pray and then go to the center for examinations, especially in the case of a high fever.*

**Where were you screened and treated for sleeping sickness?***I was diagnosed by KABE at the FOMETRO center. He found a single microbe. Then I was referred to Mbuji-Mayi, to the sleeping sickness center; it was there that I received the treatment.*

**Before being diagnosed and treated for sleeping sickness, where did you go first for care? (Please elaborate: why did you leave that place for another?)**
*I first went to MAKASI. They did examinations using blood taken from the vein but did not see the microbe. They simply told me they hadn't seen the microbe, but that my blood was not good, and I needed to go to KABE for further examinations. It was he who saw the microbe that was in my body with his equipment.*

**Is there a problem that sometimes prevents you (or members of your household) from attending the health center for care?**
*Firstly, it is doubt. Many people have doubts when they are told to go for care. Secondly, it is lack of financial means, which is an obstacle to attending the center.*

**What are your suggestions if we are to improve access to healthcare services in our Health Area/Health District?**
*The number of FOMETRO teams should be increased so that they can start moving around everywhere. There are many sick people in the villages, but they are afraid to come to the center.*
