# Supplementary material for: Barriers to integration of passive screening for sleeping sickness in Bibanga Health District, Democratic Republic of the Congo
Source: PLoS Negl Trop Dis. 2026 Apr 8;20(4):e0014179. doi: 10.1371/journal.pntd.0014179 (PMC13089886; doi:10.1371/journal.pntd.0014179)
Supplement: S4 File — (ZIP) [file pntd.0014179.s004.zip › S4_Verbatim transcripts/4_AS_CIBILA/AUD.31_ENT_IT_CIBILA.docx]

**INTERVIEW WITH HEALTHCARE PROVIDERS OF THE BIBINGA HEALTH DISTRICT**

**Audio N°31: Interview with the IT (Nurse) of the CIBILA Health Area**

**I. Knowledge of HAT Control Strategies**

**Could you tell us about the strategies used by your Health Area to reduce the prevalence of sleeping sickness in the Bibanga Health District?**

*Yes, we have several strategies. First, there is trapping within the community with the RECOs (community relays), then screening with the mobile team. At the health center, we screen any case that comes for curative consultation. So, every case that comes to the center must be screened for HAT. Secondly, the community takes charge through trapping and through the monthly passage of the mobile team. You see, cases have decreased.*

**Question (implied: How do you proceed with screening?)**

*So, once we receive a patient, they must go to the laboratory. Based on their complaints, if there are cases of headaches, and since we are in an endemic zone, we must perform an HAT examination for any patient because we have the HAT RDT. Or we do this if we see that malaria is negative; then we must perform HAT. If it is positive, we must proceed to other examinations. The community and health area providers are the personnel involved in screening. Afterwards, coordination comes to support us as well.*

**Since you were assigned to this Health Area, have you ever diagnosed HAT in your center? If not, why? If yes, how do you do it?**

*Several times. As a screening center, we even manage patients. We screen them very often.*

**How? (Question likely implied from previous answer, but not fully transcribed)**

**Have you ever encountered resistance when referring a suspected case for confirmation? If yes, what did you do to convince them to go to the HAT confirmation center?**

*So, we have RECOs. Once screened, we must follow the patient to their home to explain the advantages and disadvantages. When you are taken care of while the disease is still at an early stage, there are many advantages to being cured. But if you refuse treatment, if you are stubborn, when you take the treatment later with delay, there are many complications that can affect you. That's why we have RECOs. We take the addresses of the patients we manage. If there is a case that we can manage at our level, we do so. If there is a case to refer to the DIPUMBA coordination, we refer. It's always like this, it's what we do here. Our center does both: screening and management.*

**II. Perception of HAT Integration**

**In your opinion, when we talk about integrating sleeping sickness control activities into Basic Health Services (SSP), what do you think about?**

*So, when we talk about integration of control, it's to definitively eliminate the disease before the planned date. That's why at our level, we fight, we do everything. We have even involved the community so that there is early screening. At their level, if they see a patient complaining of headaches, there are signs we have even shown to the community, if there is just one sign, bring the person to the health center for early screening. That's what we do here.*

**What (about the challenges or perceptions?) (Question incomplete)**

*It's well-known, because a destitute patient, when you tell them to go to the center, they will think they will ask me for money. What can be done? That's why at our level, we register all cases that come. Whether they have means or not, we will identify all that. First, send the patient, you will discuss with him. As we do, like for tuberculosis here, we did it for free. And then, when there are already signs, well, the RDT is free, it won't cause a problem.*

**Do you think integrating only screening and diagnosis is enough to eliminate HAT in the Bibanga Health Zone, or are other complementary strategies needed? If not, why? If yes, which ones?**

*So, for me, we have almost all the means, except what I can ask from the partners is to encourage the providers first, because they do it somewhat voluntarily. That's what discourages them. Secondly, the community also needs encouragement because the RECOs receive nothing, that's why they are disgusted. But if we need to add other strategies to screening, we need trapping first. Because in the community, we need traps where there are flies. We must eliminate them all. Then you will see that sleeping sickness will decrease further and we will eradicate the disease. But if there are no traps in the community, how will we reduce the flies? That's why we also need to increase the number of traps in the community and then encourage the RECOs, and even the providers who work. That's it.*

**In your opinion, was it necessary to implement these activities precisely at this time? Why?**

*They are right, because there is a distance to the management centers. But if it was integrated into all health centers, even if a case comes from far away, even if it's 5km, we can already identify it. The team is right because there are centers far from our structures that manage patients. Why? If there is a case at a long distance, they can be screened with HAT RDTs, and then they can be referred to the screening center for further examinations.*

**How are the HAT control activities implemented in the Minimum Activity Package (PMA) of your health center beneficial for you and your structure?**

*It's beneficial because in that time, there were many deaths, there were many people with mental disorders. Since we established this system, I see that I don't find deaths, there are no patients with mental disorders. And before, once screened, you had to send someone to Ngandajika, to Bibanga, or even to Dipumba. But the person could lack transport means. Since it's done on site, it's good. We have established this well. There aren't many expenses.*

**How are the control activities implemented in the PMA of your health center beneficial for the community of your Health Area?**

*It's beneficial for the community and even for the health center, because a case screened at the health center who doesn't have the means to go to Dipumba, that case can still transmit the disease to other healthy people. That's the continuity. That's why it's quite good because management is on site. That's the advantage.*

**What do you think about the time you spend screening a suspected HAT case in your health center compared to routine daily activities? Does this time constitute a loss of income for you and your structure?**

*No, when we work, it's for the community. You see the disadvantages that existed in that time. That is, a patient we screen, there are signs, but we want to send them to the CDTC (Treatment and Control Center), and the person flees, they say no, it's trypanosomiasis, I don't want to go. But when we are here, he doesn't know it's trypanosomiasis. He is there. It's us who screen him and see that it's such a sign, it's trypanosomiasis, tuberculosis, that's it. But when it was separate, he could say, "Ah, they are sending me to an office there, that's for trypanosomiasis, no I can't go, I don't have that disease." That's why now we encompass everything. Now it's already advantageous.*

*For us, it's not a loss of income because the staff is paid by the state. There are opportunities we find in the health center. But what we can say is that the partner who manages the people could manage everyone, then it would be good continuity. Despite all that, the state is there, it pays us, we have bonuses. Sometimes others have salaries. That doesn't mean it's a loss of income for us.*

**III. Perception of HAT Elimination**

**In your opinion, what do you think about when we talk about HAT elimination?**

*When we talk about elimination, it means we cannot find even a single case in the community. That's elimination. But if we find that there is a case somewhere, even in one year we find that there is a case, then we have not yet eliminated it, because one person can transmit to another. That's why we must eliminate completely. We must eliminate, meaning no cases, not even one case in the community. That is elimination.*

**Do you think HAT elimination is an urgent matter in the Bibanga Health District?**

*Yes, it is urgent because we live in areas where there were many cases, rivers, mountains, forests. But we want to eradicate all that because if the neighbor is sick, think that even your family or yourself... Why? We must eradicate this.*

**What, in your opinion, is the most effective way to eliminate HAT? Why is this method more effective than others?**

*It's screening in the community. That is the very basis. That's where the patients come from, meaning in the community. If we screen early in the community, we will have no problems.*

**What do you do at your level to make HAT elimination a reality by the 2030 horizon?**

*So, the community sends us cases of illness, cases of patients to the health center. We, in turn, screen because for every entry, every patient, we must do the HAT RDT, especially where there are complaints, especially where there are cases of malaria negativity. We must always do the HAT RDT. If it's positive, we must continue the examination to see if it's a case of trypanosomiasis or another pathology.*

**IV. Accessibility of Communities to HAT Screening Services**

**What do you think about the attendance rate at the health center by the community?**

*There is good attendance, but we have secondary structures, health posts, that also facilitate screening for us. We have five health posts in the Health Area because the center is far from certain Health Areas (CACs). We have health posts that also facilitate receiving patients. If in the post they find a case to refer, they send them to us. That facilitates things quite well.*

*But depending on distances, for example when we go beyond Kasongo, if there is a case, even a suspicion, we ask that they be sent to Bakwa Mulumba because at Bakwa Mulumba there is a management center. That's it, because Bakwa Mulumba and a case from Miombo, for example, are a short distance apart. Instead of going to Tshibila, they go to Bakwa Mulumba. That's what we do here.*

**What do you think about the availability of HAT screening in your health center?**

*Yes, there are sometimes shortages of RDTs, which causes problems. I say sometimes, but not very often. We are supplied by the coordination and sometimes by the zone. But that's the problem we have because when there is a shortage, you see that cases are missed. That's why we do targeted screening for the moment. It's not like we did in the past where every person who came we should screen. But now we see. We first do the malaria RDT. If it's negative, we think about HAT. If there are repeated headaches, if there is... always like that, or if we manage a patient today and after three days there is no change, if there is no change we think about HAT. It's already targeted screening, not like before, due to the lack of RDTs. Because if we could screen all patients we manage, we could run out of stock. That's why we do targeted screening.*

**How do patients perceive a positive HAT RDT result when they came for consultation suspecting malaria?**

*Yes, when we have done the tests, for example the HAT RDT which is positive, we explain to the patient: "You see the test we did shows that it could be sleeping sickness. It's not that you are sick, but we need to continue with the examinations," as we continue with other examinations. Once screened, that's when we will indicate that the person is sick.*

**What do you do when faced with a situation where a person refuses to believe the result of a positive HAT RDT after examination?**

*These are cases we encounter very often. There are many people who refuse. "No, I don't have that disease, it's diabolical. I don't have that disease." Especially people from Malembe who pray in spiritualist churches. Now, once... there was a case at Bena Kalenda, there was a case that was screened by the mobile team from coordination. That was three years ago. We even involved the Territorial Administrator (AT). That's why the person was placed in the center for management. Otherwise, by all means, he had refused. The team even went through there. Dieudo, whom you know, went through there. No way. Now we involved the groupement chief. It was only following the involvement of the Territorial Administrator that the patient came to be treated at the center. It's recent, not even a month ago. That's the problem we find here. Especially management. Secondly, the ironic belief of saying these are diabolical diseases. That's all.*

**How do you assess the acceptability of going to a referral structure for confirmatory diagnosis by a suspect with a positive RDT?**

*The one who accepts easily is the one who has suffered a lot. But a new case like that cannot accept. They stay at home and say "I will go," but they can take months and months. To convince them, you need to involve the family and certain leaders. It's difficult.*

**What prevents the community of this Health Area from accessing the care offered by your health center?**

*So, firstly the financial problem because once you arrive at the center, you need to buy the consultation card and all that. It always causes problems. That's why people don't come. If you screen and find that you don't have trypanosomiasis, you have malaria, pay as much. That's what causes the issue, especially the financial means. People don't have the means to get treated and get screened.*

*Other barriers are... well, people like to consult diviners, spiritualists, especially people from certain churches, for any illness. "Oh, these are diabolical diseases. Instead of going to the center, let's go see this prophet." That's what causes problems. It's a real problem of belief. They say that disease doesn't exist, it's not a real disease. It's like HIV, any chronic disease, the community doesn't accept it easily.*

*Another everyday issue is the presence of small pharmacists in the villages and charlatans who block the community. Even when there is a trypanosomiasis problem, they say it's malaria. "Come, I'll give you the product." Then when it's not successful, they say it's typhoid fever, forgetting that we are in an epidemic zone for trypanosomiasis.*

*These are the problems we have here: (1) beliefs, (2) charlatans and even pharmacies that are scattered everywhere. Even a woman who doesn't know how to read or write, she still sells products. "You have fever, you have headaches... Take Relief." All these are problems. Afterwards, the person will come to the center after a long period of suffering when someone tells them, "No, this is another disease, go see the healer." In the end, when the person is already exhausted and has been stripped of everything, they have spent what they had. That's it.*

**What do you suggest to improve the use of the health center by communities?**

*In the past, we suffered because back then the community didn't even know what the signs of trypanosomiasis were. But at the moment I'm speaking to you, if a patient comes, they say, "Dear nurse, I would first like to have tests for trypanosomiasis." They themselves request it.*

*They now know that the disease is curable and that treatment is free. That's why communities are already engaging. For usage, there is no problem here. Patients come themselves and also request to have all the tests.*

*But to improve access, there needs to be a flat rate. We don't have medicine. The state should support us. That's the big problem. Because the community suffers, they don't have money. Now, when we ourselves are not paid, you see there is a problem. There is no medicine, first to buy it, and then the patient who comes is poor, they don't have means. I myself, the staff you find here, I don't have means. You see that's the suffering. We need to have products, we need to be bonused and paid by the state, the government. You will see that everything will go well.*
