# Supplementary material for: Barriers to integration of passive screening for sleeping sickness in Bibanga Health District, Democratic Republic of the Congo
Source: PLoS Negl Trop Dis. 2026 Apr 8;20(4):e0014179. doi: 10.1371/journal.pntd.0014179 (PMC13089886; doi:10.1371/journal.pntd.0014179)
Supplement: S5 File — (ZIP) [file pntd.0014179.s005.zip › S5_Verbatim transcripts/5_BCZ_DPS_PNLTHA/AUD.33_ENT_DPS.docx]

**INTERVIEW WITH DPS KASAI ORIENTAL EXECUTIVES**

**Audio N°33: Interview with the Head of the Technical Support Office**

**I. Perceptions on the Integration of HAT Activities into PHC**

**How do you assess this process of integrating HAT control activities into the PHC services of the Bibanga Health District, five years after its implementation?**

*Well, integration is actually a very good thing. I believe that at the provincial level, several zones were affected by HAT, but Bibanga had a particularity. At the beginning of this integration, I think the number of cases, as you know, had significantly decreased, and at a certain point, there was a resurgence. Perhaps we need to understand that HAT care was expensive at the time and was largely based on injectable treatments. Possibly, regarding active case finding within integration—was active case finding being done correctly, or were we waiting for patients themselves, in the event of a problem, to come to health centers? We need to look at things from that perspective. Another thing, another factor probably, is that at the beginning, during this initial enthusiasm for integration, active case finding was much more sustained, and then progressively, there was likely a relaxation. That is how we might understand this resurgence in case numbers. But I believe, if you recall the history of this disease in the country, the fight with mobile units back around independence was targeted, and when the disease was controlled, there was a relaxation, which created a resurgence. Are we not experiencing the same phenomenon? This means we integrate, we strengthen the capacities of health centers, we mobilize the population; in the early stages, there is hyper-motivation, and then gradually, people relax. Is this not the same phenomenon? So, we really need to look at things from multiple angles.*

**Can the success in the fight against HAT in the Bibanga Health District today be attributed to the efforts of first-line healthcare providers? Explain your answer.**

*That is what I am saying. I made a comparative analysis. What happened back then, during the transition from the colonial era to independence, we experienced practically the same phenomenon. But here, we thought integration would allow us to control the disease. This means that integration implies there is no longer a specialized unit responsible for patient care. Instead, there are various operational health structures, which are the health centers, that could already begin to ensure patient care. That concerns patient care. In this integration, as I mentioned, were the same techniques, strategies, or approaches given to the community for active case finding? Because if we set aside the mobile units, this means the activity carried out by the mobile units had to be compensated for internally. Internally, we know that it is more the community health workers. Are the community health workers doing, continuing to do the same work at their level? Because at the community level, we have community health workers organized within CACs (Community Animation Cells), and we have CACs at all levels. Is this work being done? Is this work being done? Now, if you recall well, the mobile units went out for active case finding; understand, the CAC can do the same work, but screening is done at the health center. This means the community health worker continues to do active case finding in the community. You know that in a community, in a health area, depending on the demographics, we can have several CACs, and each CAC has a well-defined geographical area. Is that work being done? Because if that work were being done, we could easily identify cases, bring them to the health center for screening, and if the case is confirmed, provide early care – that question needs to be asked at that level. Also, integration implies ownership. Is there this ownership? Can we say that at the beginning, with the decrease in case numbers, there was ownership, and then a relaxation? These are questions that need to be asked. Another question might be, this is also a natural phenomenon. Perhaps at the start of integration, there was care and sufficient awareness with active case finding, and this active case finding helped sensitize the entire community, and everyone watched their neighbor, and over time, there was relaxation. Does the same dynamic continue? Are we still doing vector surveillance at the zone level, at the community level? Is the community truly involved? These are all the questions. Because integration implies a reduction in the resources that were directly linked to the fight against the disease, you understand that a bit? And everything is merged into the health zone. To such an extent that the few resources arriving for an activity X, when it is community-based, it takes all aspects into account. Is the same motivation there? No. So we need to look at these problems on several levels. Are the community health workers actually doing this active case finding? We can say yes because the number of cases is increasing. If the number of cases is increasing, it means someone brought these patients to health centers so that they could be detected. That means active case finding continues, but now, why are there cases? Has the vector been controlled? Is the vector not still circulating? Because if the vector continues to circulate, then the disease will always be present. So for me, I suppose that starting from a decrease in case numbers and then an increase, these cycles sufficiently prove that at the time of the increase, it means someone identified these patients? The patient cannot identify themselves; they were brought to a structure, which shows that since mobile units are not there, it is the community that brought these patients. It means the community continues to be aware of the presence of the disease. But for me, I see the problem at the vector level. Have we defeated the vector? Is it not the presence of the vector continuing to circulate, a multitude of vectors, or is it the same vector circulating in the community that continues to transmit the disease? Isn't this the level at which we need to look at things?*

**In your opinion, could we already consider reducing, with the aim of definitively stopping, the HAT control activities carried out by the National HAT Control Program (PNLTHA) through its specialized structures to leave room for PHC services?**

*Well, I think we can, because the verticalization of the fight had an objective. We can do it, but we cannot stop abruptly. I think we can strengthen community-based surveillance, strengthen community-based surveillance with clear terms of reference, and once cases are detected, they can be referred for confirmation. If we further strengthen this community-based surveillance, the mobile units could perhaps return once a semester or once a year just to act as an external eye to see if this community-based surveillance has truly been effective or not. Because, considering the resources that mobile units bring, they are not the same as those available for community-based surveillance. But I think if we strengthened it further—the advantage with the community, if this surveillance is sustained, is that it covers all areas simultaneously, permanently, and cases could be detected if they exist, earlier, you see? It could be detected earlier. Now, to ensure the effectiveness of community-based surveillance, we could send mobile units, perhaps once a semester or once a year, just to do a sort of cross-check to see if, beyond what the community has done, there aren't any other cases that went unnoticed. But for me, I think the decrease in case numbers should not only be linked to the mobile units and their visits, no. It should be linked more to the movement of the vector in the community. Because even if mobile units go there, if the vector is defeated, there will be no more disease. This means that if there are patients, it's because the vector continues to circulate. So we need to act more on the elements that can create a barrier to the vector. We need to act more; therefore, at that time, it's particularly about strong sensitization regarding the use of bed nets in households. Now, with the malaria program already, we have bed nets. Does the community use bed nets? We might think yes in households, but when they go to the fields—because that's where there is more vector mobility—when they are in their fields, how does it go? No one knows. That's where I would like us to answer the question: among the new patients, who are they? Who are they? Do we have the characteristics of these new patients? If we manage to trace the epidemiological profile of these patients, we can better understand what happened. Are these patients who stay permanently in the village, or are they patients who move between other zones or field areas? We really need to see what the epidemiological profile of these patients is. It can really guide us because, for me, the primary objective of mobile units was to search and detect for early care, to avoid the spread of the disease and also to avoid deaths related to the disease. You see a bit. And that was the added value of these mobile units. So we can achieve that element with community-based surveillance. But have we defeated the vector? Because if the vector continues, whether it's mobile units or surveillance, we will always have cases as long as the vector is present. So we need to look at it now. I think we need to multiply strategies. On one hand, we do surveillance; surveillance allows us to know if the health problem persists, but we also need to see what causes this health problem. Now, we know it is mostly the vector. Is the vector defeated? But if the vector continues, we can continue with surveillance; we will only realize that the incidence will always be there. The incidence will persist no matter what, but once we stop the vector, everything will stop. So I think we need to combine approaches in this fight. We really need to combine approaches. So, strengthen community-based surveillance. The mobile unit can, for quality control reasons, come once a semester or once a year and see a bit what other means exist to counter the vector.*

**In your opinion, if we want to sustain the integration of HAT control activities in the Bibanga Health District, can we already consider completely ending management by the PNLTHA to attribute it to the Health District Management Team (BCZ), or is parallel management (PNLTHA-BCZ) necessary?**

*As I have always said, the unit of integration for all interventions we have within the system is the health district ; it is the unit par excellence. All programs come together at the health district level. So now, the health zone must help us. I am convinced and I know the zone has been sufficiently strengthened through its structures and its management team. The work done by the mobile unit, I suppose it is on behalf of the program, because I am not in favor of letting go too early. So perhaps we need to reduce the capacities, you see, the capacities, perhaps avoid having a dedicated treatment hospital here in Dipumba like we have. Leave this care at the health zone level, through hospitals, through health centers, because now we have oral treatment, we have rapid diagnostic tests (RDTs), which already facilitate many things. So with oral treatment, the patient can even take it at home. Therefore, now the program, through the mobile units, will only handle what I might call quality control aspects to ensure whether what the zone is doing is on the right track or if there are still many errors or things to correct. And progressively, we will see how things go, and if we need to let go, perhaps we should give it five or ten years to avoid experiencing the situation of 1960 again.*

**II. Factors Hindering (Obstacles to) the Integration of HAT Activities into PHC**

**In your opinion, what are the obstacles to integrating HAT activities into PHC:**

- **At the community level?**
- **At the health service level (Health Centers and General Reference Hospital)?**
- **At the Health District Management Team (HDMT) and Central Level (ECP) level?**
- **At the National HAT Control Program level?**

*At all three levels first—management team, health centers, communities—it is awareness. That is the first obstacle. If there is no awareness that the work we do, the few resources we receive, we must not limit ourselves to X program just because it provided the resources to cover other aspects, that is already a blockage. Without awareness, everything is mowed down.*

*Now at the community level, the first obstacle at the community level, based on what I have observed in several zones, is the lack of ownership. The community fails to take ownership of the activities. After all, we define the community health worker first as a volunteer. This notion of volunteerism is actually misleading. You are always told "volunteer," yes, we did this, what have you brought us? For me, this volunteerism is a truly misleading concept. The question I also ask myself is, can we talk about volunteerism—understand me, I am an economist—can you talk about volunteerism in a community living in such a state of advanced poverty as ours? These are questions I always ask myself as a health economist, and I sense it. When I go into the zones—I have traveled through almost all the rural zones—they will always tell you, yes, we did this, but what did you bring us in return? That is the second obstacle. So we must not believe that this community, even though we selected community health workers, volunteerism is an empty notion, emptied of its meaning by poverty. So this community, when we engage with it, requires motivation, and that motivation must be financial or material. That is an obstacle. You even mentioned it at the level of the management teams, your stories there, as you expressed. What does it mean? The resources you bring, you do not give them to the management team; you give them directly to the concerned providers, which is quite logical because you pay the one who performs the service. So you understand this financial obstacle that we cannot express to you directly so that you do not say that you work only for money. Because the management team is obligated—since it is an administrative duty—to do this work. But to show their dissatisfaction, they sabotage, they don't work.*

*The third obstacle is compliance with standards and guidelines regarding human resources. Because if we could place the right people in the management teams at the right time, I think we would avoid many things. I am not going to praise certain provincial executives; there are doctors who, when you ask for a service and give them a deadline, they respond because they think, "It's my duty, I must work." Because we make uncontrolled assignments—not to say politico-tribal assignments—and now we place people in positions, and the consequence is that we have no results. Because you, Jérémie, arrive somewhere, the person there thinks they are under the protection of so-and-so; anything you ask, they might not do because you have no binding measure, none. I think, in my humble opinion, if we can act on the financial aspect, the resources, on the competency aspect, it might—I am not saying it can solve everything—but it can improve things somewhat. Because people no longer feel accountable.*

*There are two other obstacles I didn't mention. There is this aspect of spirituality that leads us to traditional medicine and churches. Because churches and traditional healers can, at that level, if they are not sufficiently sensitized, block things. They constitute a blockage for me. How is the first reflex in our communities, especially rural ones, as soon as there is a health problem? You know there are multiple interpretations surrounding illness. There are multiple perceptions of illness in our communities, and generally, if one is not in a church, one goes to traditional healers. At this level, if traditional healers or men of God in churches are not sufficiently sensitized, it already poses a problem. But this is where I say—you understand when I spoke about volunteerism—because when you go to rural areas, the community health workers play a crucial role. They play a really crucial role, and we often use teachers and pastors. Where there are informed pastors, they can understand that this situation requires that I take this patient to the hospital because it could be... So we must also include traditional medicine and churches in the group of obstacles, because they constitute the primary places of sequestration, and generally, patients arrive to us in an already advanced state.*

**In your opinion, can the reduction of resources constitute an obstacle to the integration of HAT activities? How can this be avoided?**

*That is what we must fear. That is why I say we must proceed gradually. For example, for the laboratory, we must keep the laboratory for quality control. There should also be supervision from the higher level that would come with perhaps one or two mobile teams to do the same work, to compare, to see if we are on the right track or not. Regarding resources as well, that is why I say this has added value. It is that even if HAT hasn't sent resources and a Community Animation Cell (CAC) has received resources for, say, community-based surveillance for a specific pathology, this community can take advantage to do everything else. This is where we now need to make the management teams—especially the management team, in this case, of the Bibanga health zone—understand that the resources arriving need to be capitalized on to extend surveillance. You can have resources for family planning, for example for DBC, you can have resources for an IVA campaign as happens here. These resources should not be used only for the IVA campaign; they should be leveraged at the level of the Community Animation Cells to say, while you are doing that, also take the opportunity to do community-based surveillance for such and such a pathology. But we don't feel this, we don't grasp this. Especially with these payments by mobile money, it becomes complicated because now it becomes a matter of appropriation by the one who has their phone, who received the money. They say, no, I received this to do this activity, I limit myself to that, whereas it shouldn't be like that. This is where we are losing a bit of this notion of integration. So the community health worker who goes into the field should not limit themselves only to malaria, only to HAT; at the same time, they should address HAT, they see, they look a bit at AFP (Acute Flaccid Paralysis), they look also at yellow fever, they look at other aspects—yellow fever, measles, etc. But if we stay narrow, it means we haven't yet understood the notion of integration very well. That is the greatest danger, and we need to go towards the logic of true integration. For me, the unit of integration par excellence is the health zone, truly the health zone. All programs that arrive come in through the health zone. Even if the resources are not given directly to the health zone, these actors who receive these resources must understand that when we go into the field, it is for one, two, three, four things. I seize these resources; they simply allow me to move, and when I arrive in the field, I must do one, two, three, four. This notion must be well understood at that level. Unfortunately, sometimes we have small issues regarding this.*

**III. Factors Favoring the Integration of HAT Activities into PHC**

**In your opinion, what are the elements we can rely on to improve the integration of HAT into PHC?**

*No, I think we only need to reverse the blocking factors. We need to turn them into positive factors. So, at the community level, it's sensitization, sensitization, sensitization. Show the community health workers that when you sensitize, you must sensitize for one, two, three, four things. Because they only serve as a bridge, and the message they carry to the community is given to them by us, the health system. So it is up to us. This is where I mentioned the competency problems also at the management team level. The nurse doesn't have a problem; they are in their structure, they wait for the patient to be brought to them, and they treat, that's all. So if we, at the community level, can act on sensitization—and to sensitize, we must find key focal points: the houses of traditional healers, the churches. So we must reach the traditional healers, we must find the village chiefs, we must reach the church leaders. At that moment, we give the right information. Not only saying we limit ourselves because we are talking about HAT, no. We talk about HAT, we talk about tuberculosis, because tuberculosis has resources at the community level, with several donors bringing resources for sensitization. Why not take advantage of the RECO resources for tuberculosis to also say, "Attention, when you see someone presenting such and such an effect, such and such a problem, bring them to the center"? Why not use that? So, just with the resources for tuberculosis sensitization, we can already do a lot without necessarily waiting for them to come from HAT. Because tuberculosis, HIV/AIDS, they have resources. Why not seize those resources? Why not seize those resources? Now, who must initiate this? It is the management team. And if the management team initiates—this is where I talked more about competencies—if the management team initiates, understands that the resources I received for tuberculosis, the community health worker who goes into the field, from you I expect the number of people sensitized regarding HAT, regarding tuberculosis, regarding HIV, regarding respiratory infections, regarding diarrheas, regarding... we will have succeeded. But if the team that should pilot this fails to initiate, the community will only do what it was told to do. The consequence will be that we will find ourselves blocked. So we need to act on these various elements, but the greatest catalyst is communication. If we act on communication, I believe we can... and then maybe the rest is: how to manage competencies? How to find the competencies? That is at our level. Now we need awareness at our level to understand that the people we treat, the people who fall ill, are our brothers, our sisters. We must place people capable of assuming the role required of them. But if we must remain in "tribalism, nepotism, political party" logic, we are killing ourselves. Especially since I have never seen a politician, when they are sick, go look for their brother to get treated. On the contrary, they will look for a competent doctor, a competent nurse. No, they do not look for their brother or sister; they do not look for someone from their political party to treat them. So, if we can understand this in this sense, I believe it can help us a lot.*

**Thank you.**
