# Supplementary material for: Barriers to integration of passive screening for sleeping sickness in Bibanga Health District, Democratic Republic of the Congo
Source: PLoS Negl Trop Dis. 2026 Apr 8;20(4):e0014179. doi: 10.1371/journal.pntd.0014179 (PMC13089886; doi:10.1371/journal.pntd.0014179)
Supplement: S5 File — (ZIP) [file pntd.0014179.s005.zip › S5_Verbatim transcripts/5_BCZ_DPS_PNLTHA/AUD.34-ENT_PNLTHA.docx]

**INTERVIEW WITH PNLTHA EXECUTIVES IN KASAI ORIENTAL.**

**Audio N°34: Interview with the Provincial Coordinating Physician**

**I. Perceptions on the Integration of HAT Activities into PHC Services**

**How do you assess the process of integrating HAT control activities into the PHC services of the Bibanga Health Zone, five years after its implementation?**

*I strongly doubt that this integration was already effective at that time, because I know at least that trainings were organized there, but to speak of integration, it is not only about training, but also about equipment, isn’t it? Did the equipment follow? I strongly doubt it, I strongly doubt it. I know that training was provided, I know that some supervisions were organized, I know that some materials were indeed deposited, but not everywhere. I think that if there was some talk of integration, it was in a few structures, in one, moreover, at the General Reference Hospital (HGR), where things were somewhat faltering. It is only now that I see the HGR beginning to take ownership of the fight a little.*

*But if we are to talk about integration, we should have started with training, so that people first know how to screen. It is done clinically when people come for care in the structures. They should also, based on the signs the patient reports, the signs the patient will present, think of sleeping sickness. There is serological screening done based on Rapid Diagnostic Tests (RDTs), okay, but there is also parasitology. So, for parasitology screening, we have the microscope and other equipment to be able to search for the trypanosome. There is also the case management that we must integrate, because that is also part of the integration package, vector control, and everything. So, regarding the Bibanga health district, after this integration by the Lubumbashi team, when I went there in 2019 to participate in a monitoring meeting, just to see what about the suspicion, is serological suspicion being done, where are we? Because the report was already problematic. There were Integrated Teams (ITs) that were faltering, in the sense that they never… there were those who had never even seen an HAT patient, yet they are an IT in Bibanga. That's why I am saying that it's good that integration took place, but was it truly effective integration?*

**In your opinion, could we already consider reducing, meaning definitively stopping, the HAT control activities carried out by the PNLTHA through its specialized structures to leave room for the Health District Management Team (HDMT)?**

**No, it's difficult. Without the support of the mobile team, it's difficult. It's difficult in the sense that for HAT patients to reach the hospital, they have to be really sick. So it's someone who is already at stage 2 of sleeping sickness, often. Yet, we all know very well that a patient screened at the first stage is a patient who heals correctly and completely. So, saying that we wait for the person to catch the disease, for the tryp to develop, for the tryp to travel throughout their body, that is not good. I do not think that only fixed structures can eliminate it. No, that won't happen, because when they are already sick and continue living their normal life in the community, they will spread the disease. So, instead of treating one or two people, it will be an entire community. So, thinking alone, thinking that only fixed structures can eliminate it, no, it would be difficult. We absolutely need both strategies, passive and active. But there are other things missing. We must also integrate sensitization. Yes, the community should know the signs of sleeping sickness, know what the tsetse fly is, okay, and how to combat it, how to fight against the tsetse fly. This could make a somewhat more robust package to be able to fight HAT within fixed structures. Because the University of Lubumbashi, PNLTHA, they went, it's good, it was the RDTs, it's good. Uh, some structures were trained in parasitology, it's good, but does the community have sufficient information? Do you see a bit? Because if the community has information on a permanent basis, we noticed after a survey that even the memory of the disease had significantly decreased. The survey was done in Ngandajika, the survey was done in Kasansa and in Tshilundu. There are people who do not even know the disease, huh, huh, we have already heard. Even, I told you about the ITs, didn’t I? I said who has already treated an HAT patient? No, no, there weren't many. Okay, I understand that, but who has already seen someone suffering from sleeping sickness? There, I think out of the seventeen ITs, there weren't even seven. Uh, if someone, the IT themselves, has never seen it, they will very easily miss it. But what about in the community? Especially since there aren't many cases anymore.**

**I come back to tell you that for me, passive screening alone will not suffice. Now, if the mobile unit is there, they find suspects, they send them to the structure, follow-up is done, and everything. I think that could solve the problem.**

**II. Factors Hindering (Obstacles) the Integration of HAT Activities into PHC Services**

**In your opinion, what are the obstacles to the integration of HAT activities into PHC services:**

**At the community level?**

**At the health service level (Health Center [CS] and HGR)?**

**At the Health District Management Team (HDMT) level? and Provincial Management Team (ECP)?**

**At the national HAT control program level?**

*Okay, your work concerns the difficulties, the bottleneck, uh, what prevents integration from being effective.*

*Integration is problematic in the sense that concerning the disease, sleeping sickness itself is a neglected disease, especially since there are no funding sources that follow. I'll take an example, I was in a structure and they told me, Doctor, when we do a malaria RDT, someone pays 500 francs, but your HAT RDTs there, we get paid nothing. That doesn't motivate, you understand? That's a bit of it, which means the person can even forget that I don't have HAT RDTs, it's not their problem.*

*Regarding the Bibanga health zone, which is a pilot health zone concerning integration. What could be, what I can call the obstacle, is firstly at the level of the ITs, it's this instability of staff. You train, you equip, and the person leaves. As soon as they leave, we're back to square one. So this instability really constitutes an obstacle, huh? That's number one. Number two, uh, money, the funding problem. Sleeping sickness is a disease that affects the poor population, and we asked them that when someone comes, it must be done for free. So, see, asking someone to work for free doesn't encourage them too much; it really requires a firm will. That too is, is, is an obstacle, the funding. Normally, we could buy this activity, we could buy this, this, the case management or the screening for HAT. So, you examined ten people with RDTs, we pay you something. So, normally, we should have a partner, a partner, any partner, and not only these HAT partners, even the European Union, even PROSANI which has taken over the health zone, they could also include HAT among the diseases that are bought, that are subsidized. That's at the level of the structures, which can serve as an obstacle because in fact they are not paid, they are not motivated, they forget. They are too ready to refer when the patient comes, ah ah, you just go to that structure, they are not very motivated to manage them. Those are the two obstacles I found at the health center level.*

*At the BCZ, let's not even talk about it. The BCZ has many activities; these are people who are too busy. They are always busy where the money is. If they hear there is no funding, they will do it for a while, then it's over. I think that if we could have a partner who bought these control activities, it could solve the problems. That's at the BCZ level.*

*Then, at the coordination level, what we lack is supervision. You know, we have integrated, but we must follow up very closely. When they know that every month we come to visit, every month we will come to check what I'm doing, every month they will call me for reporting, you see a bit, it's a kind of motivation. But we are not very many, at the office, okay. See, for example, since January, no supervision has been done. That hinders a bit; it's really an obstacle to integration; they will forget very quickly, there you go. And when you arrive, you start from zero. You see, for example, when I learned that there were RDTs at the Provincial Health Division (DPS), it shocked me. Right after, I will try to call the head doctor to tell him that RDTs are not meant to be kept at the warehouse; they are to be sent to the structures.*

**Could you describe the supply chain for RDTs to the structures by the coordination?**

*The question you just asked, about knowing what circuit the RDTs should follow: Often, we use two circuits. The first is official, meaning the structure makes a requisition; when it arrives here with the signature of the Health District Medical Chief (HDMC) or the Medical Director (MDH), it comes, and we supply. The second circuit is the mobile unit, and it's the circuit that is most profitable for us. Because giving out RDTs is good, but getting reports back is another thing. You can give them out and start, but we had this many RDTs; we gave them to whom? You understand? So, when it's the mobile unit, the mobile unit knows that I, leaving the coordination, had, for example, 100 RDTs. I know I gave ten to the HGR Bibanga, five to such and such a health center, and so on. So, at the end of the month, we must go; I gave five, the report. But your report is how? How many RDTs do you have left? If he starts asking again, I don't have any more. But I gave you five, and you only tested two people, what did you do with the other three? You see a bit. So, the circuit via the mobile unit is the circuit that is much more profitable for us. Much, much more, how can I say, uh... beneficial, interesting.*

**Is there another source of supply other than the one used by the coordination?**

*Actually, we have a new partner, ENABEL, which is concerned with integrating HAT into the horizontal health system. So ENABEL wants to work with the DPS; it supplies the health zones with inputs via the DPS. And I was even shocked to learn that there are RDTs still sitting there in their warehouse; they will expire. When we send them like that, it's so that they send them to the structures that were trained, or so that they implement what they are integrating. But if they are still keeping them there, that's shocking, because after receiving them, they were supposed to dispatch them, they were supposed to send them to the health zones, they were supposed to give them, at least to the MCZ, who know the structures that were trained, because ENABEL also trained some structures. We have Kasai Oriental, ten, Lomami, I think five if I'm not mistaken. So for the ten in Kasai Oriental, they should have given them out; why are they still keeping them? You see how the blockage is at all levels, you see a bit how that can expire. But there are some at the DPS. Recently, there was a provincial review at the DPS, and a health zone medical chief asked me, Doctor, isn't HAT integrated in my health zone? I said, but that's not true, I was with your laboratory technician. When he realized that integration is not only about microscopy, he said, ah okay, I thought it was the whole package and everything. If I had known at that moment that there were RDTs idle, I could have taken action. Now, I want to see the head doctor so we can see how to dispatch these to the structures.*

**In your opinion, what are the reasons for stock-outs of HAT inputs in the structures?**

*Let's take a very basic example with the RDT. Recently, in March 2022, I was at the HGR Bibanga. I entered the laboratory, and they said to me, no, we don't have RDTs. I said, okay, but the zone's Administrative Manager (AG), the hospital's AG, no, the health zone's AG was at the coordination. I myself signed the dispatch note. Let's go; we arrived at the pharmacy. The RDTs are there in the pharmacy waiting for someone to requisition them. In the hospital, uh, the hospital laboratory did not requisition the RDTs from the hospital pharmacy to go and test. You understand? That's, how am I going to interpret that? It's, it's, it's negligence, a neglected disease, neglected disease. And it's not the pharmacist who will force the laboratory to take the RDTs. For them, there are some at the lab, but the lab didn't come to get them.*

**Do you sometimes experience stock-outs of RDTs while providers need them?**

*Yes, at one point, but it didn't last long. We had some stock-outs until 2010, 20 or 21. We had some stock-outs, but it didn't last long, it didn't last long. And actually, we had RDTs close to expiration. Okay, so to avoid expiration, we cleared out the coordination warehouse. And before we were resupplied, it took some time for us to get those inputs.*

*But I gave you the example of the HGR with RDTs in the pharmacy, but at the laboratory, there were none, and they said, no, we haven't done RDTs; it's been this many days, we have no RDTs. I said that's not possible; we gave out so many RDTs. But imagine, if we had not signed the dispatch note, for example, or if I had forgotten because I am also human, I can also forget, the RDTs would have remained in the pharmacy and expired, and at the HGR, they wouldn't have done RDTs. That's a bit of the reality on the ground.*

*I remind you again that HAT is a neglected disease. The central office is a unit that manages all pathologies, not just HAT; there is malaria, there are vaccines. I take malaria for example, they have medicines, they have RDTs, patients pay, even though it's supposed to be done for free. But there, there is a lot of interest. Where there is interest, people work. Doing this, if they remember, they do it, but otherwise, it's not easy.*

**In your opinion, how could the reduction in resources from the PNLTHA influence the integration that has already begun?**

*Yes, but that's where we are heading. We have reduced activities, and that's what we are seeking, the resurgence of the disease. That's what scares us. That's what scares us. You know, this disease, once, I got scared was in 2020, during the COVID lockdown. Kasai Oriental had, in one day, eight patients declared behind Katanda. I myself, not that I was told, I was on the ground that day. If they had sent me the report, I could have said, ah, maybe they didn't see the tryp correctly. I myself, on the ground.*

*I saw eight, eight, eight HAT patients with trypanosomes behind the health center. At one point, we had a clinical trial; we were looking for patients to include. The agents or staff of Katanda scoured the town, scoured the villages, to look for patients, and they didn't find any. But behind the center, we were at the center, we left, we followed, we worked at the center, we finished, we said the mobile unit is not far, we went on foot. And that day, I was scared. I said, ah, so one day I could be sitting like this and be told there are 50 patients somewhere.*

*III. Factors Favoring the Integration of HAT Activities into PHC Services.*

**In your opinion, what are the elements we can rely on to improve the integration of HAT into PHC services?**

*What I propose so that integration, uh... can be improved, for me, is the involvement of everyone at their own level. So that the IT has the concern, okay, to screen even clinically, to think of the disease. So that people do not forget sleeping sickness, it's true it's neglected, but that they make an effort. That everyone at their own level can get involved. There is the IT doing their part. There is first the community doing their part, because it starts with the community. The community suspects cases and refers them to the center. The community, you see, organizes vector control, okay. And when someone who goes to the field properly, we see them, they become weak, the community is alerted about this. I think if everyone did their work at their level, we could talk about good integration. The problem is that we forget; as soon as the coordination turns its back. The coordination also has its duty to be in the field all the time to support. You see, when you know the coordination will come to supervise us, you see, when you supervise, someone starts looking at you, no, but it's because it's been a long time, we weren't told that. That was at Muya; someone answered me like that, but Doctor, you don't come anymore. We have, uh, but we didn't tell ourselves that we have to come every day. When we come once, normally we should supervise a fixed structure twice a year. But how many do we have? Up to now? We have integrated into at least 68 structures. Now, visiting all 68 structures twice, it's very complicated. But if everyone had that awareness, the awareness of being a health worker, the community here would know that we are in an area endemic for HAT, we must fight by organizing vector control, by sensitizing the community during home visits by community relays, during meetings of the Health Center Management Committee (CAC). Among the points they list, they should also talk about sleeping sickness.*

*At the BCZ level, that the supervisors also integrate, the HAT supervisor into their integrated supervision. Because you see the supervisions that are done now are integrated; we also include a theme concerning HAT, and often regularly, I think that will advance or improve things.*

*There is also the central level that must send us reagents. Send us the RDTs in real time, not when we have run out, we have asked, asked, asked, or we start taking two here, like that, no. We need them; we say our alert stock is at [a certain point], and we are resupplied at any time.*

*So, that everyone does their part. I think that can really, we can have good results concerning integration.*

*Another aspect, uh, uh, it's that we are being promised that we will integrate HAT indicators into the DHS2 (Health Management Information System). As soon as we integrate that, it will be easier. It will be a routine activity. So just as the IT reports on diarrhea and on Acute Respiratory Infections (ARIs), so too, they will report on RDTs performed, the number of suspects. If we take all these indicators and put them in the DHS2, it will be very easy, even from my computer. If I enter the DHS2 of the health zone, I get my information. It will be the same for the base, at the BCZ, at the DPS, and for me too.*

**Thank you.**
