# Supplementary material for: Barriers to integration of passive screening for sleeping sickness in Bibanga Health District, Democratic Republic of the Congo
Source: PLoS Negl Trop Dis. 2026 Apr 8;20(4):e0014179. doi: 10.1371/journal.pntd.0014179 (PMC13089886; doi:10.1371/journal.pntd.0014179)
Supplement: S5 File — (ZIP) [file pntd.0014179.s005.zip › S5_Verbatim transcripts/5_BCZ_DPS_PNLTHA/AUD.35_ENT_BCZ.docx]

**INTERVIEW WITH EXECUTIVES OF THE BIBANGA HEALTH DISTRICT**

**Audio N°35: Interview with an Executive of the Bibanga Health District Central Office**

**I. Perceptions on the Integration of HAT Activities into PHC Services**

**How do you assess this process of integrating HAT activities into the PHC services of the Bibanga Health District, five years after its implementation, in terms of success or failure?**

*Thank you, Papa Jérémie. Regarding success first, this is an initiative that has greatly helped us in terms of approach. We have reached a stage where every facility is expected to conduct screening for early detection and management of sleeping sickness. Indeed, this is a real success. Since all facilities are integrated into screening and some also into case management, this truly represents a successful system for our sleeping sickness control activities. Additionally, there are successes regarding the community-based approach. We received traps that the community itself monitors. They take turns, install them, and check for flies. We continue to strive for sleeping sickness control. That is a real success. We also have RDTs; I believe in our health district, there were few facilities that performed screening before. But this time, we have integrated the entire health zone. We did not have reading rooms or mini-columns, but we received equipment and also reagents for screening and confirmation of sleeping sickness. That is a real success.*

*Regarding areas for improvement, one is the availability of RDTs. Sometimes we experience stock-outs, so there are no RDTs for facilities that can perform early screening. Apart from that, I believe control efforts are ongoing. The facilities are doing the work.*

*Another success of this approach is the reduction in the number of cases. This also constitutes a success because when we screen in time and manage cases promptly, people can truly be saved from sleeping sickness. That is a success of the approach.*

**What explanation does the health zone provide to justify the resurgence of the disease in 2020, given that the trend was declining with only one new case in 2019?**

*Thank you, Papa Jérémie. We are aware. Regarding the cases we had in Katanda, specifically in Bufua. The eight cases screened by the mobile unit, there are people… We follow the algorithm. People may come to the health center, but the provider must follow the algorithm to detect and determine if it is a case for which HAT RDTs should be performed. It is not done systematically for everyone; there are cases according to the algorithm that people can perform and then identify. However, there is a slight disregard, related to the community itself. Even if you raise awareness in the community that we have a screening center, if you go there we can screen, we have RDTs and so on, the community's level of understanding requires behavior change. It would take awareness campaigns to address this. These are cases where people even said it was a disease caused by sorcerers. They said, "We cannot go for screening because if I get screened and find out I am positive for sleeping sickness, that would not be good." But these were cases within the same family, in the same household, where we found the eight cases. We had found one case and informed them they could come to the center for screening, but these people refused to come to the center. However, with the visit of the mobile team and their awareness efforts, that is why they went and we detected these cases.*

**II. Factors Hindering the Integration of HAT Activities into PHC Services**

**In your opinion, what are the obstacles to the integration of HAT activities into PHC services at the following levels: Community level? Health service level (HC and GRH)? Health District Central Office level? and Central Pharmacy level?**

*Thank you. I believe regarding… Let us start with the GRH. At the GRH, we can screen and confirm, but for management, the patient had to go to Katanda. But you see someone coming from, say, Nsengiyabu directly to the hospital; we confirm the case and tell them, "Go to Katanda for management." This costs them dearly because they think that when they go to Katanda, they will still need significant resources to support their stay there. One obstacle is distance for patients. Regarding the Central Office level, there is the issue of follow-up. For case follow-up, there are patients lost to follow-up. Someone can come today, and you tell them they are positive, and they say, "No, let me go prepare myself first and come back so I can go for treatment." When they leave, we lose the case; we cannot find them again to say the case came from such and such place. We may go there, and they say, "No, he is no longer here; he moved." All these are obstacles because when someone is already tested positive and remains in the community, it is still a risk to the community. It is a risk.*

*At the health area level, we can detect a case and say, "No, you go to such and such place for confirmation, and if positive, you will be managed free of charge." I give you, for example, the recent case in Kalunda. The woman came to the GRH; we screened and found her positive. We asked her to go to Katanda for treatment. We even told her the treatment is free; you pay nothing; you will be given food; everything is there; take the medication so you are not a factor of contamination for the population. We made some noise about it. She asked for means, transport means to leave her home and get to Katanda. That too, transportation costs. But we did everything to convince the family, and we took her to Katanda. She was treated. She is cured; I believe last time she came back for follow-up. That is essentially it at the health area level. Even at the community level, at the health area level, as we speak, we are really in RDT stock-out. So there may be cases that we miss during this stock-out period, which we might not know about even when the mobile unit passes and can detect cases; that is also a problem. Another problem is awareness. We must raise community awareness so the community has knowledge about sleeping sickness.*

**Are you aware of stock-outs of HAT supplies? What explanation could you give for this weakness in supply?**

*Thank you. Regarding stock-outs, yes. Regarding the stock-out aspect, it starts first at the base, the health areas, because it is the health areas that consume the RDTs. They must calculate the average monthly consumption of the RDTs they use. When they see that the quantity they have is insufficient, they can place an order, not waiting until two or three months later; they place the order. But sometimes we face constraints because they only inform us when they are in stock-out. They will tell you, "We are out of stock." Or when you visit for supervision, you ask, "Do you have HAT RDTs?" They say, "No, what I had is finished." But it is finished because they should have placed an order. There is an emergency order we can make. You say, "No, here, I don't have that, so I placed an emergency order." They can supply us. And that is the issue. If they placed the order before, because we must not wait until there is a stock-out to place the order; during the time it takes to place the order, request the RDTs, and get supplied, during that time, people can get infected. This is essentially about the structures. If they submit their order in advance, they tell us, "No, here, I do not have HAT RDTs." Then we only place the order, "No, we need RDTs." We do this every time here. If someone comes here, we give them some of the order, and they bring us the RDTs. But this is a difficulty or a pathway for the health structure.*

**In your opinion, how can the lack of provider motivation or financial support influence the integration of HAT activities in the District?**

*I believe this is our core or routine activity, so to speak. Even when there is no funding, we will always ensure it because this is among the diseases that must be closely monitored to prevent the population from experiencing more cases. I believe we do not work only for money; we also help the community. For us, regarding the community, it is about awareness. Even if there is no money, we say to ourselves: this disease kills; if you do not get screened, that is it. Even if there is no money, people will always do it. We must always do it because if the population is affected by this disease, it becomes a burden for the central office. So we must always raise awareness and also change the community's behavior. This is our activity; even without money, we must always take responsibility. But if motivation for HAT is needed, I think it is mainly our ITs, the ITs really who have nothing. Since we work and receive nothing, if people receive something, even just for monitoring at the base, so they know that this activity is truly supported at the office level, there would be some encouragement.*

**III. Factors Favoring the Integration of HAT Activities into PHC Services**

**In your opinion, what are the elements we can rely on to improve the integration of HAT into PHC services?**

*Well, for improvement at the health area level, and even the community level, improvement always lies in awareness. I do not think that if we give people something today, one day there will be none. But first, the community must understand that this is a problem for them. When the disease is present, it is the community that suffers. To avoid suffering, the community itself, including the ITs, must take ownership of this activity.*

*Regarding awareness campaigns, behavior change, home visits where we always talk about HAT: what are the consequences? What are they? That is how we can truly achieve concrete results and maintain momentum for this activity.*

*At the Central Office management team level, it is about supervision. I look at the checklist we have for integrated supervision; when I personally reviewed it, because I have a checklist but I left it, in that checklist there is no HAT section. That is in the specific checklist for integrated supervision; we have not integrated HAT activities. Those who go for supervision must review all activities taking place at the health area level, but there are supervisions conducted without addressing the HAT aspect. My major concern is to train all supervisors on the management team regarding the HAT approach. Then, I believe everyone will integrate this aspect, even during supervision; they will go with the same understanding regarding HAT.*

*Another aspect is motivation related to HAT. If we could motivate, it is especially our ITs who really have… we work and receive nothing; we work and receive nothing. But if people receive something, even just for monitoring at the base, so they know that this activity is truly supported at the office level, that would help.*

**Thank you.**
