# Supplementary material for: Barriers to integration of passive screening for sleeping sickness in Bibanga Health District, Democratic Republic of the Congo
Source: PLoS Negl Trop Dis. 2026 Apr 8;20(4):e0014179. doi: 10.1371/journal.pntd.0014179 (PMC13089886; doi:10.1371/journal.pntd.0014179)
Supplement: S5 File — (ZIP) [file pntd.0014179.s005.zip › S5_Verbatim transcripts/5_BCZ_DPS_PNLTHA/AUDIO.32_ENT_CUM.docx]

**INTERVIEW WITH CUM TSHILENGE**

**Q1) Sir, you are the team leader of the specialized team (EM) of the PNLTHA. Could you tell us how many Health Areas (AS) of the BIBANGA Health Zone (ZS) your Mobile Team (CM) covers in active screening activities?**

***R:****Thank you very much. Indeed, my Mobile Team (EM) covers a part of the BIBANGA Health Zone, the northern part. I cover four Health Areas: KABALA 1, KABALA 2, CIBILA, and a part of the BIBANGA Health Area.*

**Q2) Are you aware that the Health Areas of the BIBANGA Health Zone that you cover had integrated Human African Trypanosomiasis (HAT) control activities that were implemented in their Minimum Activity Package (PMA) between 2016 and 2018?**

***R:****Yes, I am aware, because there was a team that came for the integration. Moreover, the four Health Areas in our operational area have been covered since that time.*

**Q3) The BIBANGA Health Zone, which was once a hyper-endemic focus of HAT, now reports only a few rare cases per year. Could you tell us about the efforts made at different levels to achieve this situation of decline?**

***R:****Yes, indeed, we commend the program for everything it has done. In the northern part of BIBANGA that we cover, the EM visits the villages. We travel on foot to reach these villages. Thanks to the means provided by the program, we manage to reach them. We often travel by waterway using an outboard motor, and sometimes, when it is truly inaccessible, we have the community transport the equipment to reach the scheduled village. That is how we managed to eliminate [cases]. We also had support from MSF, who came to assist us in carrying out control efforts in these hard-to-reach villages. As a result of these actions, and also with the integration of activities into all Health Centers (CS), there has been this significant reduction in cases in BIBANGA. In any case, if things continue in this direction, we will eradicate the disease in the BIBANGA Health Zone.*

**Q4) Sleeping sickness (HAT) was supposed to be eliminated as a public health problem by 2020. Today, in 2022, your operational area still reports cases. Do you believe in the elimination of this disease in the near future, and with what means?**

***R:****Yes, this is possible if resources are made available to the coordination so that the Mobile Teams can go there. For example, there are these remote areas where, if the EM does not pass for 2–3 years and perhaps someone contracts the disease and enters those villages, it becomes necessary that all these villages be covered with screening. Since there are already Health Centers that perform passive screening, it is essential that the villages be visited by the EM. That is how we can eliminate the disease.*

*Furthermore, for your information, last year the EM reported only 3 new cases, and these 3 new cases all came from BIBANGA. This indicates that there is still a problem. Therefore, all these villages must truly be visited. If resources are made available to the EMs, along with fixed structures that perform screening and refer suspects to where sensitive confirmatory tests are conducted, we will eradicate the disease.*

**Q5) Do you think that a Health Area like CIBILA, which is the largest in the BIBANGA Health Zone, is capable of meeting the community's needs regarding HAT screening coverage with only one screening and confirmation structure based in CIBILA itself?**

***R5)****Yes, I think this issue has already been addressed once in a reprogramming workshop. Regarding the CIBILA Health Area, CIBILA is a very vast area, and almost 90% of its villages are hard-to-reach. We visit these villages starting in the dry season, and even during the dry season, sometimes we have to go on foot or use an outboard motor. There are villages with patients who cannot easily reach CIBILA; they cross the river to go to MUKUMBI because only the riverbank separates them from MUKUMBI. Finding CIBILA too far, they prefer to get treatment in MUKUMBI. That is why we suggested that the HAT Rapid Diagnostic Test (RDT) be integrated everywhere in CIBILA. But the difficulty is—I can say that this is a Health Area where all villages have diamond diggers. This is a population that is not stable; they move constantly in search of diamonds. Thus, it is difficult to have a nurse who stays in a mining area for a long time. That is the only difficulty. We thought about leaving the RDT in KASONGO because there was a nurse we saw who had already been there for 2 or 3 years. Unfortunately, while we were considering that, he moved away. That is the difficulty we have in covering the CIBILA Health Area. But if there is a subdivision with a second structure, perhaps it could work.*

**Q6) Mr. Dieudo, the integration of screening activities has already taken place in all structures according to the standards. The Health Zone is supposed to take over to coordinate these activities alone and ensure supervision without your support. In your opinion, should we let them proceed, or is your accompaniment still necessary?**

***R:****First of all, this is an issue we have consistently decried in reprogramming workshops—the idea that the Health Zone could start supervising these structures, and that reports and everything would go through the Health Zone. We understood there were many difficulties. Now, what do we do as an EM? There were even times when a structure would go 3–4 months without reagents or any inputs, waiting for the Health Zone to bring them. When we realized this had to be addressed, the EM took matters into its own hands. We come—it depends—we visit the structure during supervision. The EM goes there, sees what is missing. Since we have the means to reach them easily, we take the necessary items. If it is RDTs, we bring them, and we ask the staff—since we had already agreed that structures should send reports by the 25th or 26th of each month—we waited for these reports, but they didn't arrive. So we go there, and the report must go through the Health Zone central office (BCZ). We understood that this process was causing significant delays. We started calling them; we first call the person in charge of the structure, who sends us the report by SMS, and the hard copy comes later. This is how we work with them. This way, we can easily monitor to know the stock levels and how many suspects were followed up. Because not all integrated structures have sensitive confirmatory tests; we work with them in this sense. Where there are suspects, we give appointments and visit. If possible, we instruct them to send the suspects to CIBILA so that confirmatory tests can be performed.*

**Q7) What do you think about the continuity of activities in the structures that have integrated HAT activities after your definitive withdrawal of support?**

***R)****It is complicated. You know, the central office has always decried the problem of funding. "Oh, you make us work without money." In this sense, if we, the EM, do not make the effort to collect the reports, we could go a whole year without the structures providing reports, or the structure sends the report to the BCZ, but the BCZ does not know how to forward it to us. That is why sometimes we collect the reports from the structures, and sometimes, when they need that report because they don't have it, they refer to the EM, so the EM can take the report that came from them and send it to them. So this is one of the difficulties in the transmission of reports. The BCZ, when we are in reprogramming workshops, says, "We will do it," but the result is not good. That is how the EM always does everything; if there is a delay—it's been a month without receiving reports—sometimes I leave here with the driver, and we go to collect the reports.*

**Q8) If today you found yourself at the BCZ as a supervisor in charge of HAT, what contribution could we expect from you to improve integration?**

***R)****Yes, if today I were at the BCZ, I would make my contribution in the following way: first, as we do during monitoring, in the SNLS report, sleeping sickness, like any other disease—because we see our colleagues give more attention to other diseases because they have funding and other resources—I would tell them that we should consider sleeping sickness, supervise it, and provide reports on it just as we do for other diseases. Because the BCZ centralizes all diseases, not just some while excluding others. And for the nurses, it is a matter of habituating them to the diagnosis so that they start thinking about sleeping sickness as well, and not only about malaria and typhoid fever, forgetting HAT. This is perhaps what I would think to pass on to the nurses.*
